# Easy access to a carbohydrate-based template for stimuli-responsive surfactants

**DOI:** 10.3762/bjoc.16.229

**Published:** 2020-11-17

**Authors:** Thomas Holmstrøm, Daniel Raydan, Christian Marcus Pedersen

**Affiliations:** 1 Department of Chemistry, Faculty of Science, University of Copenhagen, Universitetsparken 5, 2100 Copenhagen Ø, Denmark; 2 LAQV-REQUIMTE, Departamento de Química, Faculdade de Ciências e Tecnologia, Universidade NOVA de Lisboa, 2829-516 Caparica, Portugal

**Keywords:** carbohydrates, emulsifiers, stimuli-responsive, surfactants, synthesis

## Abstract

In this paper we describe the synthesis of a new carbohydrate-based building block functionalized with azido or amino groups on the 2 and 4 positions. The building block can be synthesized in anomerically pure form in only five scalable steps starting from commercially available levoglucosan. It was shown that the building block could undergo alkylations under strongly basic conditions. The building block with azido groups could furthermore take part in CuAAC reactions, generating derivatives with ester or carboxylic acid functionalities. In addition, the anomeric mixture of the building block was used for the synthesis of a molecule that could act as an emulsifier only in the presence of Zn^2+^ ions.

## Introduction

**Figure 1:**
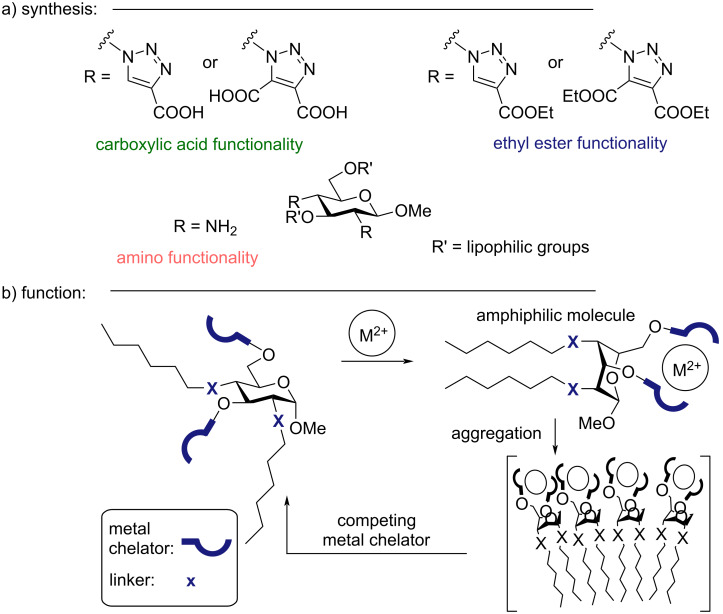
a) The carbohydrate-based building block for the synthesis of stimuli-responsive surfactants. b) The concept of stimuli-responsive surfactants.

Surfactants (surface-active agents) are molecules with both a hydrophilic and a lipophilic domain, thereby rendering them amphiphilic. This dual functionality gives surfactants unique properties such as being able to stabilize emulsions (emulsifiers) or forming large self-assembled aggregates that can take the shape of, for example, micelles or liposomes [1]. The latter property has been of great interest during the recent decades as the self-assembled aggregates have found the way into many fields of research. Within the field of organic chemistry, the smaller aggregates, micelles, have been exploited as a new reaction medium, making it possible to perform organic reactions in water, thereby giving rise to a greener approach to organic synthesis [2–3]. Furthermore, the micelles act as a mini-reactor in which the reagents exist in a very high concentration, giving cleaner and faster reactions [1]. The larger aggregates, liposomes, have shown great potential as drug-delivery system owing to their size and the ability to carry drugs, both in the polar (core) or apolar (lipid bilayer) interior [4–5]. A challenge in liposome-based drug delivery systems is to release the drug at the place of function. This challenge has led to the development of stimuli-responsive surfactants: amphiphilic molecules that can alter their properties upon an external stimulus [6]. The intelligent surfactants are not passive but designed to undergo a molecular change when it is triggered with either a change in the pH value [7–8], upon light irradiation [9] or in the presence of metal ions [10–11]. Common for all of the systems is that the hydrophilic head group can undergo a conformational change upon an external stimulus. The head group is typically functionalized with two lipophilic tails rendering the compound amphiphilic. The conformational change of the head group acts as a mechanical impulse that can increase or decrease the distance between the two lipophilic tails, thus changing the amphiphilic properties of the molecule. Incorporating such molecules into the lipid bilayer of liposomes can result in the decomposition of the whole liposome when the stimuli-responsive surfactants are triggered, thereby releasing the cargo [8]. Guo and co-workers have earlier succeeded with the use of pH-sensitive surfactants and even introduced the term “flipids” for such pH-responsive lipids [7–8]. This term could also describe compounds where the binding of a guest molecule is able to change the conformation of the head group, for example, through a ring flip giving rise to an altered amphiphilicity. Yuasa and co-workers have employed a metal-chelating xylopyranoside derivative as the polar head group [11]. The xylopyranoside was functionalized with two amino groups on the 2- and the 4-position while having lipophilic tails on the anomeric and the 3-position. This configuration made it possible to chelate Zn^2+^ ions only when the pyranoside was in the flipped ^1^*C*_4_ conformation. The binding of the metal ions could thereby induce a ring flip that would manifest itself in a decreased distance of the two lipophilic tails resulting in a self-assembly of the molecules into larger aggregates that could be characterized with DLS and TEM [11]. In a previous study by Yuasa et al., it was shown that the distance between the two groups on the anomeric and the 3-position indeed decreased upon a metal-binding event. This study was carried out by having pyrene fluorophores attached to the two non-chelating positions, giving rise to excimer fluorescence when the distance between them was decreased due to the ring flip [12]. Carbohydrates with *gluco* stereochemistry have also been used as templates for conformational switches but have, to our knowledge, never been used as stimuli-responsive surfactants [13–14]. The compounds would have the benefit of being generally inexpensive due to the high production of glucose. However, going from glucose to a conformational switch often involves time-consuming synthetic transformations employing costly reagents [15–18]. In this paper we describe the synthesis of a new carbohydrate-based building block that can be used for the synthesis of stimuli-responsive surfactants as it has the ability to be functionalized with both metal-chelating groups and lipophilic groups at a late stage of the synthesis (Figure 1a).

The synthesis is centered around the cheap and commercially available levoglucosan, a glucose derivative that can be produced directly from cellulose [19]. This building block seemed ideal for the synthesis of a stimuli-responsive surfactant with amphiphilic properties induced by the presence of Zn^2+^ ions (Figure 1b). In order to synthesize a glucopyranose-based molecular switch, a 2,4- or 3,6-functional group pattern is needed as these positions reside *cis* on the ring, making them pointing in the same direction of the ^1^*C*_4_ conformation. When the 3- and 6-position are tethered, for example by metal binding, the glucopyranose ring undergoes a ring flip, bringing together the 2- and 4-position as shown in Figure 1b. Levoglucosan is the ideal starting material for such transformations as the 2- and 4-positions can be functionalized with high regioselectivity via the Černý epoxide [20].

## Results and Discussion

### Synthesis

**Scheme 1:**
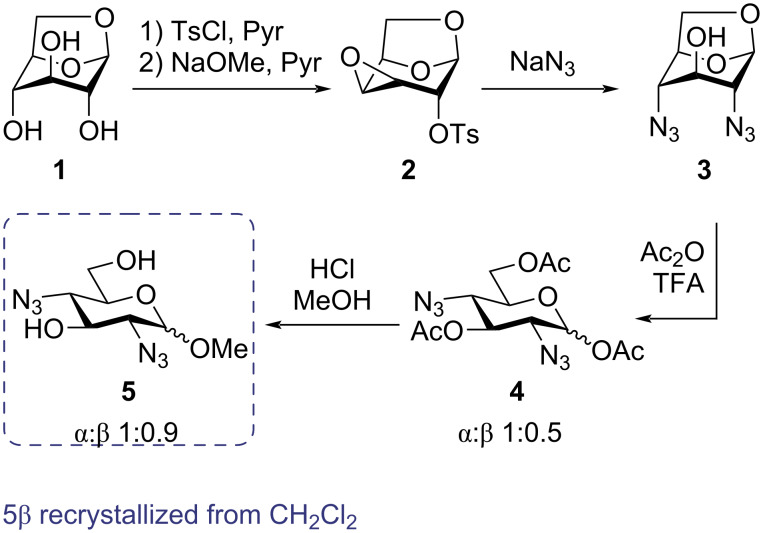
Synthesis of **5** from levoglucosan (**1**).

The synthesis was initiated by a regioselective esterification of levoglucosan (**1**) with tosyl chloride in pyridine, first presented by Černý and co-workers, in order to afford the 1,6-anhydro-2,4-di-*O*-tosyl-β-ᴅ-glucopyranose as an intermediate. The latter could be used in the next step upon concentration of the reaction mixture under reduced pressure (Scheme 1) [20]. The intermediate was then treated with sodium methoxide in the presence of pyridine in order to generate the Černý epoxide **2** in a 73% yield over two steps [20]. Subjecting the Černý epoxide to sodium azide at an elevated temperature in a mixture of DMF and water afforded the diazide **3** in a 76% yield [21–22]. The presence of the azido groups was supported by a band at ≈2100 cm^−1^ in the FTIR spectrum of the diazide **3**. The 1,6-anydro functionality could then be opened under acidic conditions [22] to give the acetyl pyranoside **4** as a mixture of two anomers (α:β: 1:0.5) in a 95% yield.

**Scheme 2:**
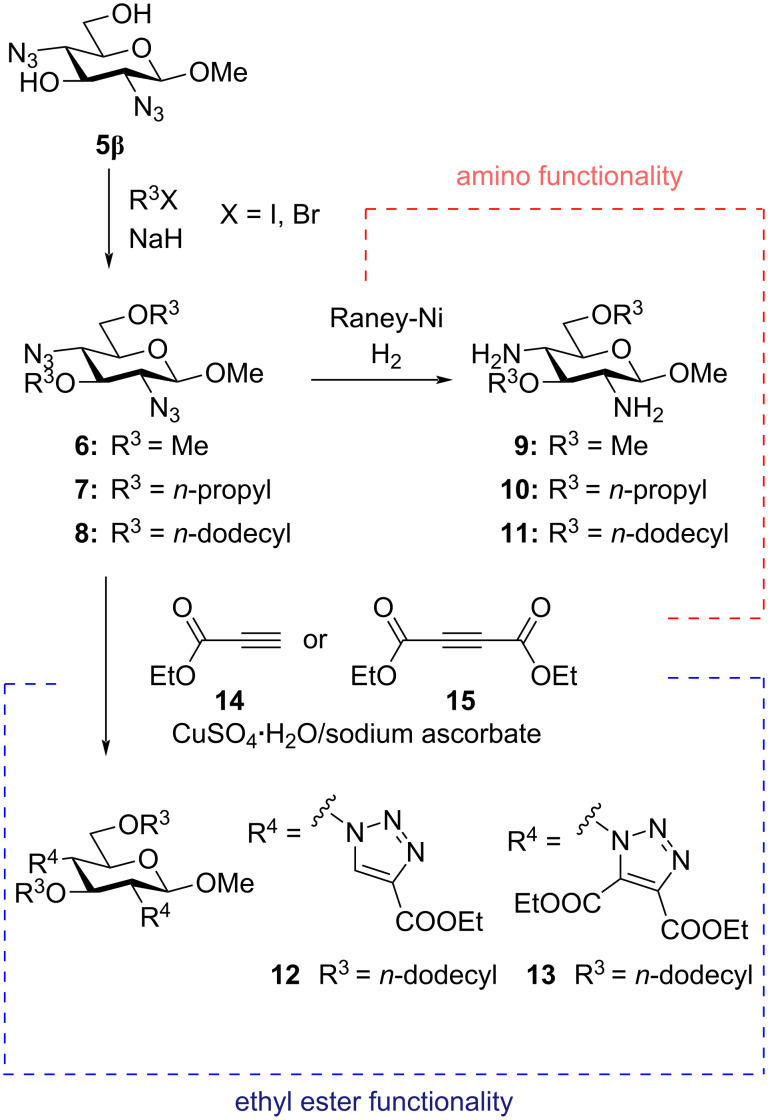
Functionalization of the building block **5β**.

The methyl glucoside **5** was prepared by treating acetyl glucopyranoside **4** with methanol and HCl to generate an anomeric mixture of the methyl glucopyranoside **5**. By recrystallization, it was possible to isolate the β-anomer (**5β**) of the compound **5** as this anomer formed crystals in dichloromethane. Next, the diazide **5β** was treated with different electrophiles (iodomethane, 1-bromopropane or 1-bromododecane) under basic conditions in DMF. By this synthesis, the three new methyl glucosides with *O*-methyl groups (**6**), *O*-*n*-propyl groups (**7**) or *O*-*n*-dodecyl groups (**8**) were synthesized demonstrating that the building block could easily be functionalized with lipophilic tails of different lengths. The azido groups of the three compounds could undergo a reduction by using Raney nickel in order to afford an amino functionality on the 2,4-positions thereby giving diamines **9**, **10**, and **11** (Scheme 2). Having amino groups on these two positions fulfills the requirement of the compound to become a stimuli-responsive surfactant based on the earlier studies by Yuasa and co-workers [11–12]. Furthermore, starting from the azide **8**, it was possible to achieve ester functionalities by a CuAAC reaction [23–24] in the presence of the two different alkynes **14** and **15**, giving rise to the diester derivative **12** and the tetraester derivative **13** (Scheme 2).

**Scheme 3:**
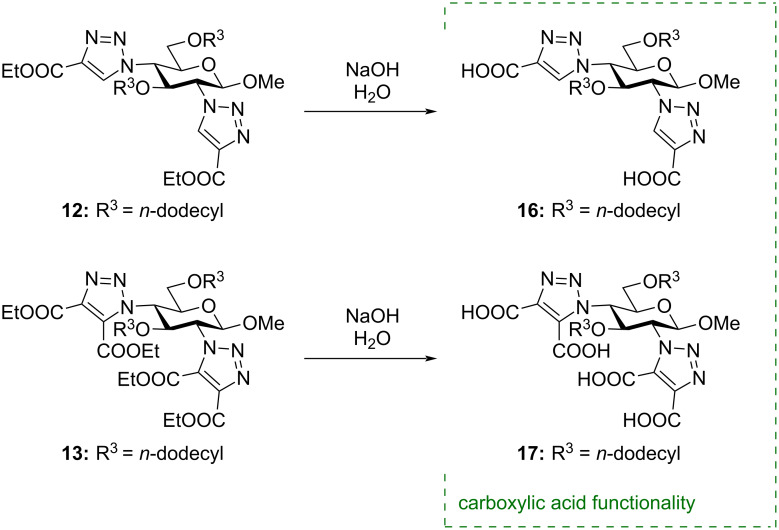
Hydrolysis of the ethyl esters **12** and **13**.

Subsequently, the esters could be hydrolyzed by refluxing the compounds in an aqueous solution of NaOH giving access to the carboxylic acid derivatives **16** and **17** (Scheme 3) demonstrating the versatility of the azido groups to be handles for introducing other metal-chelating groups.

The simplicity of the synthetic steps and the versatility of the products underlines that building block **5** is an ideal template for the synthesis of molecules with a 2,4- and 3,6-functional group pattern. However, after investigating the metal-binding properties of the synthesized diamines and the carboxylic acids, no conformational changes could be observed. Hence, the compounds could not function as stimuli-responsive surfactants in our setup (see Supporting Information File 1 the for binding study with compound **16**). This was somewhat surprising as the prepared amine-functionalized compounds **9**, **10** and **11** resemble the compounds investigated by Yuasa and co-workers as stimuli-responsive lipids [11]. The three compounds reluctance to adopt a ^1^*C*_4_ conformation may be due to a steric clash between the methylene group at C5 and the anomeric substituent when both of these groups are in axial orientation.

**Scheme 4:**
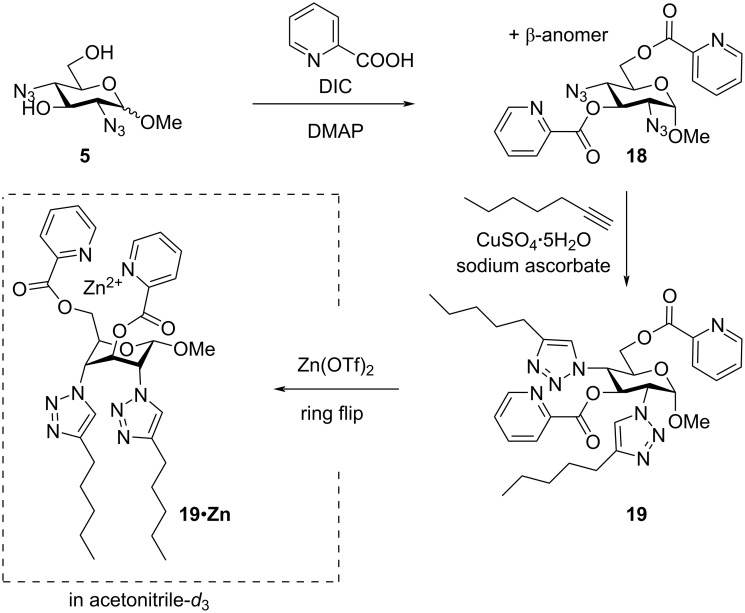
Synthesis of compound **19** from building block **5**.

To further study the scope and versatility of building block **5**, we decided to synthesize a new surfactant from the anomeric mixture of compound **5**. By functionalizing the 3-OH and 6-OH groups with picoloyl groups, using picolinic acid in the presence of *N*,*N'*-diisopropylcarbodiimide (DIC) and 4-dimethylaminopyridine (DMAP), an anomeric mixture of diazide **18** was obtained. The picoloyl group has earlier been used as metal chelator [25]. At this stage, it was possible to separate both anomers of the diazide **18** using flash column chromatography. The pure α-anomer was then subjected to a CuAAC reaction using 1-heptyne and, in only two steps, the new surfactant **19** could be prepared from the common building block **5** (Scheme 4).

### Evaluation of amphiphilic properties

**Figure 2:**
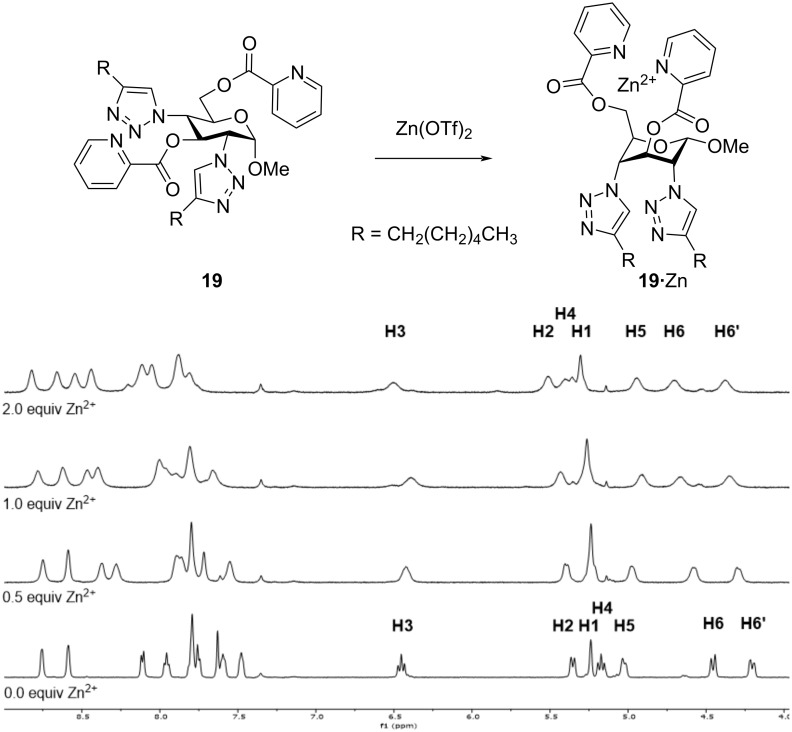
^1^H NMR titration of compound **19** with Zn^2+^ ions in acetonitrile-*d*_3_.

In order to evaluate the conformational change of compound **19** upon the binding of Zn^2+^ ions, a ^1^H NMR titration study was carried out. Thus, a sample of compound **19** was dissolved in acetonitrile-*d*_3_ and a spectrum was recorded. On the basis of the spin–spin coupling constants between the vicinal protons on the pyranoside ring, it was possible to confirm the ^4^*C*_1_ conformation in the absence of Zn^2+^ ions (Figure 2). Upon adding aliquots of Zn(OTf)_2_, as a source of Zn^2+^ ions, the signals in the ^1^H NMR spectrum began to change.

**Figure 3:**
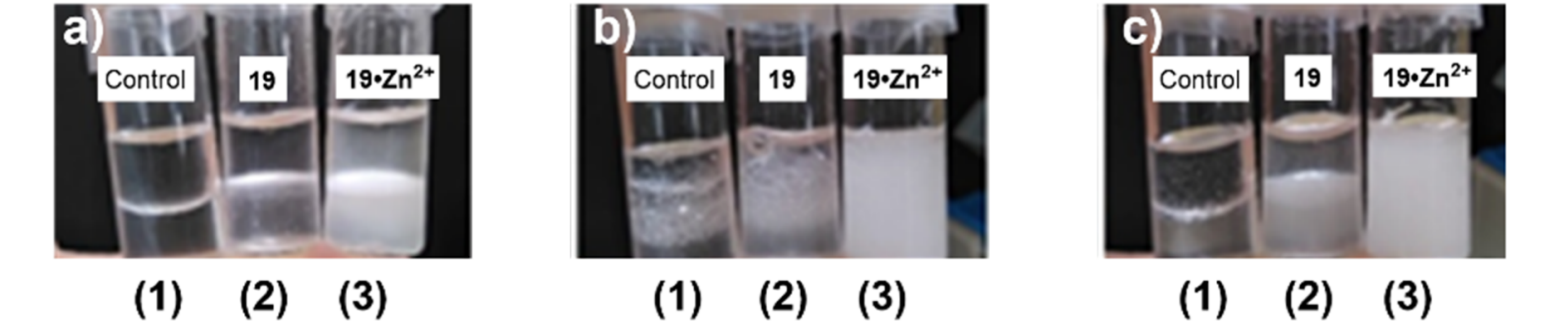
(1) 1:1 Mixture of 1-octanol/H_2_O, (2) same solvent mixture with compound **19**, and (3) same solvent mixture with compound **19** + 1.0 equiv Zn(OTf)_2_ before agitation (a), just after agitation (b), and 50 s after agitation (c).

Already upon the addition of 0.5 equivalents of Zn^2+^ ions, all signals started to broaden indicating a binding in the medium-fast exchange time scale. After the addition of 1.0 equivalent of the metal ions, and even after the addition of an additional equivalent, the broadening was still observed, indicating a binding. However, due to the broadening, no exact conformational information could be deduced from this experiment. The broadening could also have been due to the formation of larger aggregates when binding a metal. If the binding of the metal affords a more polar head group, the molecule becomes amphiphilic and can therefore self-assemble into larger aggregates, resulting in the broadening of the signals in the ^1^H NMR spectrum of compound **19**. As NMR spectroscopy could not give a clear cut insight the surfactant properties, a simple experiment was carried out in order to demonstrate the amphiphilic properties of compound **19** in the absence and in the presence of Zn^2+^ metal ions. A sample of compound **19** was placed between a layer of 1-octanol and H_2_O in two out of three vials. Vial 2 only containing compound **19**, vial 3 containing compound **19** and 1.0 equivalent of Zn(OTf)_2_, and vial 1 being the control, only containing the solvent mixture (Figure 3). All three vials were agitated for a few seconds in order to mix the layers generating an emulsion. The stability of the emulsion is a measure of how well compound **19** acts as an emulsifier. In Figure 3b it can be seen that already after agitation, the control vial (vial 1) and vial 2 show very unstable emulsions while the emulsion formed in vial 3 was stable even after 50 seconds (Figure 3c).

This experiment indicates that compound **19** only works as an emulsifier in the presence of Zn^2+^ ions thereby confirming that the compound displays amphiphilic properties in the presence of the metal ions. The Zn^2+^-induced change in the amphiphilic properties may be due to a ring flip. Unlike compound **9**, **10** and **11**, compound **19** has an α-configuration thereby minimizing the steric clash between the C5 methylene group and the anomeric substituent in a ^1^*C*_4_ conformation as discussed earlier.

## Conclusion

In conclusion, it was possible to convert levoglucosan into building block **5** in only five robust steps. The building block **5** could be purified to afford the two pure anomers using column chromatography. The β-anomer, **5β**, proved to be a very versatile template for synthesizing a variety of compounds using simple and scalable conditions. Under basic conditions, compound **5** could be functionalized with aliphatic chains in a Williamson ether synthesis. Under reductive conditions, the azido groups were converted into amino groups, which are ideal handles for further functionalization. The azido groups also can undergo a CuAAC reaction with alkynes substituted with ester functionalities, which can subsequently be hydrolyzed to either the di- or tetra acids. Using the CuAAC reaction, it was also possible to install aliphatic chains. Introducing picoline residues on the 3,6 positions gave a compound with an on/off amphiphilicity as demonstrated visually. To conclude, the building block **5** gives access to a wide variety of functionalized derivatives and can be used for the synthesis of stimuli-responsive surfactants.

## Supporting Information

File 1 Experimental procedures and spectroscopic data.
