# Supplementary material for: Easy access to a carbohydrate-based template for stimuli-responsive surfactants
Source: Beilstein J Org Chem. 2020 Nov 17;16:2788–94. doi: 10.3762/bjoc.16.229 (PMC7684687; doi:10.3762/bjoc.16.229)

BEILSTEIN JOURNAL OF ORGANIC CHEMISTRY

Supporting Information

for

Easy access to a carbohydrate-based template for stimuli-responsive surfactants

Thomas Holmstrøm, Daniel Raydan and Christian Marcus Pedersen

Beilstein J. Org. Chem. **2020,** *16,* 2788–2794. doi:10.3762/bjoc.16.229

Experimental procedures and spectroscopic data

License and Terms: This is a supporting information file under the terms of the Creative Commons Attribution License (https://creativecommons.org/licenses/by/4.0). Please note that the reuse, redistribution and reproduction in particular requires that the authors and source are credited.
The license is subject to the *Beilstein Journal of Organic Chemistry* terms and conditions: (https://www.beilstein-journals.org/bjoc)

Experimental

General information

Chemicals have been applied directly without further purification prior to use. Dry solvents were collected from an Innovative Technology PS-MD-05 solvent drying system. Air and water-sensitive reactions were carried out under an inert atmosphere of nitrogen. Purification by flash column chromatography was carried out using silica gel (40–63 µm) or aluminum oxide (activated, neutral, Brockmann Activity I). Thin layer chromatography (TLC) was carried out using aluminum sheets coated with silica gel (60F) or plastic sheets coated with aluminum oxide. TLC plates were visualized with UV light before being stained with a 10% solution of H_2SO_4 in ethanol. TLC plates were deactivated by pretreating the plates with a solution of 5% Et_3N in heptane. Concentration on samples under reduced pressure was done at 40 °C. Sodium hydride was used as a 60% dispersion in mineral oil. ^{1}H NMR and $^{13}C\{^{1}H\}$ NMR spectra were recorded on a Bruker 500 MHz Ultra Shield Plus spectrograph equipped with a cryoprobe. ^{1}H NMR spectra were recorded at 500 MHz and ^{13}C NMR spectra were recorded at 126 MHz. Chemical shifts were referenced to the residual signals of the deuterated solvents ($CDCl_3$: ^{1}H: 7.26 ppm, ^{13}C: 77.16 ppm), (DMSO-d_6: ^{1}H: 2.50 ppm, ^{13}C: 39.52 ppm), (acetonitrile-d_3: ^{1}H: 1.94 ppm, ^{13}C: 1.32 ppm) and (MeOD: ^{1}H: 3.34 ppm, ^{13}C: 49.86 ppm). Coupling constants are given in hertz (Hz). High-resolution mass spectrometry (HRMS) was performed on a Bruker SolariX XR 7T ESI/MALDI-FT-ICR-MS instrument using matrix-assisted laser desorption ionization (MALDI) with dithranol as the matrix.

1,6:3,4-Dianhydro-2-*O*-*p*-tolylsulfonyl-β-D-galactopyranose (**2**)

Prepared from a literature procedure [1]. To a cooled (0 °C) solution of levoglucosan (**1**, 6.00 g, 37.0 mmol) in dry pyridine (30 mL) was added a cooled solution of *p*-toluenesulfonyl chloride (15.0 g, 79.0 mmol) in 1:1 pyridine/dichloromethane (60 mL). The reaction mixture was allowed to reach ambient temperature and was stirred for 12 hours. The reaction mixture was quenched with methanol (10 mL) and concentrated in vacuo. The residue was dissolved in dry pyridine (2.4 mL) and a solution of sodium methoxide in methanol (0.6 M, 100 mL) was added dropwise during 3 hours. After 11 hours the reaction concluded with the precipitation of a white solid that corresponded to compound **2**. This compound was isolated by filtration to afford **2** (8.05 g, 27.0 mmol, 73%) as a white solid. HRMS (MALDI/FT-ICR): *m/z*: $[M+Na]^+$ Calc. for $C_{13}H_{14}O_6SNa^+$ 321.0409; Found 321.0402. ^{1}H-NMR ($CDCl_3$, 500 MHz) δ: 7.84 (d, *J* = 8.4 Hz, 2H, arom.), 7.38 (d, *J* = 7.8 Hz, 2H, arom.), 5.17 (s, 1H, H-1), 4.83 (t, *J* = 4.8 Hz, 1H, H-4), 4.40 (s, 1H, H-5), 3.95 (d, *J* = 6.7 Hz, 1H, H-6), 3.61 (t, *J* = 4.8, 4.0 Hz, 1H, H-3), 3.50 (dd, *J* = 6.7, 4.8 Hz, 1H, H-6), 3.14 (dd, *J* = 4.0, 1.6 Hz, 1H, H-2), 2.46 (s, 3H, $-C-CH_3$) ppm. $^{13}C\{^1H\}$-NMR ($CDCl_3$, 126 MHz) δ: 145.8, 133.0, 130.3, 128.1, 98.2, 71.8 (two singlets), 64.9, 53.0, 47.8, 21.8 ppm.

1,6-Anhydro-2,4-diazido-2,4-dideoxy-β-D-glucopyranose (**3**)

Caution: Small molecules containing azides can be potentially explosive substances and should be handled with care. We have not experienced problems working on this scale.

Prepared using a modified procedure [2,3]. To a solution of compound **4** (2.03 g, 6.8 mmol) in DMF/H_2O 5:1 (30 mL) was added sodium azide (2.66 g, 40.9 mmol). The reaction was submitted to 90 °C for 12 hours, followed by TLC (R_f 0.40, toluene/EtOAc 2:1). The solution was allowed to reach ambient temperature and was diluted with EtOAc. Then, the solution was filtered through a pad of silica gel and the filtrate was concentrated in vacuo. The crude product was purified by column chromatography (SiO_2, 2:1 toluene/EtOAc) to afford compound **5** (1.10 g, 5.17 mmol) as a yellow-pale oil in 76% yield. **FT-IR** spectrum of compound **3** shows a band between 2224-2304 cm^{-1}, indicating the presence of azides. ^{1}H-NMR ($CDCl_3$, 500 MHz) δ: 5.52 (s, 1H, H-1), 4.62 (d, *J* = 5.2 Hz, 1H, H- 5), 4.11 (dd, *J* = 7.7, 5.2 Hz, 1H, H-6), 3.87 – 3.82 (m, 1H, H-3), 3.84 – 3.78 (m, 1H, H-6), 3.51 – 3.46 (m, *J* = 3.5 Hz, 1H, H-4), 3.39 (d, *J* = 4.4 Hz, 1H, H-2). $^{13}C\{^1H\}$-NMR (CDCl3, 126 MHz) δ: 100.96, 74.62, 70.91, 67.00, 62.68 (two singlets) ppm.

Acetyl 2,4-diazido-2,4-dideoxy-3,6-di-*O*-acetyl-D-glucopyranoside (**4**)

Caution: Small molecules containing azides can be potentially-explosive substances and should be handled with care. We have not experienced problems working on this scale.

Prepared using a modified procedure [3,4]. A room temperature solution of compound **3** (1.0 g, 4.85 mmol) in acetic anhydride (20 mL) was cooled (0 °C) with an ice bath and trifluoroacetic acid was added (2.1 mL) allowing the reaction to warm up slowly. The reaction was followed by TLC (R_f 0.7, 2:1 toluene/EtOAc), quenched with a saturated solution of $NaHCO_3$ (25 mL) after 12 hours, and concentrated in vacuo. The crude product was purified by flash column chromatography (SiO_2, 4:1 toluene/EtOAc) to afford compound **4** (1.65 g, 4.63 mmol, 95%, as a mixture of α,β isomers) as a yellow-pale oil. ^{1}H-NMR of **4α** has earlier been reported by Paulsen *et al.* In D_2O [4]. ^{1}H-NMR ($CDCl_3$, 500 MHz) δ: 6.29 (d, *J* = 3.6 Hz, 1H, H-1α), 5.52 (d, *J* = 8.5 Hz, 0.5H, H-1β), 5.47 (t, *J* = 10.3 Hz, 1H, H-3α), 5.12 – 5.07 (m, 0.5H, H-3β), 4.36 (dd, *J* = 12.4, 1.5 Hz, 0.5H, H-5β), 4.30 (dd, *J* = 4.3, 3.1 Hz, 2H, H-6α and

H-6α'), 3.84 (dd, *J* = 3.7, 2.5 Hz, 0.5H, H-6β), 3.82 (dd, *J* = 3.7, 2.5 Hz, 0.5H, H-6β), 3.64 (dd, *J* = 10.3, 9.7 Hz, 1H, H-4α), 3.60 – 3.55 (m, 2H, H-5α, H-2β, and H-4β), 3.51 (dd, *J* = 10.3, 3.6 Hz, 1H, H-2α), 2.22 (s, 3H, -C-CH_3α), 2.21 (s, 1.5H, -C-CH_3β), 2.19 (s, 3H, -C-CH_3α), 2.18 (s, 1.5H, -C-CH_3β), 2.11 (s, 3.5H, -C-CH_3α and β) ppm. $^{13}C\{^1H\}$-NMR ($CDCl_3$, 126 MHz) δ: 170.5, 169.8, 169.6, 168.6, 168.5, 92.6, 90.2, 73.2, 73.0, 71.0, 70.3 (one broad singlet), 62.9, 62.6 (two singlets), 60.5, 60.2, 60.0, 21.1, 21.0, 20.8, 20.8, 20.8 ppm.

Methyl 2,4-diazido-2,4-dideoxy-D-glucopyranoside (5 and 5β)

Caution: Small molecules containing azides can be potentially-explosive substances and should be handled with care. We have not experienced problems working on this scale.

Prepared as an inspiration from a literature procedure [3]. A methanolic hydrochloric acid solution was prepared in an ice bath (0 °C) by slowly adding acetyl chloride (4 mL, 56.0 mmol) to dry methanol (9 mL). This methanolic hydrochloric solution was added, at room temperature, to a solution of compound **4** (251 mg, 0.70 mmol) in dry methanol (1 mL). The temperature was increased to 75 °C and the reaction was stirred and followed by TLC (R_f 0.45 and 0.50 toluene/EtOAc) for around 12 hours until completion. The reaction was quenched with a saturated solution of $NaHCO_3$ (20 mL) and filtered. The solution obtained was diluted with EtOAc and washed with brine. The organic phase was dried over $MgSO_4$ and concentrated in vacuo. The product is a mixture of isomers α and β that corresponds to compound **5** (157 mg, 0.64 mmol, 91% yield). The compound was then dissolved in a small amount of dichloromethane and was left at room temperature one complete night to obtain white crystals. By simple filtration and washing with cold dichloromethane, it was possible to obtain the crystals of compound **5β**. HRMS (MALDI/FT-ICR): *m/z*: $[M+Na]^+$ Calc. for $C_7H_{12}N_6O_4Na^+$ 267.08122; Found 267.08127. ^1H-NMR ($CDCl_3$, 500 MHz) of **5** (anomeric mixture) δ: 4.83 (d, *J* = 3.6 Hz, 1H, H-1α), 4.23 (d, *J* = 8.0 Hz, 0.9H, H-1β), 4.08 – 3.99 (m, 1H, H-3α), 3.92 (dd, *J* = 12.3, 2.5 Hz, 1H, H-6α), 3.87 (dd, *J* = 12.2, 1.7 Hz, 1H, H-6α), 3.82 – 3.76 (m, 2H, H-6β), 3.58 (s, 3H, -OCH_3β), 3.57 – 3.53 (m, 3H, H-4α, H-5α, and H-4β), 3.47 (d, *J* = 9.6 Hz, 0.9H, H-3β), 3.42 (s, 3H, -OCH_3α), 3.34 – 3.25 (m, 2H, H-2α, and H-2β), 3.21 (ddd, *J* = 10.0, 4.2, 2.5 Hz, 0.9H, H-5β) ppm. $^{13}C\{^1H\}$-NMR ($CDCl_3$, 126 MHz) of **5** (anomeric mixture) δ: 103.0, 98.7, 74.5, 74.4, 71.3, 70.2, 66.2, 63.4, 62.1, 61.8 (two singlets), 61.1, 57.4, 55.6 ppm. ^1H-NMR (Methanol-d_4, 500 MHz) of **5β** δ: 4.24 (d, *J* = 8.0 Hz, 1H, H-1), 3.83 (dd, *J* = 12.2, 2.1 Hz, 1H, H-6), 3.73 (dd, *J* = 12.2, 4.4 Hz, 1H, H-6), 3.56 (s, 3H, -OCH_3), 3.52 – 3.40 (m, 2H, H-3 and H-5), 3.33 (EtOAc solvent signal), 3.25 – 3.15 (m, 2H, H-2 and H-4) ppm. $^{13}C\{^1H\}$-NMR (Methanol-d_4, 126 MHz) of **5β** δ: 104.0, 76.1, 75.7, 68.3, 63.3, 62.2, 57.3 ppm.

Methyl 2,4-diazido-2,4-dideoxy-3,6-di-*O*-methyl-β-D-glucopyranoside (6)

Caution: Small molecules containing azides can be potentially-explosive substances and should be handled with care. We have not experienced problems working on this scale.

To a cooled (0 °C) solution of compound **5β** (87 mg, 0.36 mmol) in dry DMF (4 mL) was added sodium hydride (200 mg, 8.33 mmol) stirring for 5 minutes before adding methyl iodide (85 µL, 1.37 mmol). The temperature was then increased to room temperature and left for 15 minutes until reaching completion, the reaction was followed by TLC (R_f 0.7, 1:1 heptane/EtOAc). The crude solution was quenched with a saturated solution of

$NaHCO_3$ (5.0 mL), diluted with ethyl acetate and washed with brine. The organic phase was dried over $MgSO_4$ and concentrated in vacuo. Then, a flash column chromatography was carried out (SiO_2, first only heptane was added and then only ethyl acetate) to afford compound **6** (97 mg, 0.36 mmol) as a transparent oil in 100% yield. HRMS (MALDI / FT-ICR): m / z: $[M + Na]^+$ Calc. For $C_9H_{16}N_6O_4Na^+$ 295.1131; Found 295.1201.^{1}H-NMR ($CDCl_3$, 500 MHz) δ : 4.07 (d, J = 7.8 Hz, 1H, H-1), 3.64 (s, 3H, $-OCH_3$), 3.61 – 3.46 (m, 8H, $-OCH_3$, H-6, and H-4), 3.39 (s, 3H, -OCH_3), 3.28 (dd, J = 7.8, 9.5 Hz, 1H, H-2), 3.18 (d, J = 10.0 Hz, 1H, H-5), 3.00 (t, J = 13.8, 9.5 Hz, 1H, H-3) ppm. ^{13}C{^{1}H}-NMR ($CDCl_3$, 126 MHz) δ : 102.9, 83.8, 73.9, 71.2, 65.7, 61.4, 60.9, 59.5, 57.2 ppm.

Methyl 2,4-diazido-2,4-dideoxy-3,6-di-*O*-propyl-β-D-glucopyranoside (7)

Caution: Small molecules containing azides can be potentially-explosive substances and should be handled with care. We have not experienced problems working on this scale.

To a cooled (0 °C) solution of compound **5β** (100 mg, 0.41 mmol) in dry DMF (4.0 mL) was added sodium hydride (160 mg, 6.66 mmol) and stirred for 5 minutes before adding 1-bromopropane (0.2 mL, 2.20 mmol). The temperature was then increased to room temperature and left for 12 hours until reaching completion, followed by TLC (R_f 0.8, 1:1 heptane/EtOAc). The crude solution was quenched with a saturated solution of $NaHCO_3$ (5 mL, diluted with ethyl acetate and washed with brine. The organic phase was dried over $MgSO_4$ and concentrated in vacuo. Then, a flash column chromatography was carried out (SiO_2, first only heptane was added and then 1:1 heptane/EtOAc) to afford compound **7** (113 mg, 0.41 mmol) as a yellow oil in 100% yield. HRMS (MALDI / FT- ICR): m/z: $[M + Na]^+$ Calc. For $C_{13}H_{24}N_6O_4Na^+$ 351.1757; Found 351.1798. ^{1}H-NMR ($CDCl_3$, 500 MHz) δ : 4.06 (d, J = 8.1 Hz, 1H, H-1), 3.79 (m, 1H, $-O-CH_2-C-$), 3.69 (m, 2H, H-6 and $-O-CH_2-C-$), 3.62 (dd, J = 11.0, 4.4 Hz, 1H, H-6), 3.53 (m, 5H, $-O-CH_2-C-$, $-OCH_3$, and H-4), 3.39 (m, 1H, $-O-CH_2-C-$), 3.29 (dd, J = 9.7, 8.1 Hz, 1H, H-2), 3.18 (ddd, J = 10.2, 4.4, 1.9 Hz, 1H, H-5), 3.08 (t, J = 9.6 Hz, 1H, H-3), 1.70 – 1.53 (m, 4H, $-C-CH_2-C-$), 0.96 (t, J = 7.4 Hz, 3H, $-C-CH_3$), 0.91 (t, J = 7.4 Hz, 3H, $-C-CH_3$) ppm. ^{13}C{^{1}H}-NMR ($CDCl_3$, 126 MHz) δ : 103.0, 82.3, 75.4, 74.3, 73.6, 69.6 (broad singlet), 66.1, 62.0, 57.2, 23.4, 10.6, 10.5 ppm.

Methyl 2,4-diazido-2,4-dideoxy-3,6-di-*O*-dodecyl-β-D-glucopyranoside (8)

To a cooled solution (0 °C) of compound **5β** (100 mg, 0.41 mmol) in dry DMF (4 mL) was added sodium hydride (80 mg, 3.33 mmol) and stirred for 5 minutes before adding the 1-bromododecane (0.4 mL, 1.67 mmol). The temperature was then increased to room temperature and left for 12 hours until reaching completion, the reaction was followed by TLC (R_f 0.9, 1:1 heptane/EtOAc). The crude solution was quenched with a saturated solution of $NaHCO_3$ (5 mL), diluted with ethyl acetate and washed with brine. The organic phase was dried over $MgSO_4$ and concentrated in vacuo. Then, a flash column chromatography was carried out (SiO_2, 1:1 heptane/EtOAc) to afford compound **8** (226 mg, 0.39 mmol) as a transparent oil in 95% yield. HRMS (MALDI/FT-ICR): m/z: $[M+Na]^+$ Calc. for $C_{31}H_{60}N_6O_4Na^+$ 603.4568; Found 603.4569. ^{1}H-NMR ($CDCl_3$, 500 MHz) δ : 4.07 (d, J = 8.1 Hz, 1H, H-1), 3.83 (m, 1H, $-O-CH_2-C$), 3.74 (m 1H, $-O-CH_2-C-$), 3.70 (dd, J = 11.0, 1.9 Hz, 1H, H-6), 3.62 (dd, J = 11.0, 4.5 Hz, 1H, H-6), 3.54 (s, 3H, $-OCH_3$), 3.51 (m, 2H, H-4 and $-O-CH_2-C$), 3.47 – 3.37 (m, 1H, $-O-CH_2-C$), 3.29 (dd, J = 9.7, 8.1 Hz, 1H, H-2), 3.18 (ddd, J = 10.3, 4.4, 1.9 Hz, 1H, H-5), 3.08 (t, J = 9.6 Hz, 1H, H-3), 1.69 – 1.52 (m, 4H, $-C-CH_2-C$), 1.26 (broad singlet, $-C-CH_2-C$), 0.88 (t, J = 6.9 Hz, 6H, $-C-CH_3$) ppm. ^{13}C{^{1}H}-NMR ($CDCl_3$, 126 MHz) δ : 103.0, 82.4, 74.3, 73.9, 72.2, 69.7, 66.2, 62.1, 57.3, 32.1, 30.3, 29.7, 26.2, 22.8, 14.2 ppm.

Methyl 2,4-diamino-2,4-dideoxy-3,6-di-*O*-methyl-β-D-glucopyranoside (**9**)

To a solution of compound **6** (83 mg, 0.31 mmol) in dry MeOH (4 mL) together with Raney-Nickel reagent (around 10 mg) was introduced H_2 gas at atmospheric pressure of 1.0 bar for 1 hour. The reaction was followed by TLC (R_f 0.1, 15:1 DCM/MeOH). The crude solution was filtered on celite to get rid of the Raney-Nickel and was concentrated in vacuo to obtain a transparent oil and a white solid together on the bottom of the flask. Adding 2 mL of chloroform, it was possible to dissolve only the oil and filtrate the solid. This solution of chloroform was concentrated again and compound **9** (60 mg 0.27 mmol, transparent oil) was afforded in 90% yield. HRMS (MALDI/FT-ICR): *m/z*: $[M+H]^+$ Calc. for $C_{31}H_{64}N_2O_4H^+$ 221.1496; Found 221.15043. ^{1}H-NMR (Acetonitrile-*d*$_3$, 500 MHz) δ : 3.98 (d, *J* = 7.8 Hz, 1H, H-1), 3.63 (dd, *J* = 10.9, 2.3 Hz, 1H, H-6), 3.49 (m, 4H, H-6 and -OCH_3), 3.42 (s, 3H, -OCH_3), 3.32 (s, 3H, -OCH_3), 3.20 (d, *J* = 2.3 Hz, 1H, H-5), 2.78 (t, *J* = 9.5 Hz, 1H, H-4), 2.59 (d, *J* = 8.8, 9.5 Hz, 1H, H-3), 2.54 (dd, *J* = 7.8, 8.8 Hz, 1H, H-2) ppm. ^{13}C{^{1}H}-NMR (Acetonitrile-*d*$_3$, 126 MHz) δ : 105.3, 86.8, 76.2, 72.8, 59.7, 58.7, 57.3, 56.1, 52.5 ppm.

Methyl 2,4-diamino-2,4-dideoxy-3,6-di-*O*-propyl-β-D-glucopyranoside (**10**)

To a solution of compound **7** (119 mg, 0.43 mmol) in dry MeOH (4 mL) together with Raney-Nickel (around 10 mg) was introduced H_2 gas at atmospheric pressure of 1.0 bar for 2 hours. The reaction was followed by TLC (R_f 0.3, 15:1 DCM/MeOH). The crude solution was filtered on celite to get rid of the Raney-Nickel and was concentrated in vacuo obtaining a transparent oil and a white solid together on the bottom of the flask. Adding 2 mL of chloroform it was possible to dissolve only the oil and filtrate the solid. Concentration in vacuo was performed and compound **10** (114 mg 0.41 mmol, transparent oil) was afforded in 96% yield. HRMS (MALDI/FT-ICR): *m/z*: $[M+H]^+$ Calc. for $C_{31}H_{64}N_2O_4H^+$ 227.2122; Found 277.21264. ^{1}H-NMR (Acetonitrile-*d*$_3$, 500 MHz) δ : 3.98 (d, *J* = 7.8 Hz, 1H, H-1), 3.67 (dd, *J* = 10.9, 2.6 Hz, 1H, H-6), 3.63 (td, *J* = 6.7, 2.2 Hz, 2H, -O-CH_2-C-), 3.54 (dd, *J* = 10.9, 5.4 Hz, 1H, H-6), 3.42 (m, 5H, -OCH_3 and -O-CH_2-C-), 3.20 (ddd, *J* = 9.7, 5.4, 2.7 Hz, 1H, H-5), 2.88 (t, *J* = 9.7 Hz, 1H, H-3), 2.61 (t, *J* = 9.7 Hz, 1H, H-4), 2.55 (dd, *J* = 9.7, 7.8 Hz, 1H, H-2), 1.63 – 1.50 (m, 4H, -C-CH_2-C-), 0.91 (q, *J* = 7.6 Hz, 6H, -C-CH_3) ppm. ^{13}C{^{1}H}-NMR (Acetonitrile-*d*$_3$, 126 MHz) δ : 106.1, 87.0, 77.6, 73.8, 73.6, 71.7, 57.8, 56.9, 54.1, 24.3, 23.6, 10.9 (broad singlet) ppm.

Methyl 2,4-diamino-2,4-dideoxy-3,6-di-*O*-dodecyl-1-β-D-glucopyranoside (**11**)

To a solution of compound **8** (148 mg, 0.25 mmol) in dry MeOH (4 mL) together with Raney-Nickel (around 10 mg) was introduced H_2 gas at atmospheric pressure of 1.0 bar for 3 hours. The reaction was followed by TLC (R_f 0.5, 15:1 DCM/MeOH). The crude solution was filtered on celite to get rid of the Raney-Nickel and was concentrated in vacuo affording a transparent oil and a white solid together on the bottom of the flask. Adding 2 mL of chloroform it was possible to dissolve only the oil and filtrate the solid. Concentration in vacuo was performed and compound **11** (129 mg 0.24 mmol, transparent oil) was afforded in 96% yield. HRMS (MALDI/FT-ICR): *m/z*: $[M+H]^+$ Calc. for $C_{31}H_{64}N_2O_4H^+$ 529.4939; Found 529.49375. ^{1}H-NMR (Acetonitrile-*d*$_3$, 500 MHz) δ : 3.96 (d, *J* = 7.8 Hz, 1H, H-1), 3.65 (m, 3H, -O-CH_2-C- and H-6), 3.53 (dd, *J* = 10.9, 5.3 Hz, 1H, H-6), 3.44 (m, 2H, -O-CH_2-C-), 3.41 (s, 3H, -OCH_3), 3.18 (ddd, *J* = 9.6, 5.3, 2.6 Hz, 1H, H-5), 2.85 (t, *J* = 9.6 Hz, 1H, H-3), 2.61 (t, *J* = 9.6 Hz, 1H, H-4), 2.54 (dd, *J* = 9.6, 7.8 Hz, 1H, H-2), 1.54 (tt, *J* = 13.9, 6.5 Hz, 4H, -C-CH_2-C-), 1.28 (broad singlet, -C-CH_2-C-), 0.88 (t, *J* = 6.9 Hz, 6H, -C-CH_3) ppm. ^{13}C{^{1}H}-NMR (Acetonitrile-*d*$_3$, 126 MHz) δ : 105.9, 86.0, 76.7, 71.1, 70.8, 56.6, 55.8, 53.0, 31.7, 29.3, 29.1 (m), 25.7, 22.2, 13.3 ppm.

Compound 12

Compound **8** (225 mg, 0.39 mmol) was dissolved in a solvent mixture of 1:1 THF/H_2O (3.7 mL) at room temperature, together with ethyl propiolate **14** (150 µL, 1.35 mmol). The system was refluxed and aqueous solutions of $CuSO_4·5H_2O$ (19 mg, 0.077 mmol) and sodium ascorbate (130 mg, 0.15 mmol) were introduced avoiding the entrance of oxygen in the flask. A yellow color immediately appeared, and the reaction was stirred until the color disappeared (around 3 hours). The reaction was stopped after observing complete disappearance of the starting material by TLC (R_f 0.4, 2:1 heptane/EtOAc). The solution was diluted with ethyl acetate and the organic phase was washed with brine, dried over $MgSO_4$, concentrated in vacuo, and subjected to flash column chromatography (SiO_2, 2:1 heptane/EtOAc) to afford compound **12** (223 mg, 0.29 mmol) as a grey wax, in 74% yield. HRMS (MALDI/FT-ICR): *m/z:* $[M+H]^+$ Calc. for $C_{41}H_{72}N_6O_8\,H^+$ 777.5464; Found 777.5490. ^{1}H-NMR ($CDCl_3$, 500 MHz) δ : 8.13 (s, 1H, arom.), 8.12 (s, 1H, arom.), 5.06 (d, *J* = 8.2 Hz, 1H, H-1), 4.98 (t, *J* = 10.1 Hz, 1H, H-3), 4.70 (t, *J* = 10.1 Hz, 1H, H-4), 4.49 – 4.37 (m, 3H, -CO_2CH_2-C and H-5), 4.22 (dd, *J* = 10.1, 8.2 Hz, 1H, H-2), 3.61 (dd, *J* = 11.4, 1.7 Hz, 1H, H-6), 3.50 – 3.43 (m, 1H, -OCH_2-C-), 3.42 (s, 3H, -OCH_3), 3.23 (dt, *J* = 9.5, 6.9 Hz, 1H, -OCH_2-C-), 3.11 (dd, *J* = 11.4, 3.1 Hz, 1H, H-6), 2.69 (m, *J* = 9.2 Hz, 1H, -O-CH_2-C-), 2.64 – 2.56 (m, 1H, -OCH_2-C), 1.42 (td, *J* = 7.1, 1.1 Hz, 6H, -CO2-C-CH3), 1.27 (broad singlet, -C-CH_2-C-), 1.10 – 1.00 (m, 2H, -C-CH_2-C-), 0.93 (m, 2H, -C-CH_2-C-), 0.88 (td, *J* = 6.9, 5.2 Hz, -C-CH_3), 0.74 (s, 2H, -C-CH_2-C-) ppm. $^{13}C\{^1H\}$-NMR ($CDCl_3$, 126 MHz) δ : 160.7, 160.5, 130.3 (two singlets), 129.9, 101.8, 79.6, 73.9, 73.7, 72.3, 68.2, 67.3, 62.4, 61.6 (two singlets), 57.8, 32.1, 30.3 – 28.4 (m), 26.2, 25.7, 22.8, 14.4, 14.4, 14.2 ppm.

Compound 13

Compound **8** (237 mg, 0.41 mmol) was dissolved in dry DMF (3 mL) together with an excess of diethyl acetylenedicarboxylate **15** (0.23 mL). The solution was refluxed and the reaction was followed by TLC (R_f 0.65, 1:1 heptane/EtOAc). After 2 hours (longer periods of time show side products in the TLC) the reflux was stopped and the solution was diluted with ethyl acetate. The crude solution was washed with brine and the organic phase was dried over Mg_2SO_4 and concentrated in vacuo. Flash column chromatography (SiO_2, heptane/EtOAc 10:1 - 4:1 - and pure EtOAc) to afford compound **13** (256 mg, 0.28 mmol) as a yellow-pale oil in 68% yield. HRMS (MALDI/FT-ICR): *m/z*: $[M+H]^+$ Calc. for $C_{47}H_{80}N_6O_{12}\,H^+$ 921.5907; Found 921.5916. The compound was directly used for the synthesis of **17** without further analysis. The characterization could not be made with NMR due to low solubility.

Compound 16

A solution of compound **12** (200 mg, 0.26 mmol) in a 0.6 mM solution of NaOH (180 mg of NaOH dissolved in 8.5 mL of water) was refluxed for 48 hours. The reaction was followed by TLC (R_f 0.8, 5:1 DCM/MeOH), the reflux was stopped and the solution was neutralized with a 1.0 M solution of HCl until reaching a pH lower than 6.0.

Then, the solution was diluted with ethyl acetate. The organic phase was dried over Mg_2SO_4 and concentrated in vacuo to afford compound **16** (121 mg, 0.17 mmol) as a white solid in 65% yield. HRMS (MALDI/FT-ICR): *m/z*: $[M+H]^+$ Calc. for $C_{37}H_{64}N_6O_8$ H^+ 721.4858; Found 721.48382. ^{1}H-NMR (Acetonitrile-*d*$_3$, 500 MHz) δ : 8.42 (s, 1H, arom.), 8.40 (s, 1H, arom.), 5.12 (d, *J* = 8.4 Hz, 1H, H-1), 4.82 – 4.68 (two triplets, 2H, H-4 and H-3 respectively), 4.47 (dd, *J* = 9.8, 8.4 Hz, 1H, H-2), 4.40 (dd, *J* = 9.8, 1.3 Hz, 1H, H-5), 3.50 (dd, *J* = 11.4, 2.4 Hz, 1H, H-6), 3.38 (s, 3H, $-OCH_3$), 3.38 – 3.32 (m, 2H, $-O-CH_2-C-$), 3.22 (m, 2H, $-O-CH_2-C-$), 3.16 (dd, *J* = 11.4, 3.7 Hz, 1H, H-6), 2.57 (m, 2H, $-C-CH_2-C-$), 1.45 (m, 4H), 1.28 (broad singlet, $-C-CH_2-C-$), 1.19 – 1.09 (m, 4H, $-C-CH_2-C-$), 1.09 – 1.00 (m, 4H, $-C-CH_2-C-$), 0.88 (t, *J* = 6.9 Hz, 6H, $-C-CH_3$), 0.71 (m, 4H, $-C-CH_2-C-$) ppm. $^{13}C\{^1H\}$-NMR (Acetonitrile-*d*$_3$, 126 MHz) δ : 161.5, 161.4, 140.3, 140.2, 130.8, 130.6, 102.1, 80.9, 74.2, 73.7, 72.2, 69.4, 67.3, 63.4, 57.6, 32.6, 30.3 (m), 26.8, 26.1, 23.3, 14.3 ppm.

Compound 17

A solution of compound **13** (254 mg, 0.28 mmol) in a 0.6 mM solution of NaOH (180 mg of NaOH dissolved in 8.5 mL of water) was refluxed. The reaction was followed by TLC (R_f 0.7, 3:1 DCM/MeOH), after 48 hours the reflux was stopped and the solution was neutralized with a 1.0 M solution of HCl until reaching a pH lower than 6.0. The solution was diluted with ethyl acetate and the organic phase was dried over Mg_2SO_4 and concentrated *in vacuo* to afford compound **17** (138 mg, 0.17 mmol) as a yellow oil in 62% yield. HRMS (MALDI/FT-ICR): *m/z*: $[M+Na]^+$ Calc. for $C_{39}H_{64}N_6O_{12}$ Na^+ 831.4474; Found 831.44829. ^{1}H-NMR (Acetonitrile-*d*$_3$, 500 MHz) δ : 5.98 (t, *J* = 9.5 Hz, 1H, H-4), 5.79 (dd, *J* = 9.5, 8.7 Hz, 1H, H-2), 5.22 (d, *J* = 8.7 Hz, 1H, H-1), 4.90 (t, *J* = 9.5 Hz, 1H, H-3), 4.58– 4.46 (m, 1H, H-5), 3.51 (dd, *J* = 11.1, 4.1 Hz, 1H, H-6), 3.36 (s, 3H, $-OCH_3$), 3.31 (dd, *J* = 11.1, 4.2 Hz, 1H, H-6), 3.26 (m, 1H, $-O-CH_2-C-$), 3.20 (m, 1H, $-O-CH_2-C-$), 1.28 (broad singlet, $-C-CH_2-C-$), 1.15 (m, 1H, $-C-CH_2-C-$), 1.03 (m, 1H, $-C-CH_2-C-$), 0.93-0.82 (m,7H, $-C-CH_3$), 0.68 (m, 2H, $-C-CH_2-C-$) ppm. $^{13}C\{^1H\}$-NMR (Acetonitrile-*d*$_3$, 126 MHz) δ : 101.5, 73.3, 72.4, 71.0, 69.0, 61.5, 56.4, 31.3, 29.0 (m), 25.3, 25.0, 22.1, 13.0 ppm.

Compound 18

To a solution of compound **5** (1:0.9 α:β, 382 mg, 1.56 mmol) in dichloromethane (12 mL) together with picolinic acid (480 mg, 3.90 mmol), were added *N*,*N*'-diisopropylcarbodiimide (550 µL, 3.23 mmol), and 4-dimethylaminopyridine (19 mg, 0.16 mmol), at room temperature. The reaction was followed by TLC (R_f 0.3 and 0.4, 5:1 EtOAc/heptane) and stirred for 12 hours until completion. The crude solution was diluted with ethyl acetate and cleaned with brine. The organic phase was dried over Mg_2SO_4 and concentrated in vacuo. Flash column chromatography was performed (SiO_2, EtOAc/heptane, 1:1–5:1) to separate both isomers, **β-anomer** (308 mg, 0.68 mmol, 44% yield) and **α-anomer (18)** (340 mg ,0.75 mmol, 48% yield), as pure transparent oils. **β- anomer:** ^{1}H-NMR ($CDCl_3$, 500 MHz) δ : 8.84 – 8.77 (m, 2H, arom.), 8.26 (d, *J* = 1.6 Hz, 1H, arom.), 8.13 (d, *J* = 7.8 Hz, 1H, arom.), 7.89 (m, 2H, arom.), 7.68 – 7.45 (m, 2H, arom.), 5.34 (t, *J* = 10.2 Hz, 1H, H-3), 4.75 (d, *J* = 12.2 Hz, 1H, H-6), 4.66 (dd, *J* = 12.2, 4.6 Hz, 1H, H-6), 4.40 (d, *J* = 7.9 Hz, 1H, H-1), 4.01 (t, *J* = 10.2 Hz, 1H, H-4), 3.76 – 3.65 (m, 2H, H-2 and H-5), 3.57 (s, 3H, $-OCH_3$) ppm. ^{1}H-NMR (Acetonitrile-*d*$_3$, 500 MHz) δ : 8.76 (t, *J* = 5.2 Hz, 2H, arom.), 8.20 (d, *J* = 7.9 Hz, 1H, arom.), 8.14 (d, *J* = 7.8 Hz, 1H, arom.), 7.96 (dtd, *J* = 11.4, 7.7, 1.9 Hz, 2H, arom.) 7.60 (ddd, *J* = 12.1, 7.6, 4.7 Hz, 2H, arom.), 5.36 (t, *J* = 9.8 Hz, 1H, H-3), 4.70 – 4.63 (m, 2H, H-6), 4.54 (d, *J* = 8.2 Hz, 1H, H-1), 3.93 (t, *J* = 9.8 Hz, 1H, H-4), 3.83 (ddd, *J* = 9.8, 4.5, 2.0 Hz, 1H, H-5), 3.71 (dd, *J* = 9.8,

8.2 Hz, 1H, H-2), 3.53 (s, 3H, -OCH_3) ppm. $^{13}C\{^1H\}$-NMR ($CDCl_3$, 126 MHz) δ : 150.21 (broad singlet), 137.4, 137.2, 127.8, 127.3, 126.3, 125.5, 103.2, 74.2, 72.4, 63.9, 63.7, 60.2, 57.6 ppm. **α-anomer**: ^1H-NMR ($CDCl_3$, 500 MHz) δ : 8.80 (s, 2H, arom.), 8.25 (d, *J* = 7.8 Hz, 1H, arom.), 8.14 (d, *J* = 7.8 Hz, 1H, arom.), 7.93 – 7.84 (m, 2H, arom.), 7.53 (s, 2H, arom.), 5.84 (t, *J* = 9.3 Hz, 1H, H-3), 4.97 (broad s, 1H, H-1), 4.71 (broad s, 2H, H-6), 4.02 (broad s, 2H, H-4 and H- 5), 3.50 (broad s, 4H, H-2 and -OCH_3) ppm. ^1H-NMR (Acetonitrile-d_3, 500 MHz) δ : 8.79 – 8.68 (m, 2H, arom.), 8.20 (d, *J* = 7.8 Hz, 1H, arom.), 8.14 (d, *J* = 7.8 Hz, 1H, arom.), 7.96 (dtd, *J* = 12.6, 7.7, 1.8 Hz, 2H, arom.), 7.60 (dddd, *J* = 13.8, 7.7, 4.7, 1.3 Hz, 2H, arom.), 5.64 (dd, *J* = 10.6, 9.3 Hz, 1H, H-3), 5.01 (d, *J* = 3.4 Hz, 1H, H-1), 4.77 – 4.45 (m, 2H, H-6), 4.23 – 3.80 (m, 2H, H-4 and H-5), 3.64 (dd, *J* = 10.6, 3.4 Hz, 1H, H-2), 3.46 (s, 3H, -OCH_3) ppm. $^{13}C\{^1H\}$-NMR ($CDCl_3$, 126 MHz) δ : 150.1, 150.0, 137.3, 137.1, 127.6, 127.2, 126.1, 125.3, 99.1, 72.3, 68.1, 63.7, 60.9, 60.0, 55.7 ppm.

Compound 19

To a solution of compound **18** (56 mg, 0.12 mmol) in a solvent mixture of 1:1 THF/H_2O (1 mL) at room temperature together with 1-heptyne (40 µL, 0.30 mmol), were added aqueous solutions of $CuSO_4 \cdot 5H_2O$ (3 mg, 0.01 mmol) and sodium ascorbate (5 mg, 0.02 mmol) avoiding the entrance of oxygen in the flask. A yellow color immediately appeared, and the reaction was stirred for 12 hours with a slow disappearance of the yellow color from the solution. The reaction was followed by TLC (R_f 0.4, EtOAc). The crude solution was diluted with ethyl acetate and cleaned with water. The organic phase was dried over Mg_2SO_4 and concentrated in vacuo. Flash column chromatography was performed (SiO_2, EtOAc to 10:1 EtOAc/heptane) to afford compound **19** (58 mg, 0.09 mmol) as a white solid in 73% yield. HRMS (MALDI/FT-ICR): *m/z*: $[M+H]^+$ Calc. for $C_{33}H_{43}N_8O_6$ H^+ 647.3300; Found 647.33074. ^1H-NMR (Acetonitrile-d_3, 500 MHz) δ: 8.69 (d, *J* = 4.7 Hz, 1H, arom.), 8.52 (d, *J* = 4.7 Hz, 1H, arom.), 8.04 (d, *J* = 7.8 Hz, 1H, arom.), 7.88 (td, *J* = 7.7, 1.6 Hz, 1H, arom.), 7.78 – 7.64 (m, 3H, arom.), 7.57 (s, 1H, arom.), 7.52 (dd, *J* = 8.0, 4.6 Hz, 1H, arom.), 7.44 – 7.38 (m, 1H, arom.), 6.39 (t, *J* = 10.5 Hz, 1H, H-3), 5.30 (dd, *J* = 11.1, 3.4 Hz, 1H, H-2), 5.18 (d, *J* = 3.3 Hz, 1H, H-1), 5.12 (t, *J* = 10.3 Hz, 1H, H-4), 4.97 (dt, *J* = 10.7, 3.3 Hz, 1H, H-5), 4.39 (dd, *J* = 12.5, 2.5 Hz, 1H, H-6), 4.15 (dd, *J* = 12.5, 4.4 Hz, 1H, H-6), 3.45 (s, 3H, -OCH_3), 2.51 (t, *J* = 7.5 Hz, 2H, -C- CH_2-C-), 2.43 (t, *J* = 7.5 Hz, 2H, -C-CH_2-C-), 1.52 – 1.40 (m, 2H, -C-CH_2-C-), 1.31 (m, 2H, -C-CH_2-C-), 1.09 (m, 8H, -C- CH_2-C-), 0.73 (t, *J* = 7.2 Hz, 3H, -C-CH_3), 0.67 (t, *J* = 7.3 Hz, 3H, -C-CH_3) ppm. $^{13}C\{^1H\}$-NMR (Acetonitrile-d_3, 126 MHz) δ: 150.9, 150.8, 138.2, 138.1, 128.5, 126.1, 123.3, 122.0, 99.2, 71.5, 68.8, 63.9, 63.1, 61.1, 56.3, 31.6, 31.5, 29.7, 29.5, 25.9, 25.8, 22.9, 22.9, 14.2, 14.1 ppm.

Binding study with compound **16**

^{1}H-NMR (500 MHz) spectrum of compound **16** in DMSO-d_6 in the presence of Zn^{2+} and Ca^{2+} ions and in the presence of NaOD together with Zn^{2+} in order to promote binding. No binding is observed

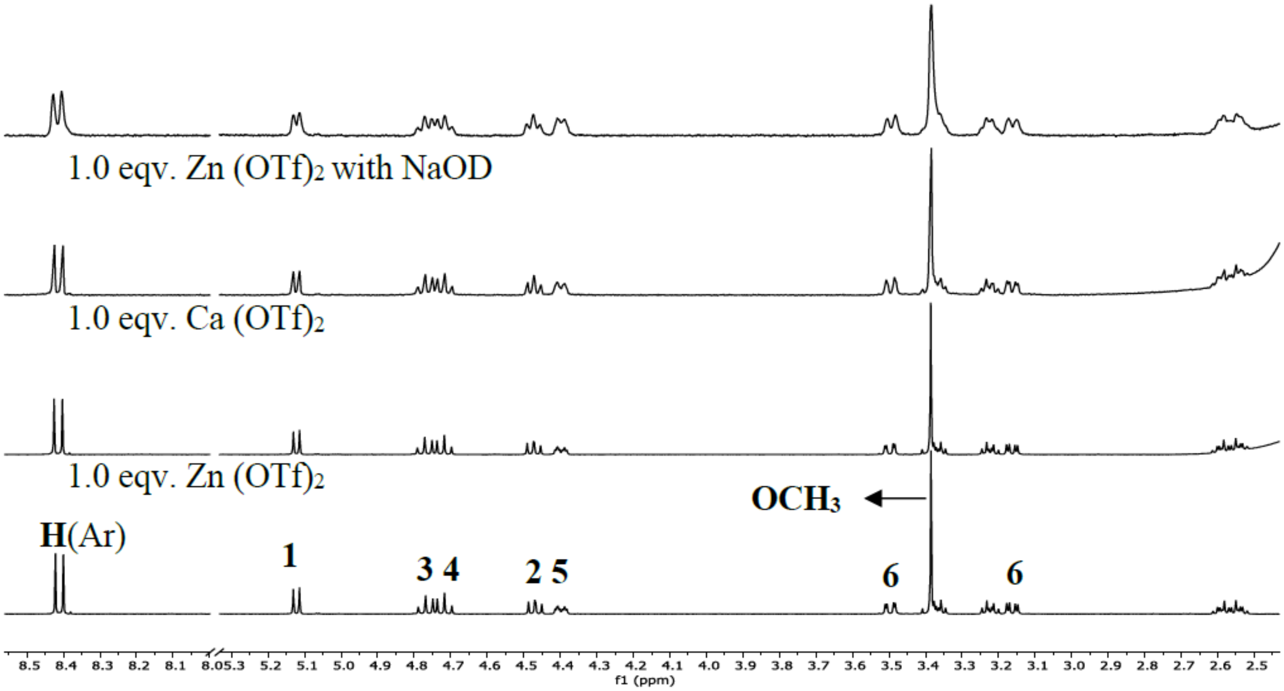

References

(1) Černý, M.; Gut, V.; Pacák, J. *Collect. Czech. Chem. Commun.* **1961**, *26* (10), 2542–2550

(2) Bailliez, V.; Olesker, A.; Cleophax, J. *Tetrahedron* **2004**, *60* (5), 1079–1085. doi:https://doi.org/10.1016/j.tet.2003.11.080

(3) Kato, T.; Vasella, A.; Crich, D. *Carbohydr. Res.* **2017**, *448*, 10–17. doi:https://doi.org/10.1016/j.carres.2017.05.015

(4) Paulsen, H.; Koebernick, H. *Chem. Ber.* **1976**, *109* (1), 104–111. doi:10.1002/cber.19761090108

NMR spectra

^{1}H-NMR (500 MHz) spectrum of compound **2** in chloroform-*d*.

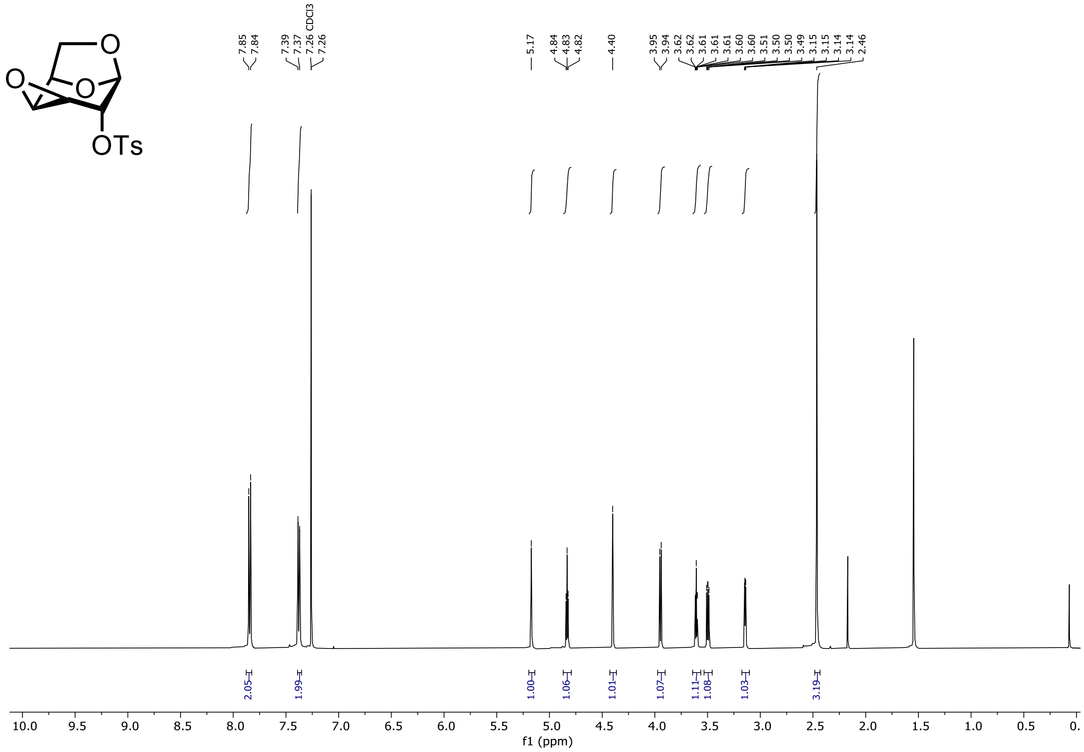

^{13}C{^{1}H}-NMR (126 MHz) spectrum of compound **2** in chloroform-*d*.

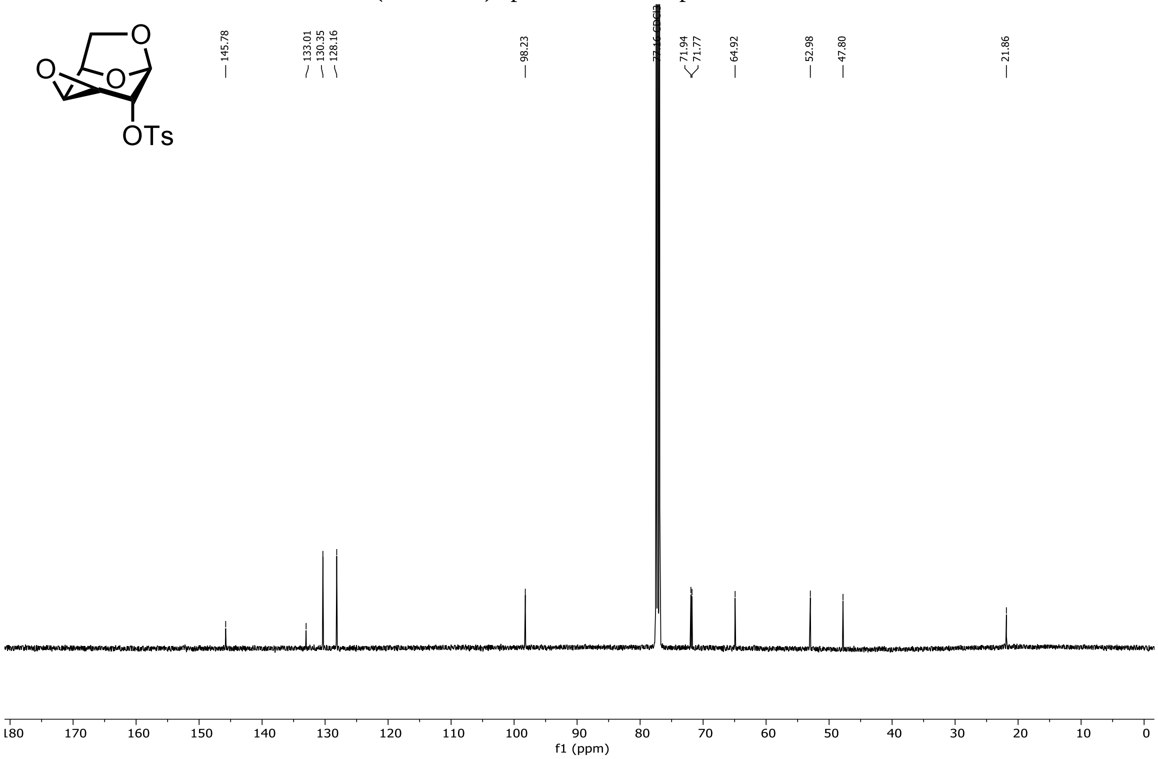

COSY spectrum of compound **2** in chloroform-*d*.

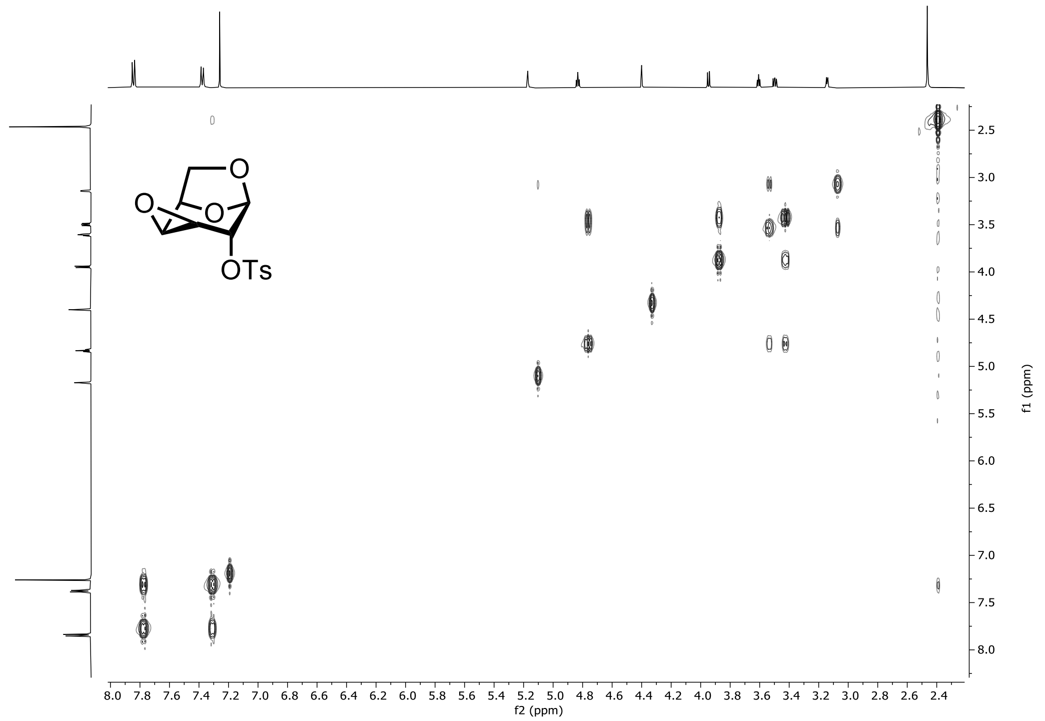

HSQC spectrum of compound **2** in chloroform-*d*.

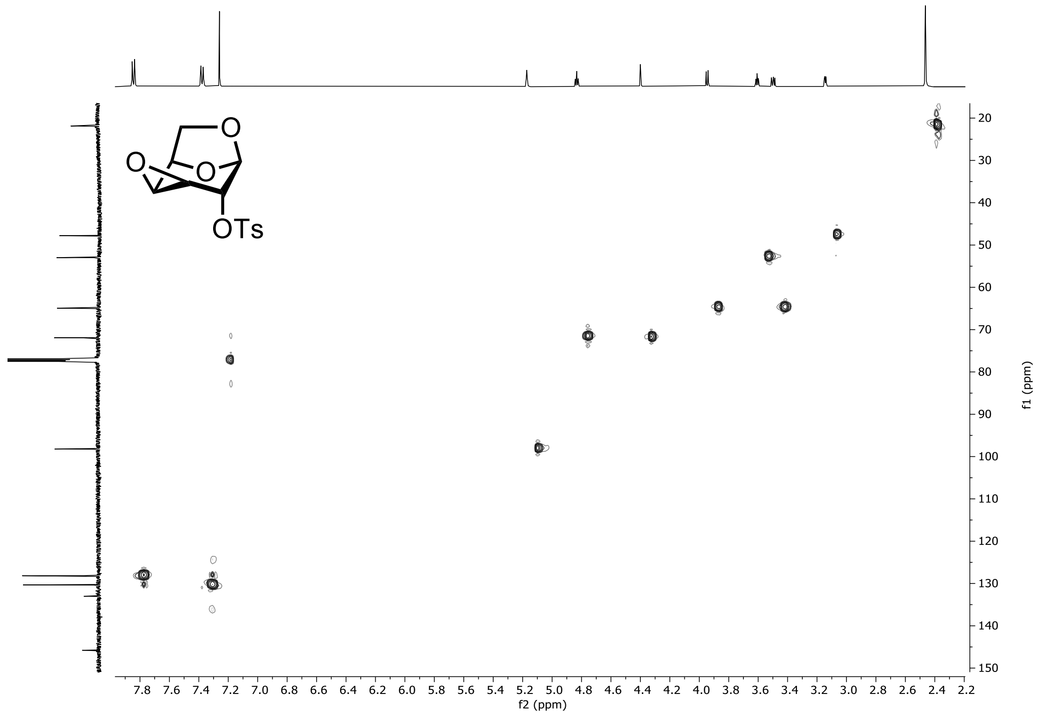

^{1}H-NMR (500 MHz) spectrum of compound **3** in chloroform-*d*.

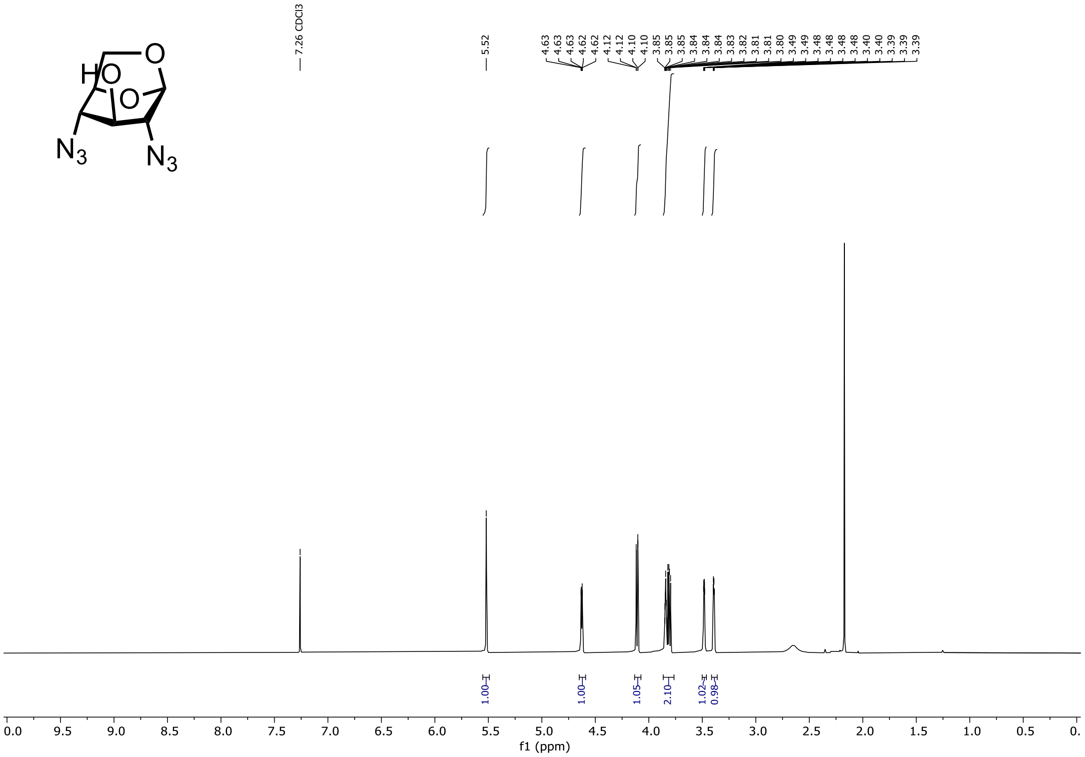

^{13}C{^{1}H}-NMR (126 MHz) spectrum of compound **3** in chloroform-*d*.

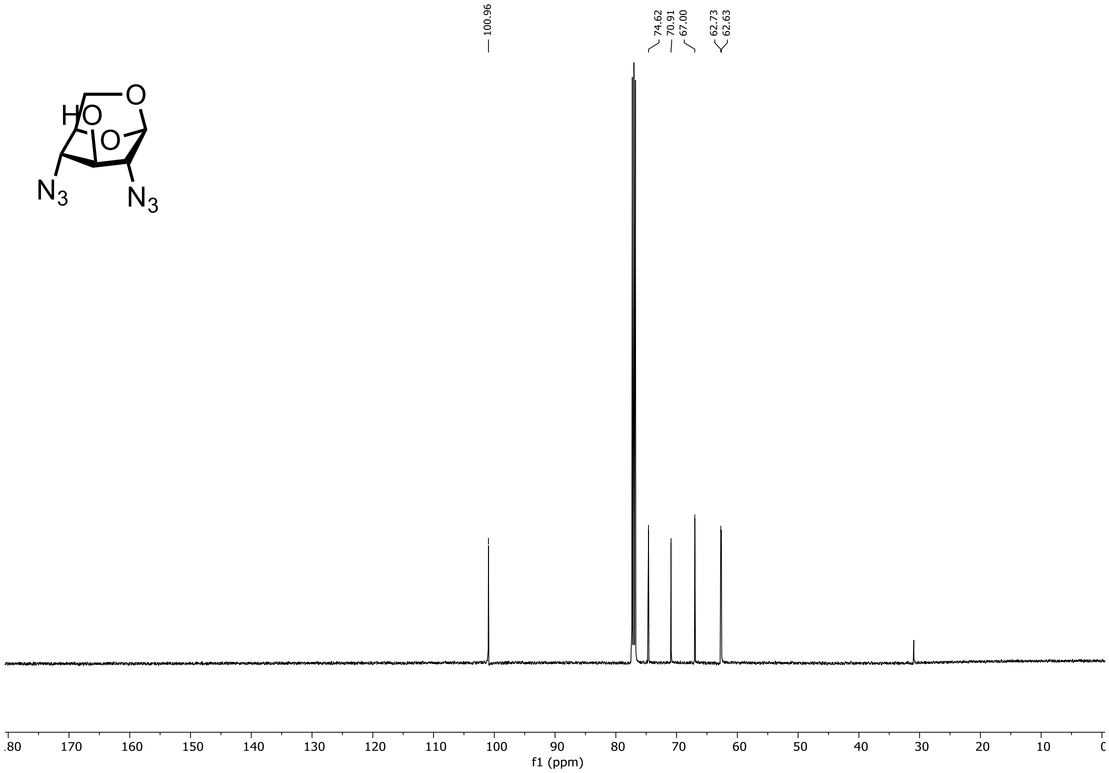

COSY spectrum of compound **3** in chloroform-*d*.

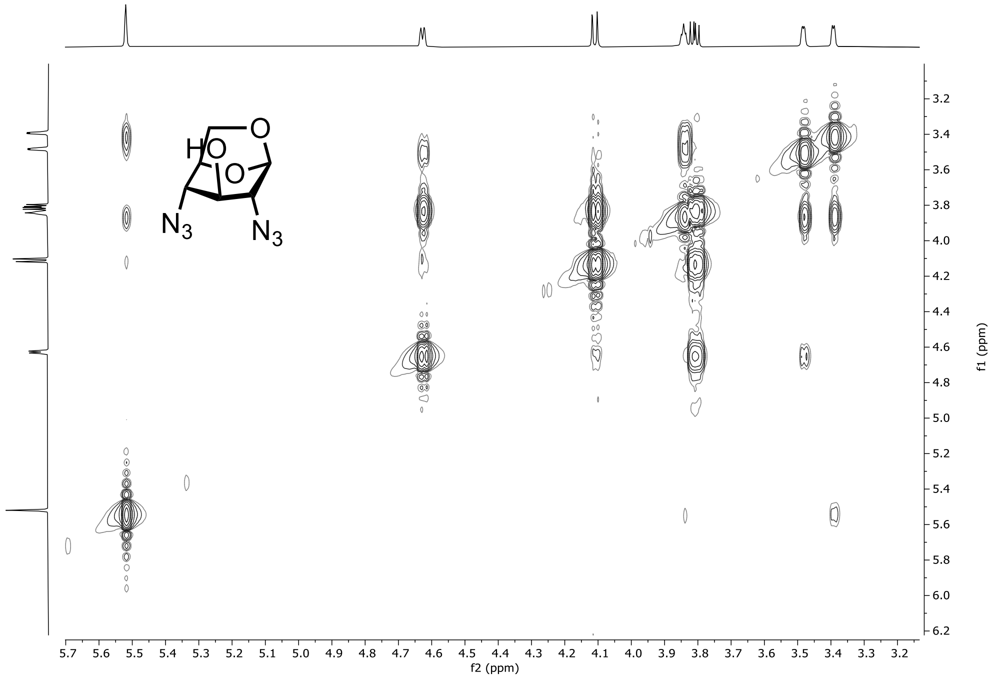

HSQC spectrum of compound **3** in chloroform-*d*.

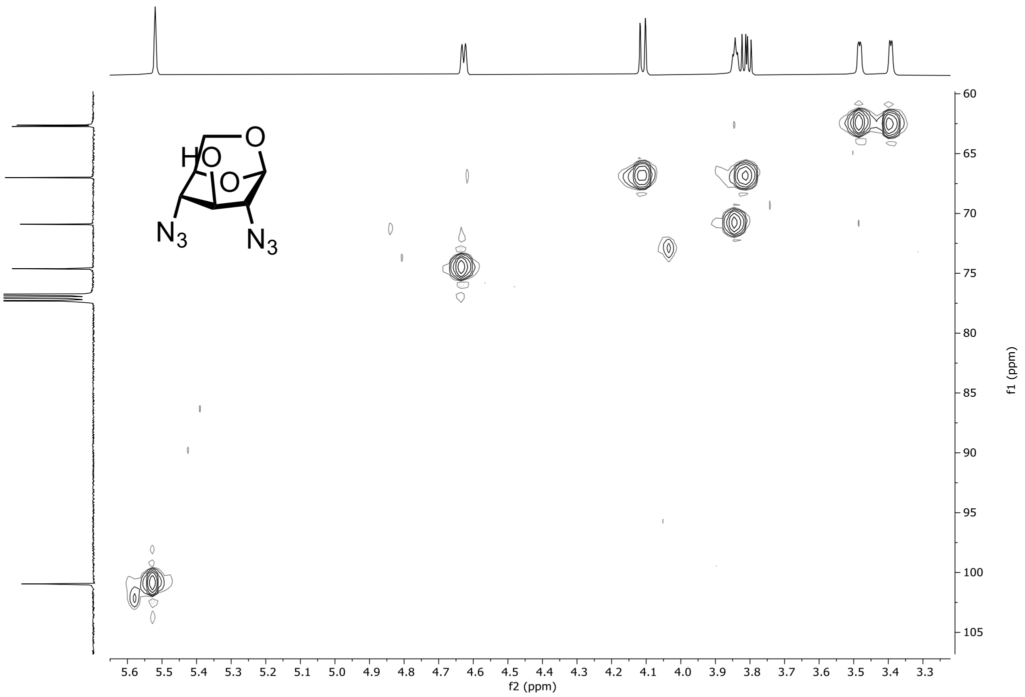

^{1}H-NMR (500 MHz) spectrum of compound **4** in chloroform-*d*.

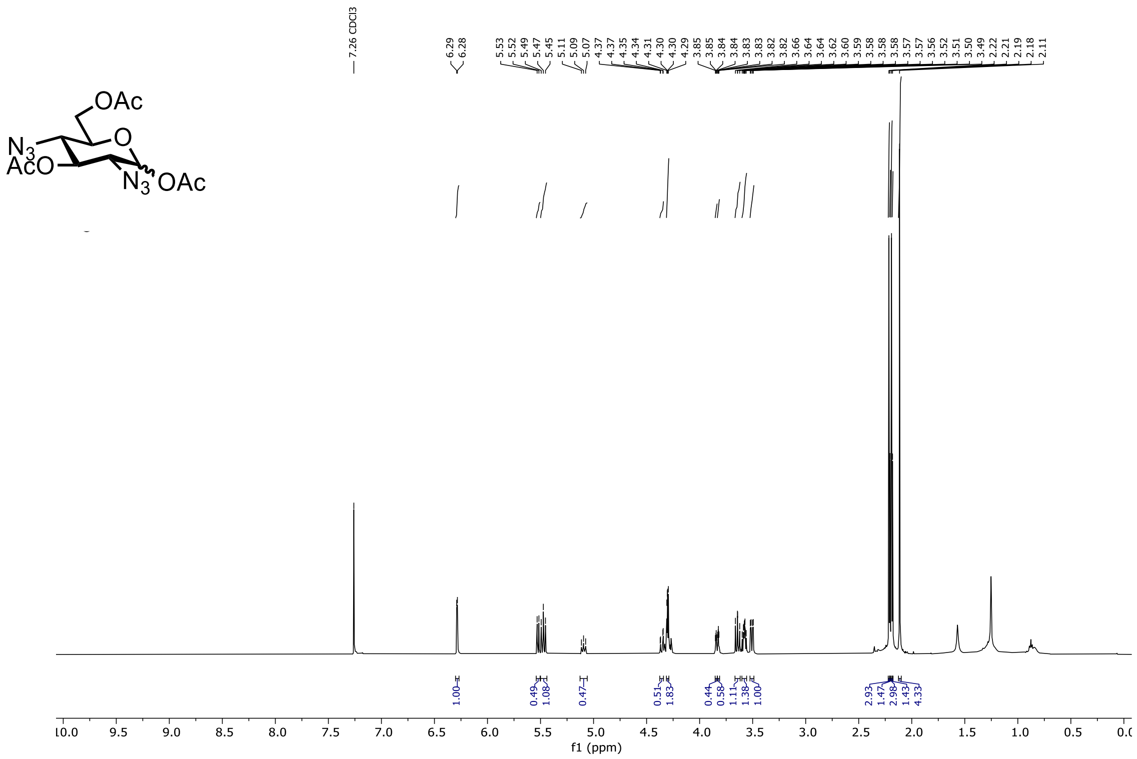

^{13}C{^{1}H}-NMR (126 MHz) spectrum of compound **4** in chloroform-*d*.

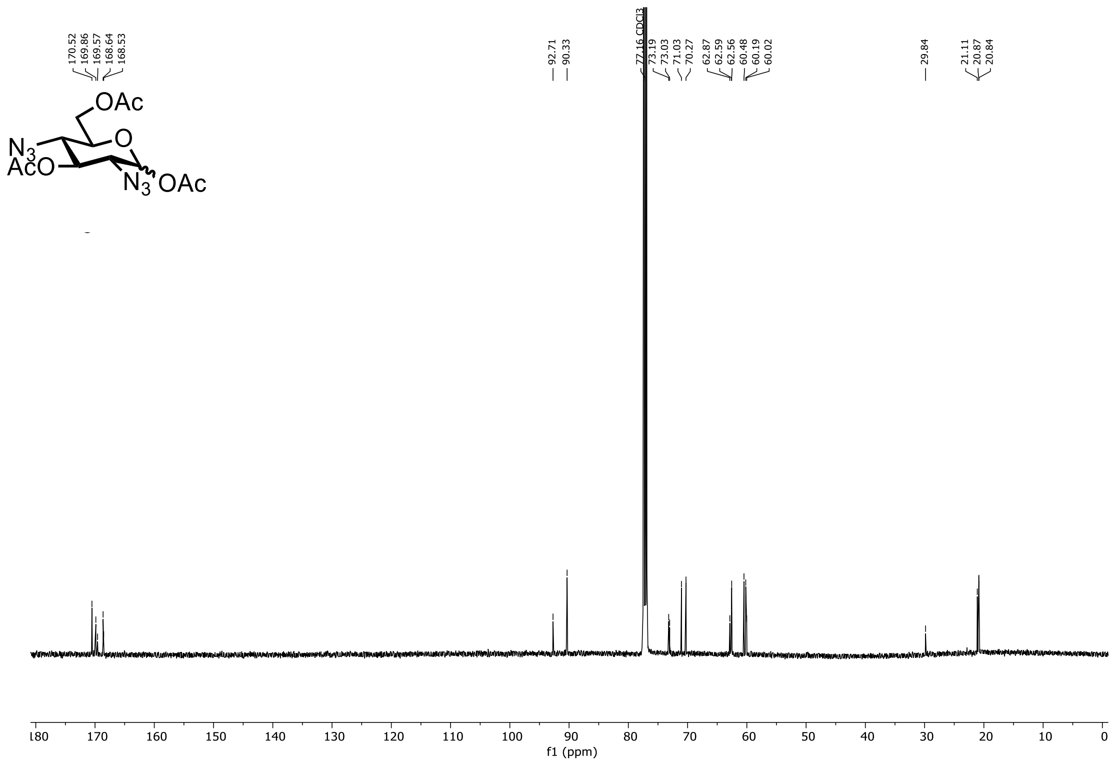

COSY spectrum of compound **4** in chloroform-*d*.

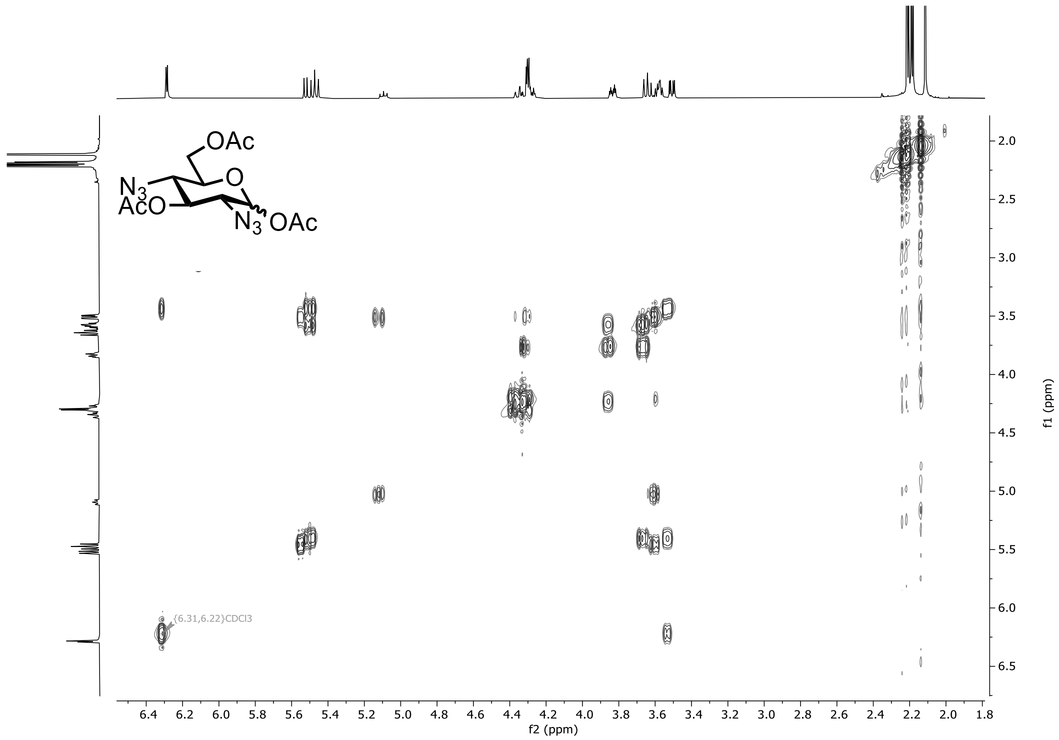

HSQC spectrum of compound **4** in chloroform-*d*.

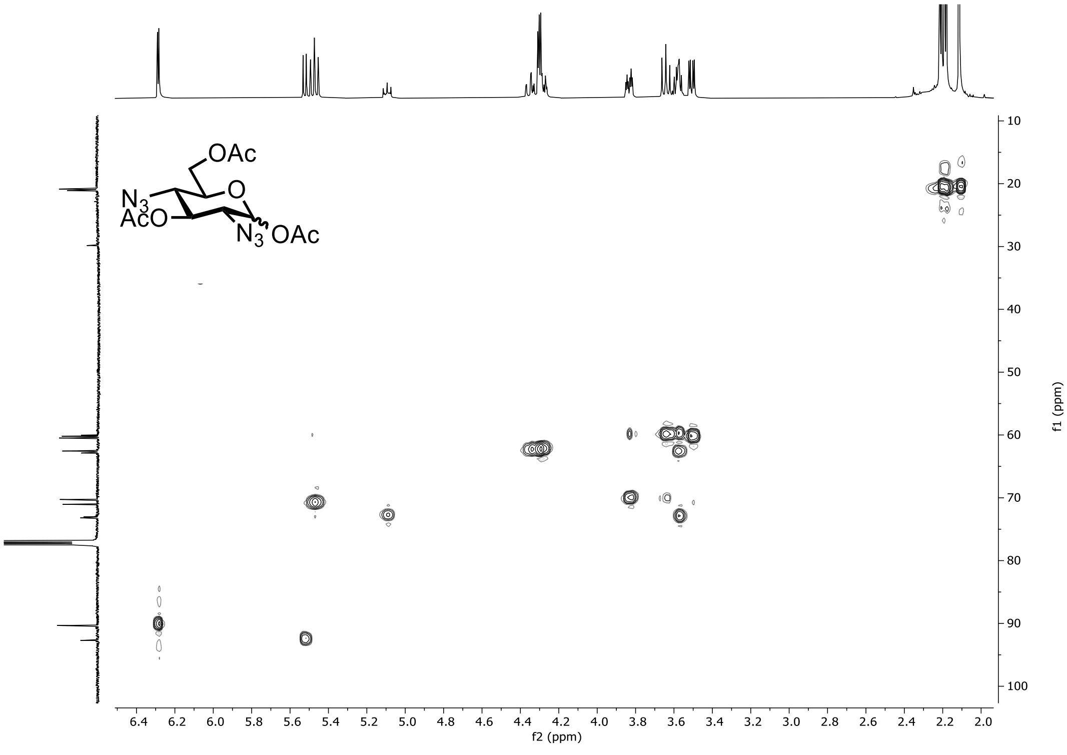

^{1}H-NMR (500 MHz) spectrum of compound **5** in methanol-d_4.

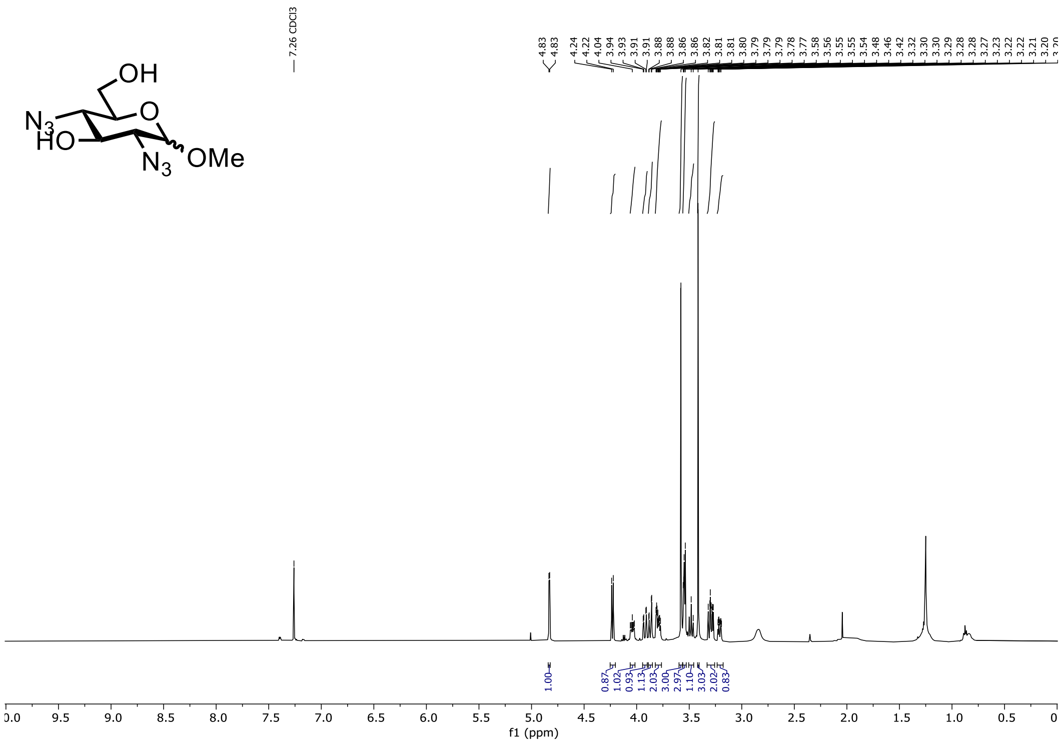

^{13}C{^{1}H}-NMR (126 MHz) spectrum of compound **5** in methanol-d_4.

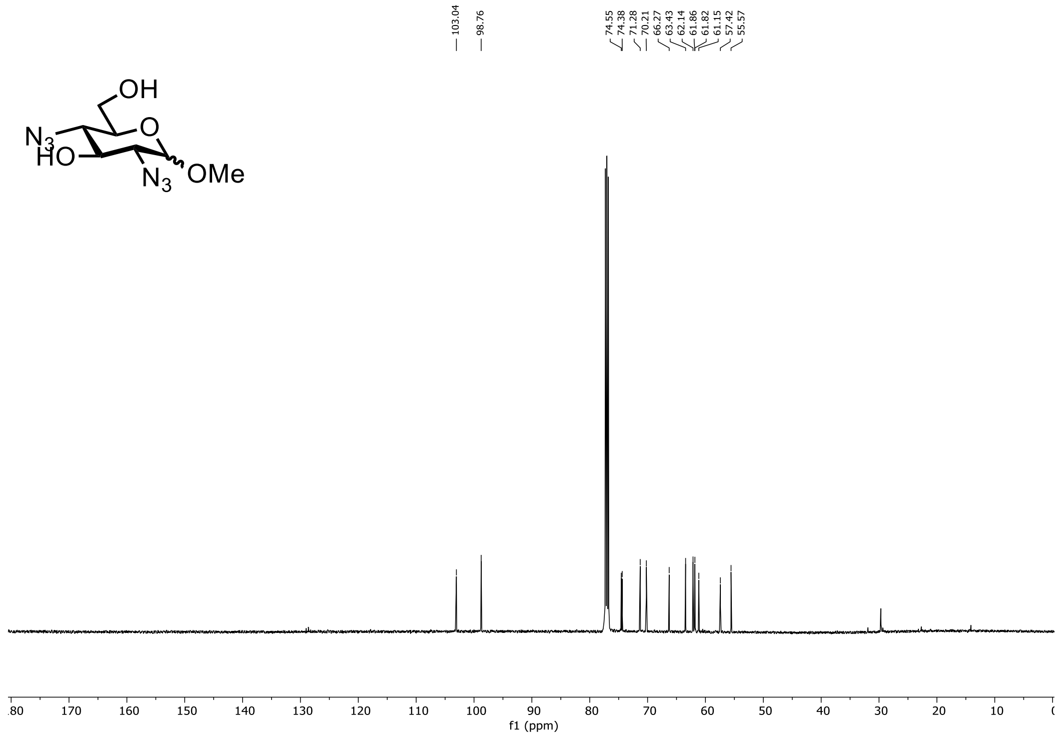

COSY spectrum of compound **5** in methanol-d_4.

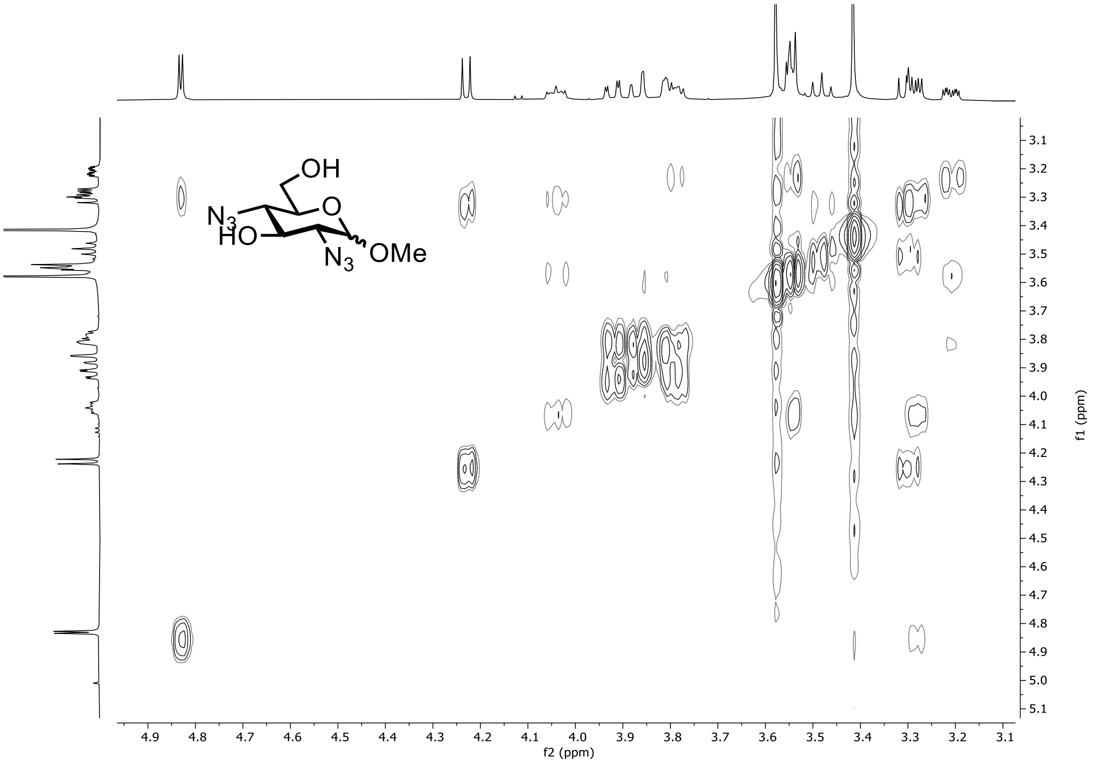

HSQC spectrum of compound **5** in methanol-d_4.

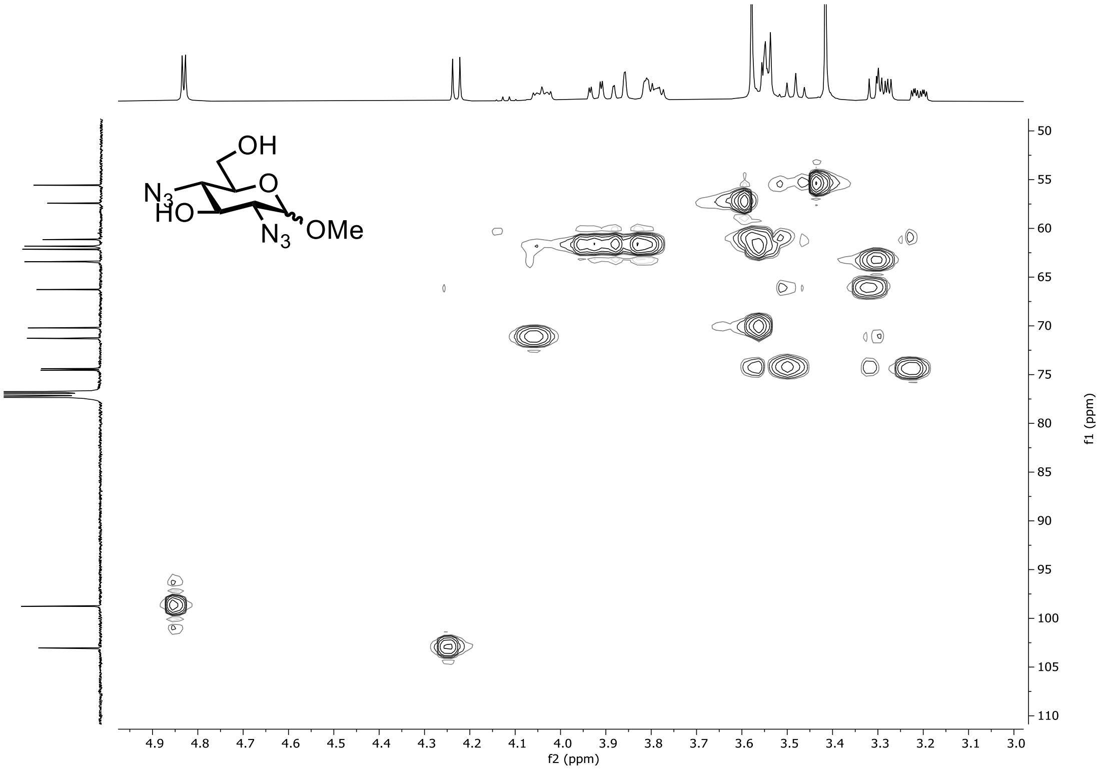

^{1}H-NMR (500 MHz) spectrum of compound **5β** in methanol-d_4.

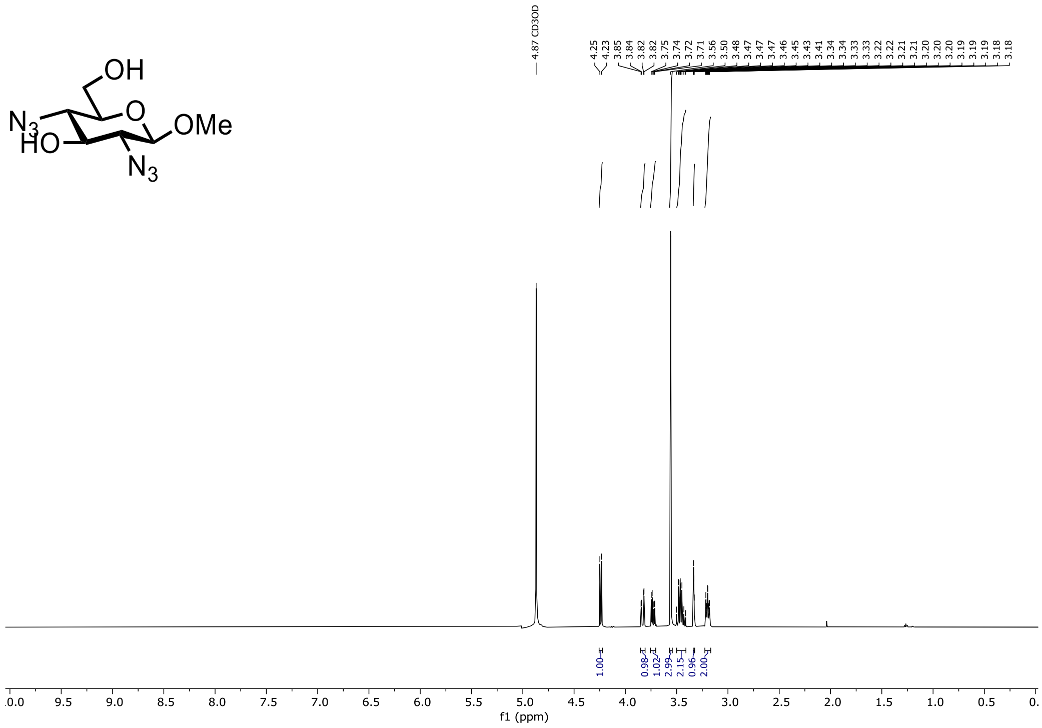

^{13}C{^{1}H}-NMR (126 MHz) spectrum of compound **5β** in methanol-d_4.

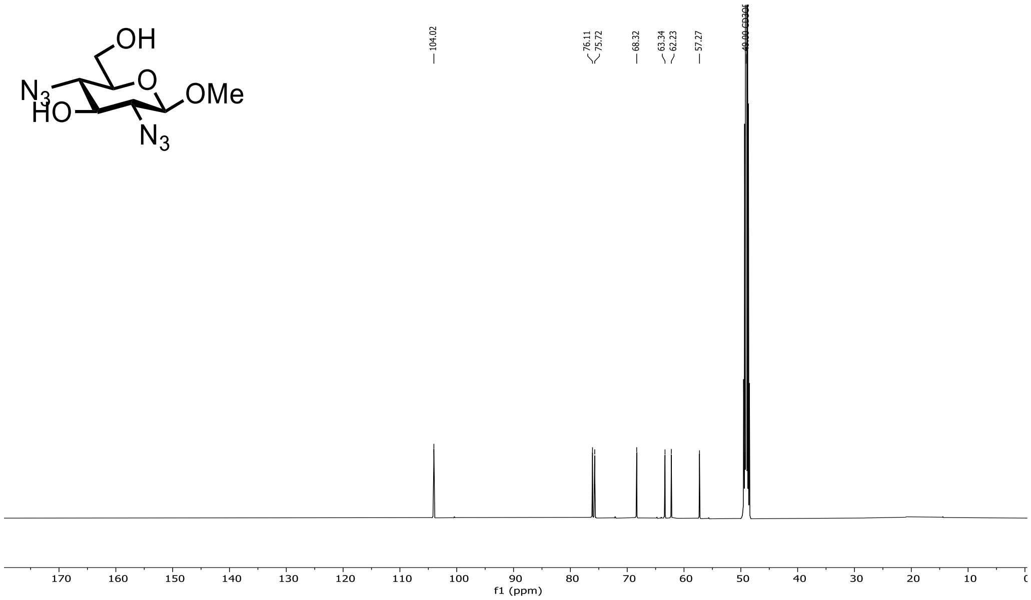

COSY spectrum of compound **5β** in methanol-d_4.

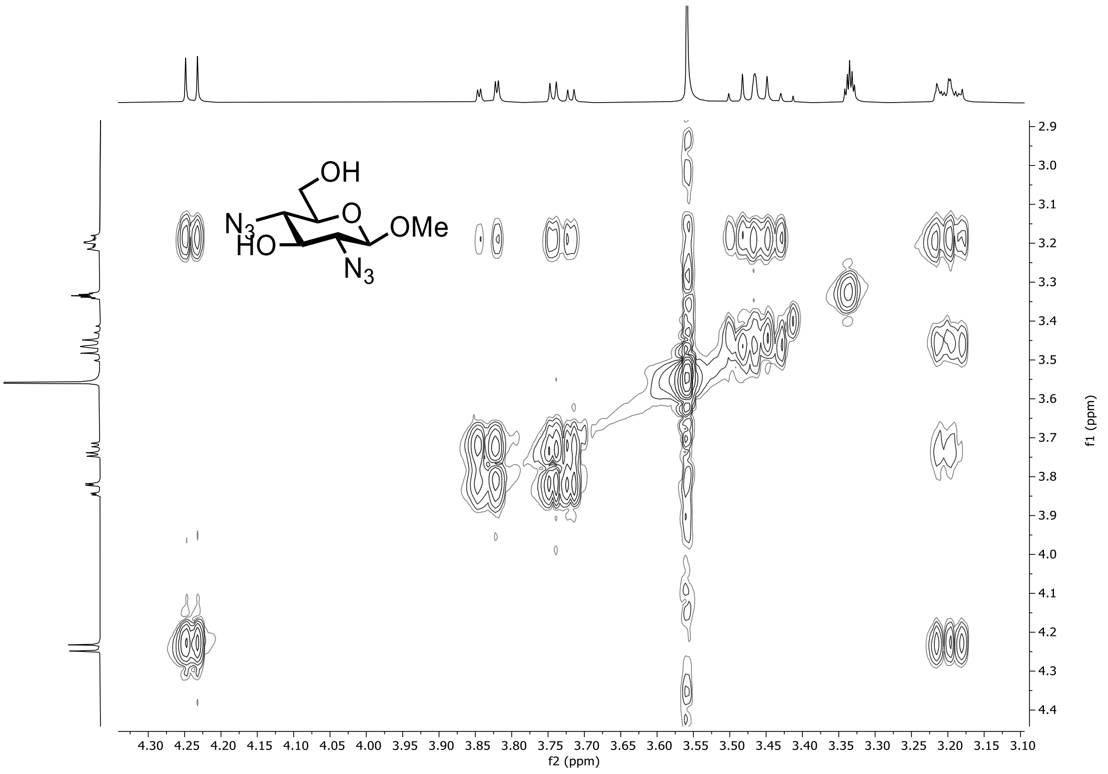

HSQC spectrum of compound **5β** in methanol-d_4.

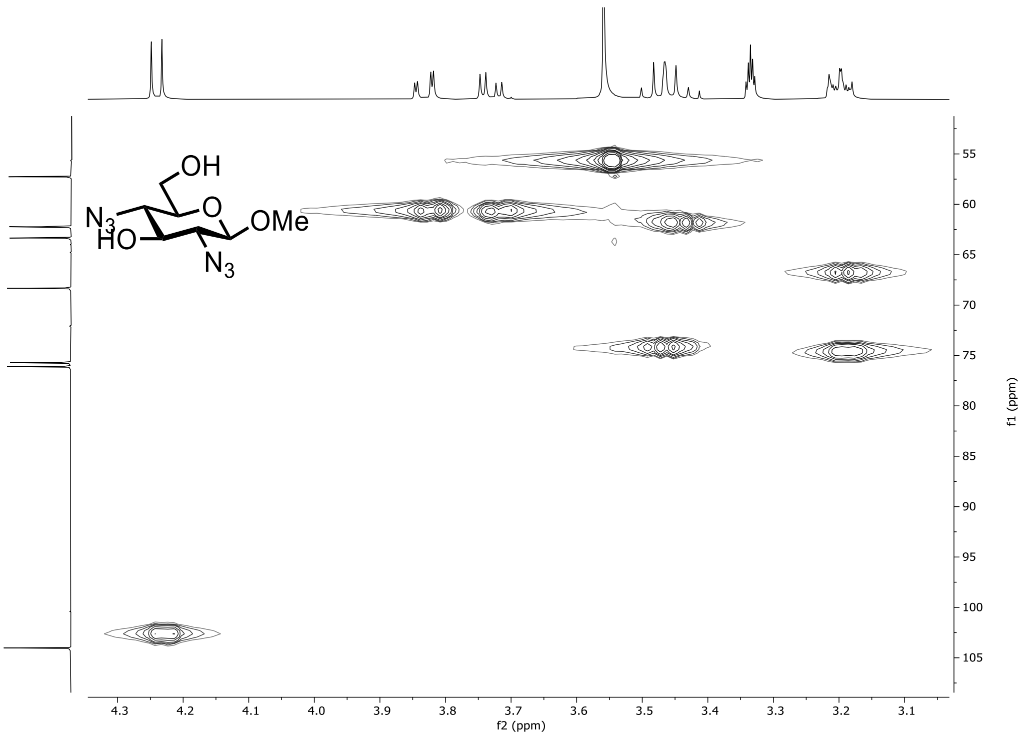

^{1}H-NMR (500 MHz) spectrum of compound **6** in chloroform-*d*.

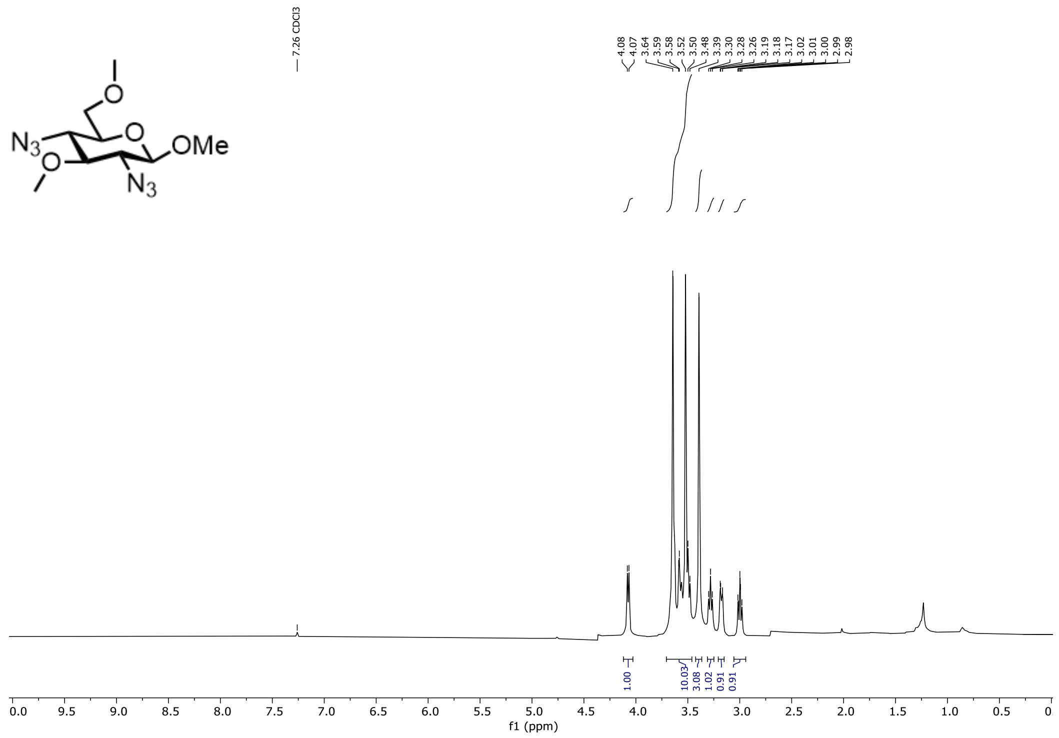

^{13}C{^{1}H}-NMR (126 MHz) spectrum of compound **6** in chloroform-*d*.

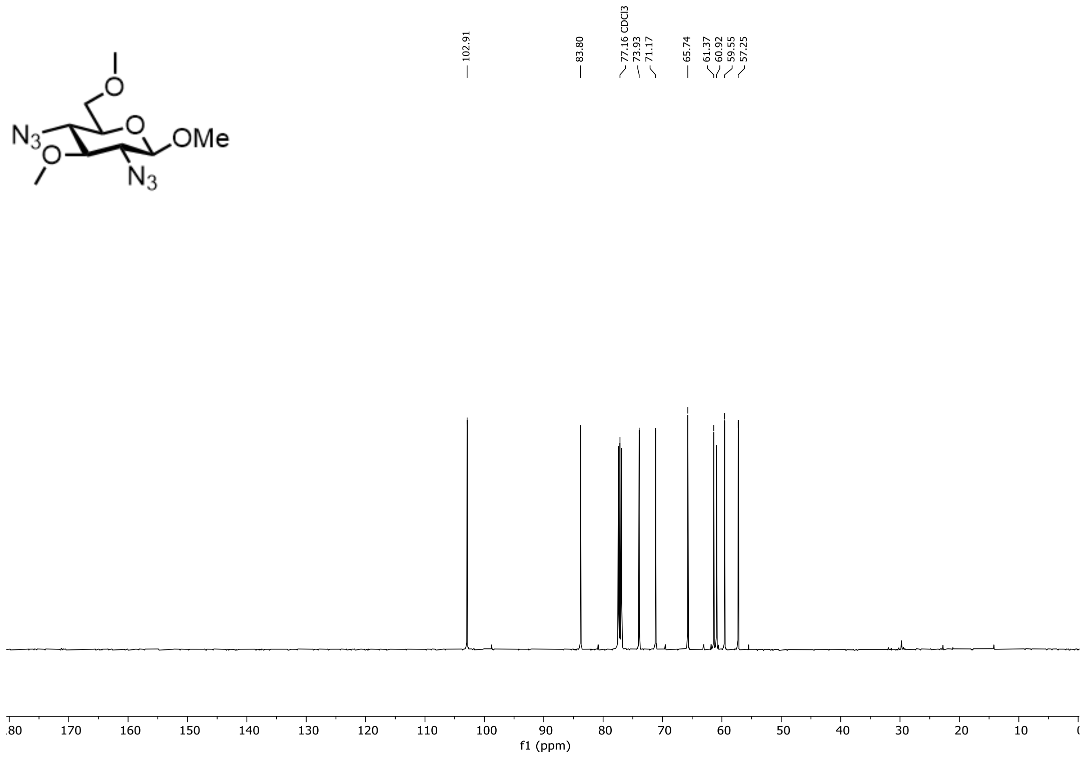

COSY spectrum of compound **6** in chloroform-*d*.

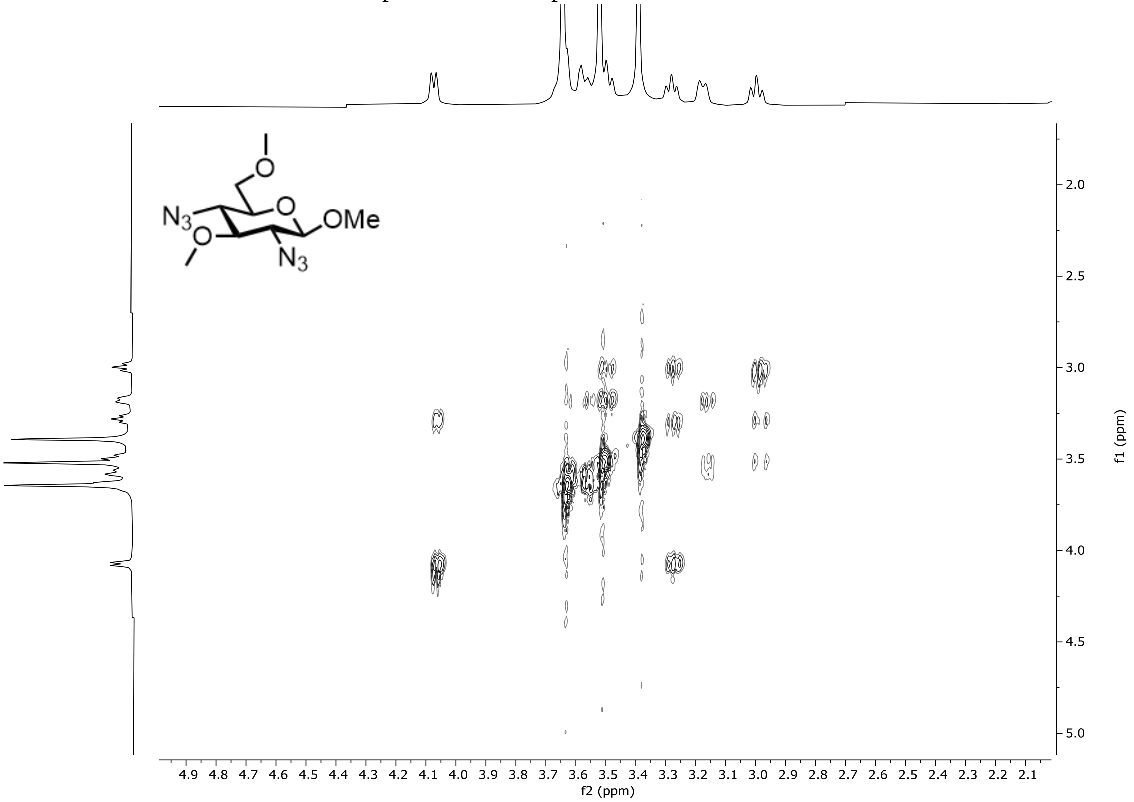

HSQC spectrum of compound **6** in chloroform-*d*.

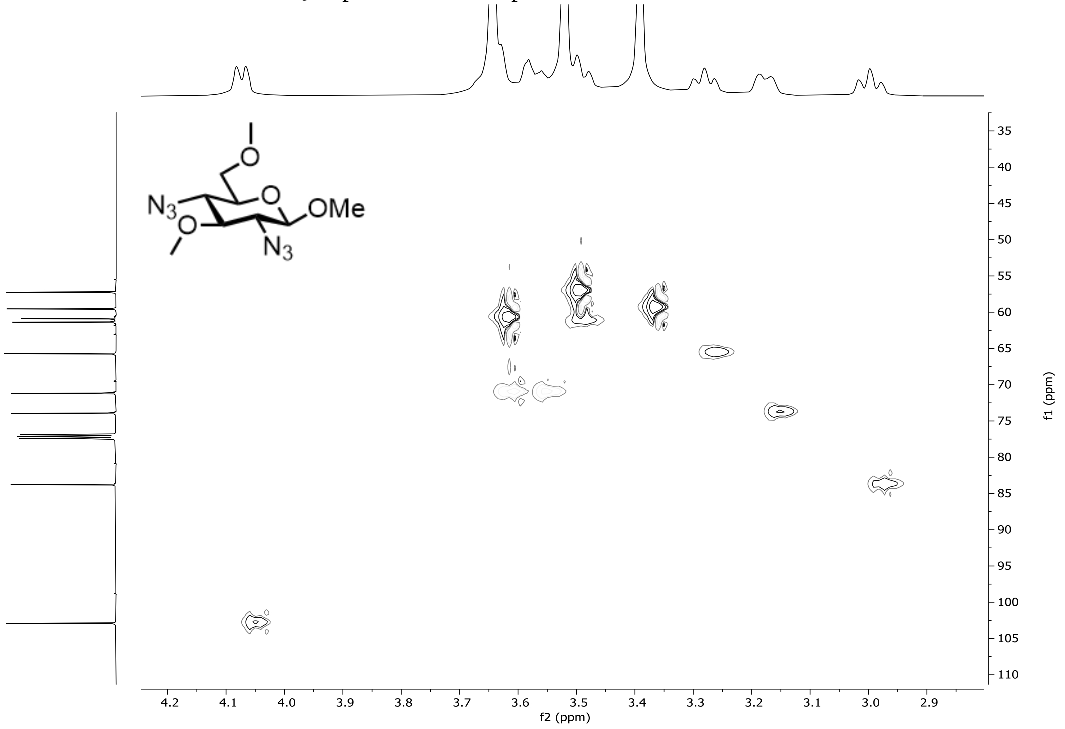

^{1}H-NMR (500 MHz) spectrum of compound **7** in chloroform-*d*.

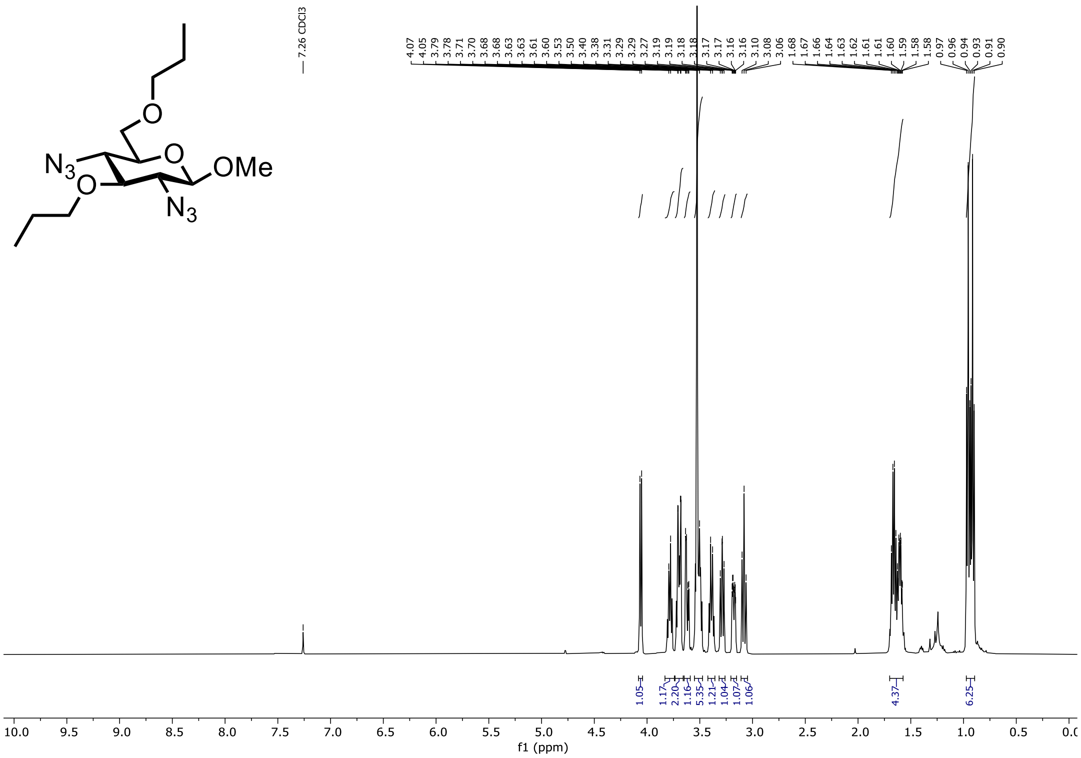

^{13}C{^{1}H}-NMR (126 MHz) spectrum of compound **7** in chloroform-*d*.

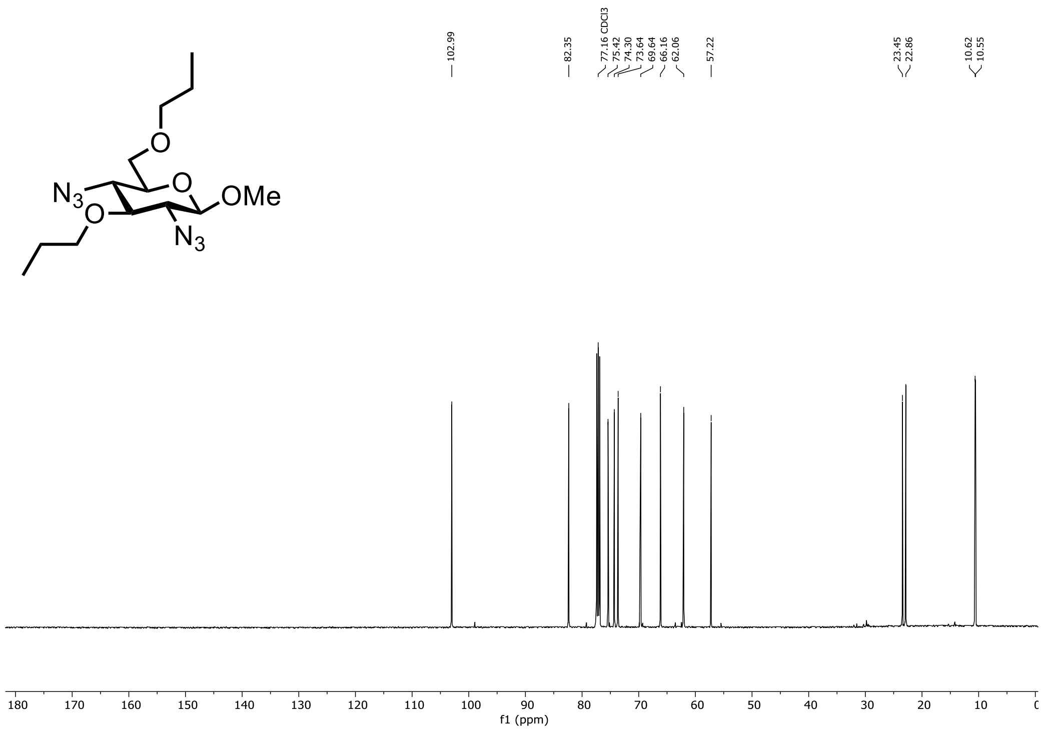

COSY spectrum of compound **7** in chloroform-*d*.

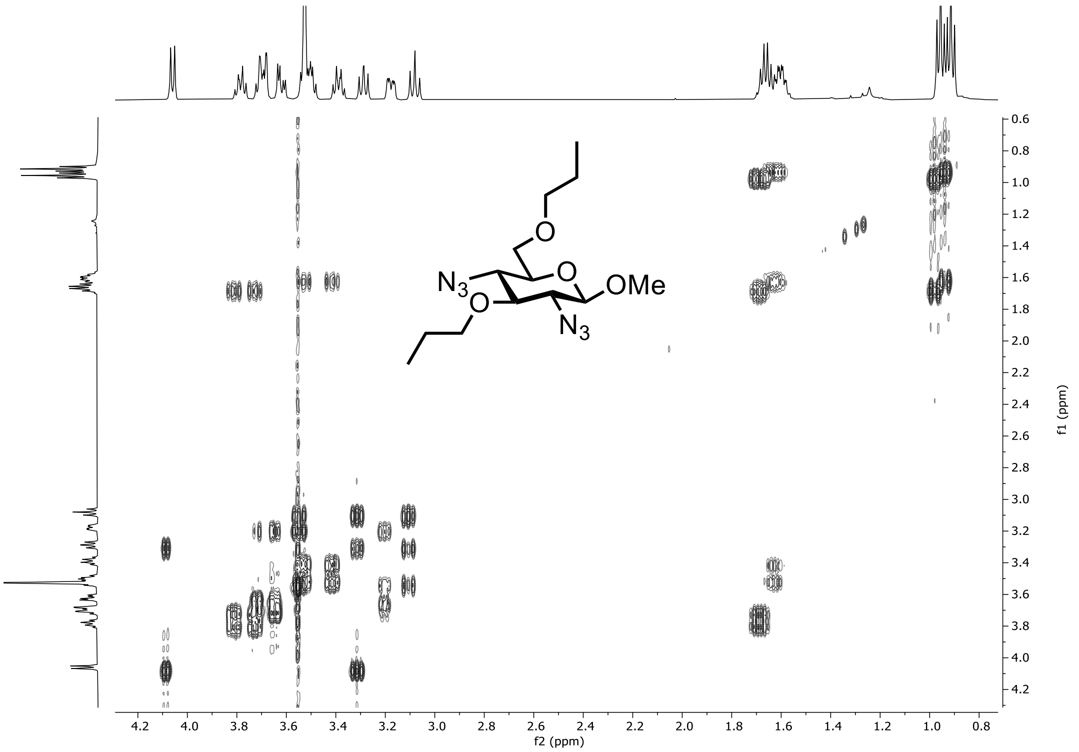

HSQC spectrum of compound **7** in chloroform-*d*.

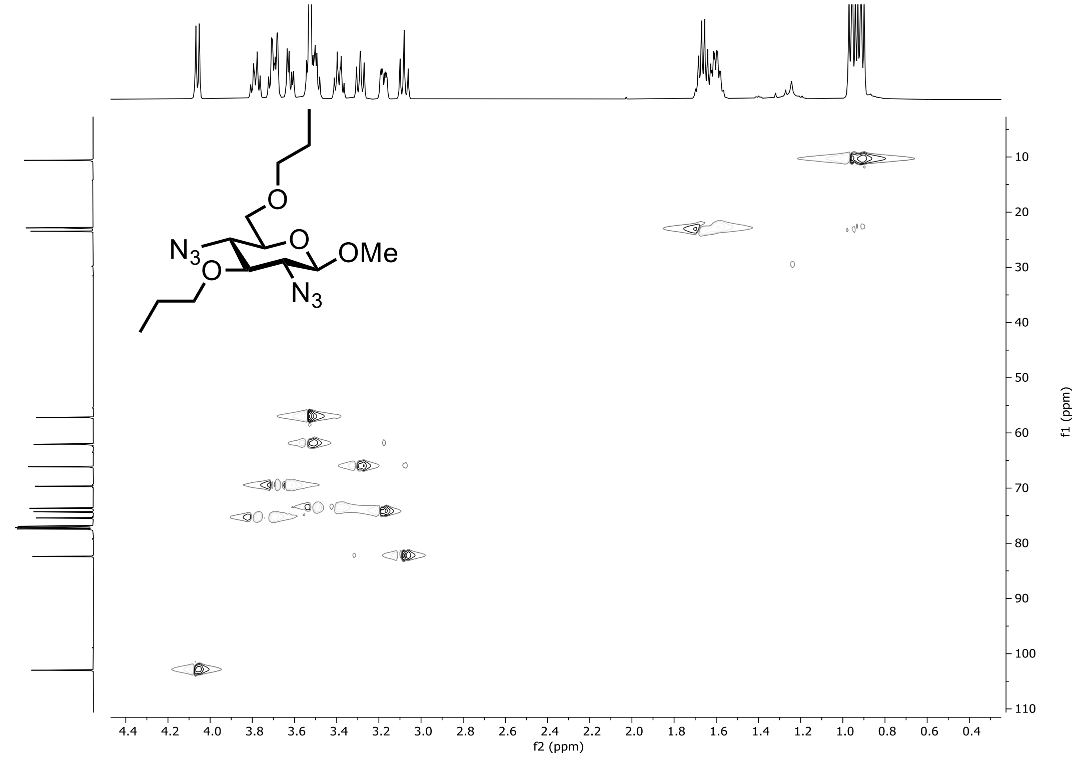

^{1}H-NMR (500 MHz) spectrum of compound **8** in chloroform-*d*.

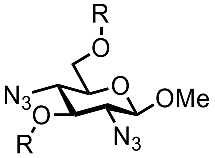

R = $(CH_2)_{11}CH_3$

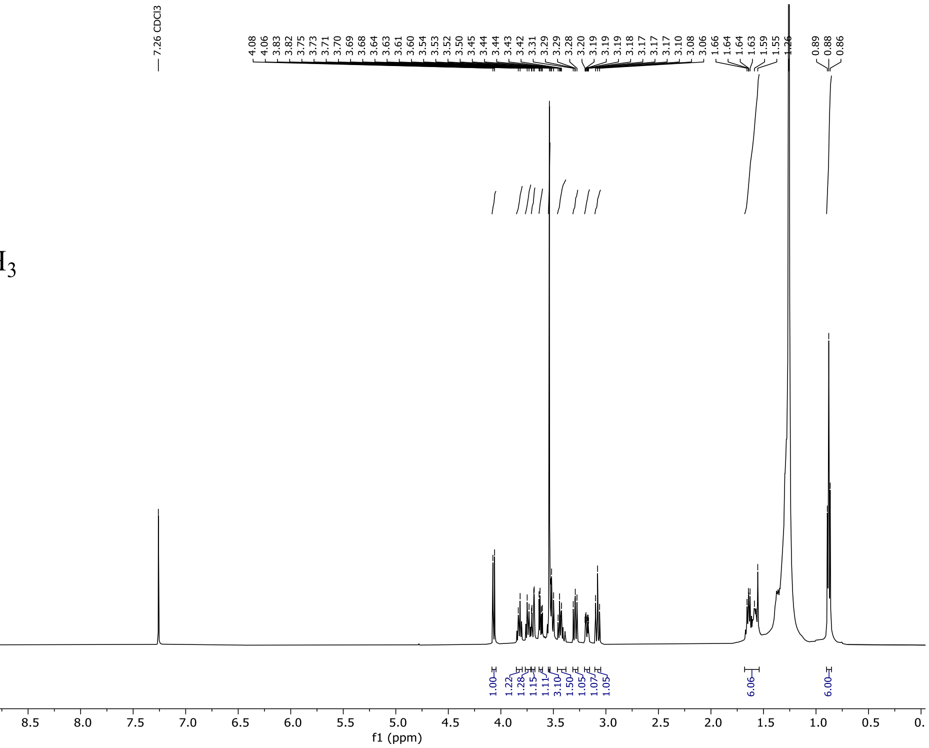

^{13}C{^{1}H}-NMR (126 MHz) spectrum of compound **8** in chloroform-*d*.

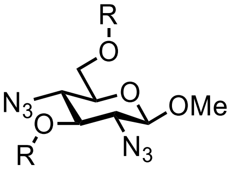

R = $(CH_2)_{11}CH_3$

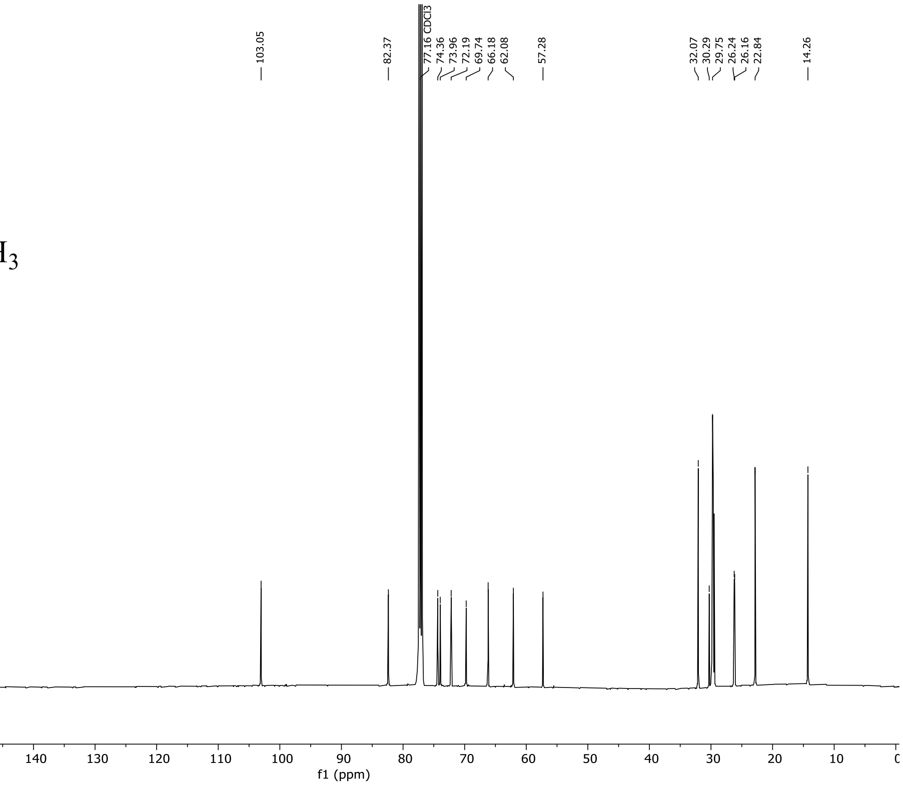

COSY spectrum of compound **8** in chloroform-*d*.

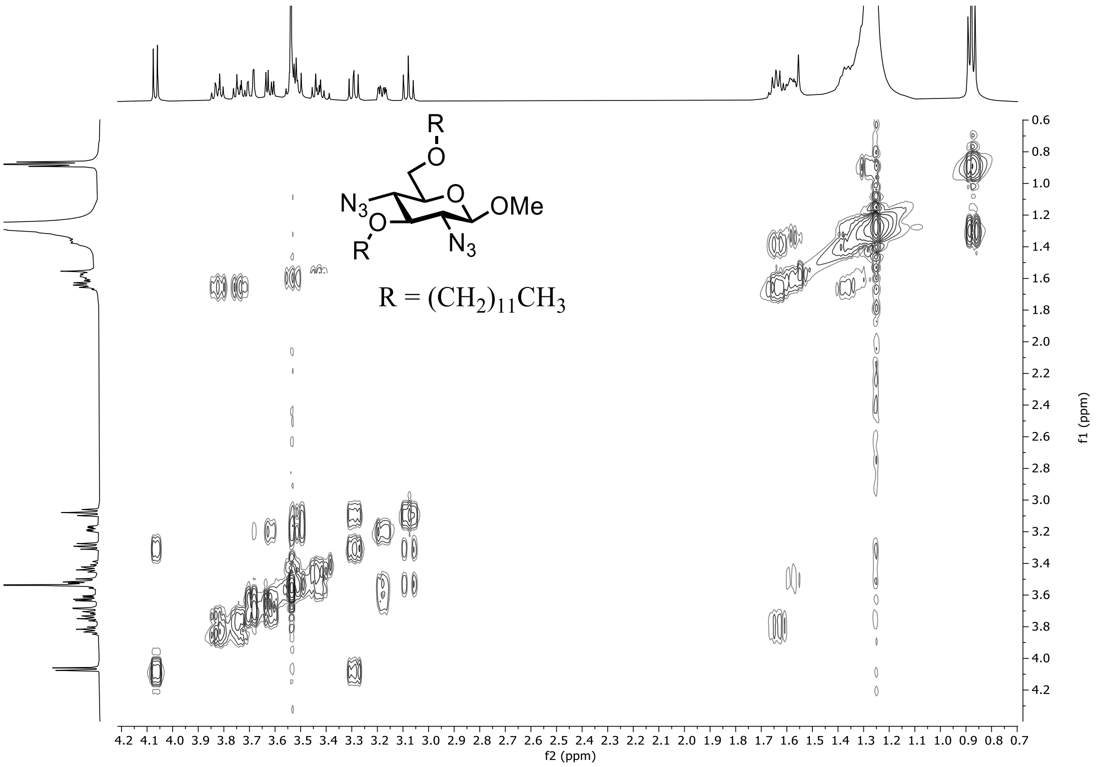

HSQC spectrum of compound **8** in chloroform-*d*.

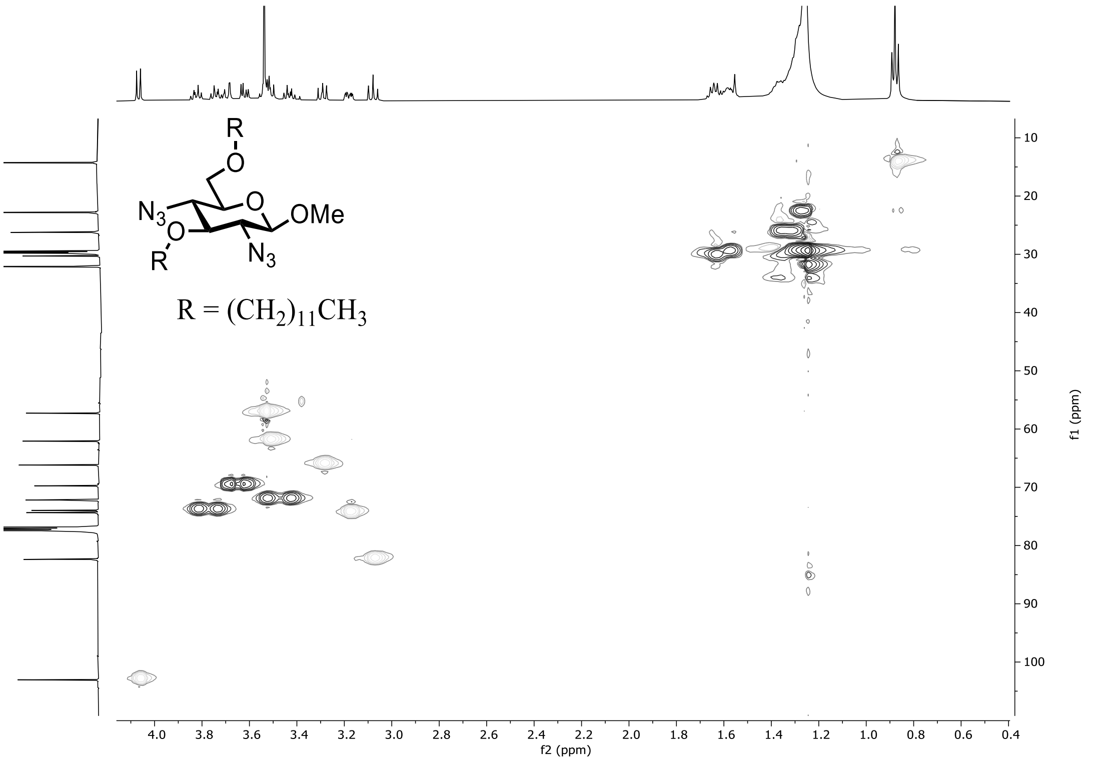

^{1}H-NMR (500 MHz) spectrum of compound **9** in acetonitrile-d_3.

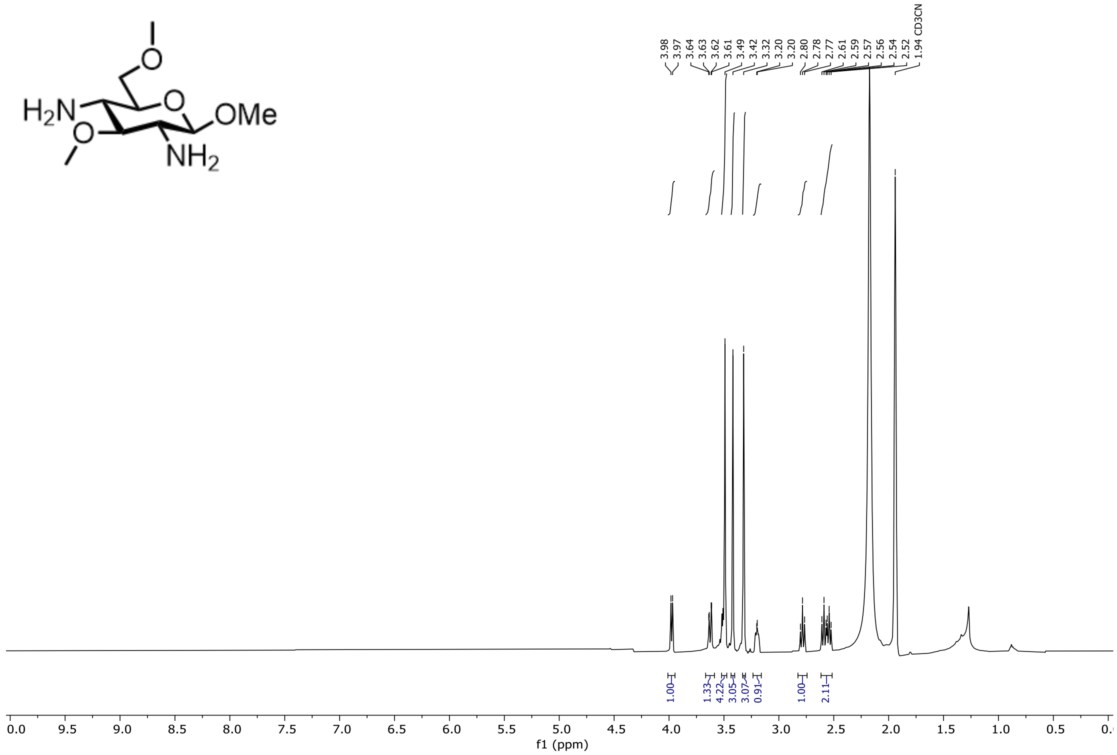

^{13}C{^{1}H}-NMR (126 MHz) spectrum of compound **9** in acetonitrile-d_3.

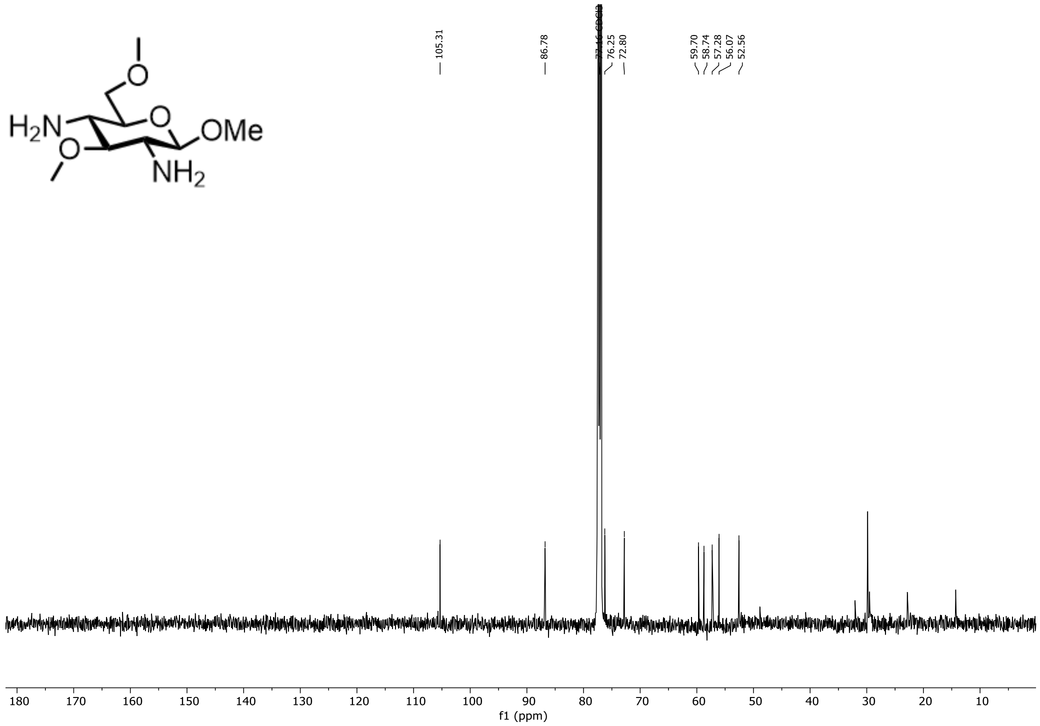

COSY spectrum of compound **9** in acetonitrile-d_3.

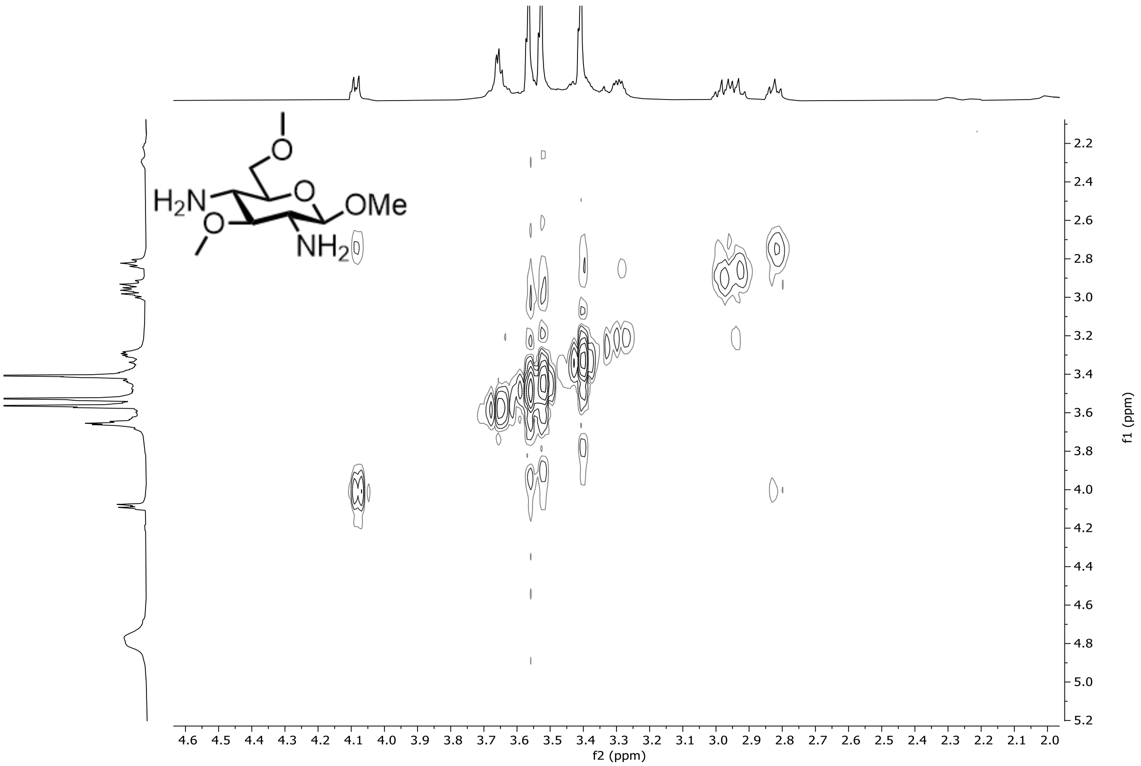

HSQC spectrum of compound **9** in acetonitrile-d_3.

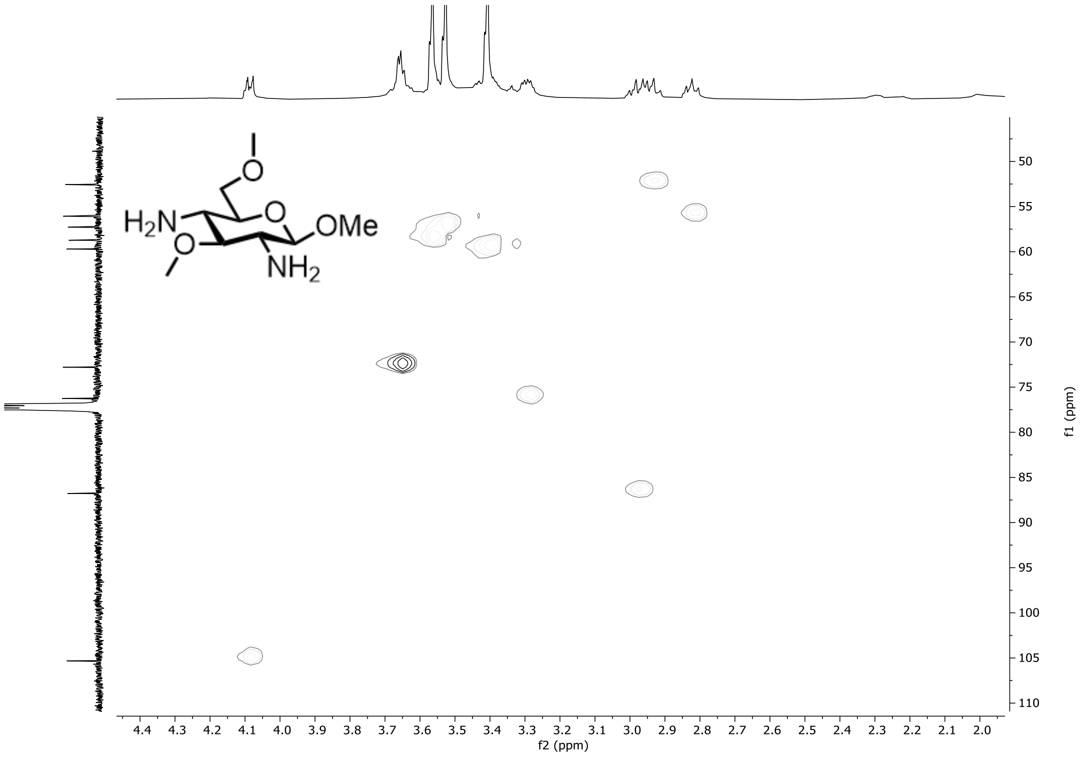

^{1}H-NMR (500 MHz) spectrum of compound **10** in acetonitrile-d_3.

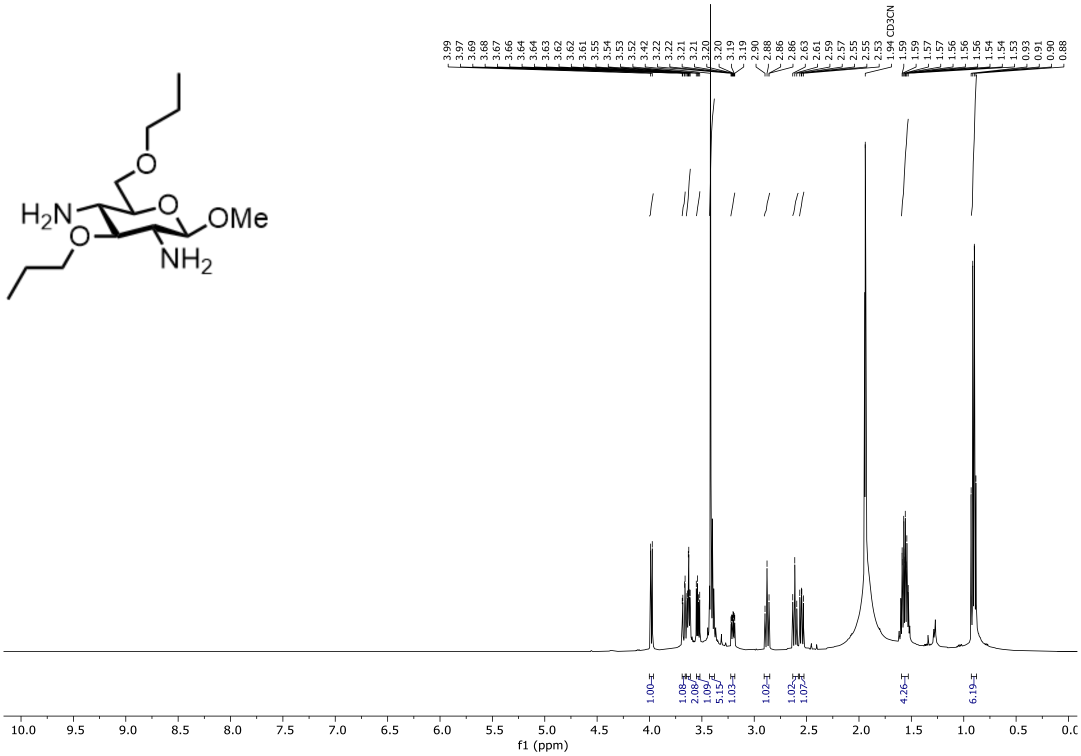

^{13}C{^{1}H}-NMR (126 MHz) spectrum of compound **10** in acetonitrile-d_3.

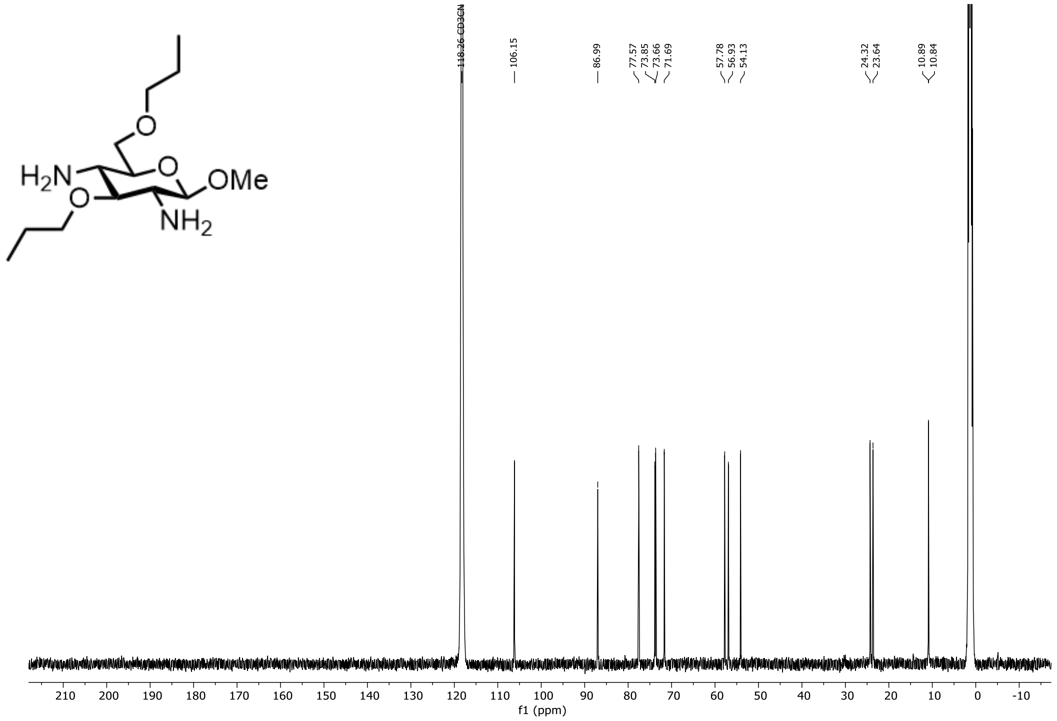

COSY spectrum of compound **10** in acetonitrile-d_3.

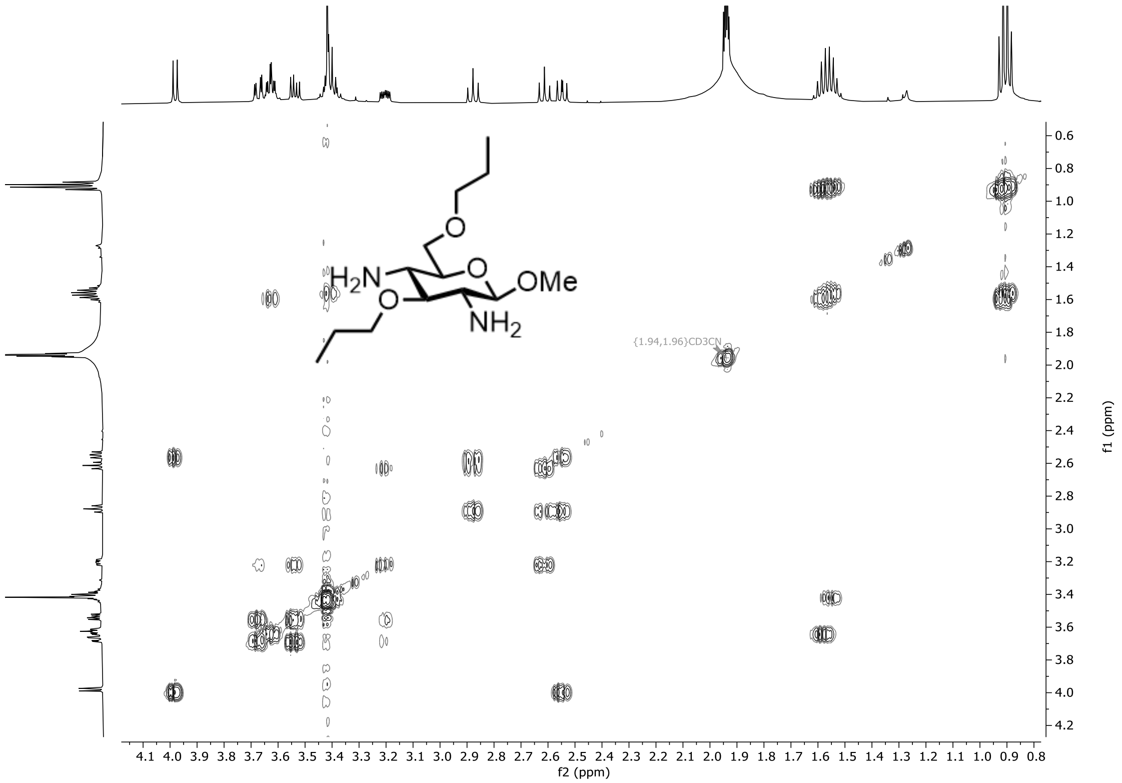

HSQC spectrum of compound **10** in acetonitrile-d_3.

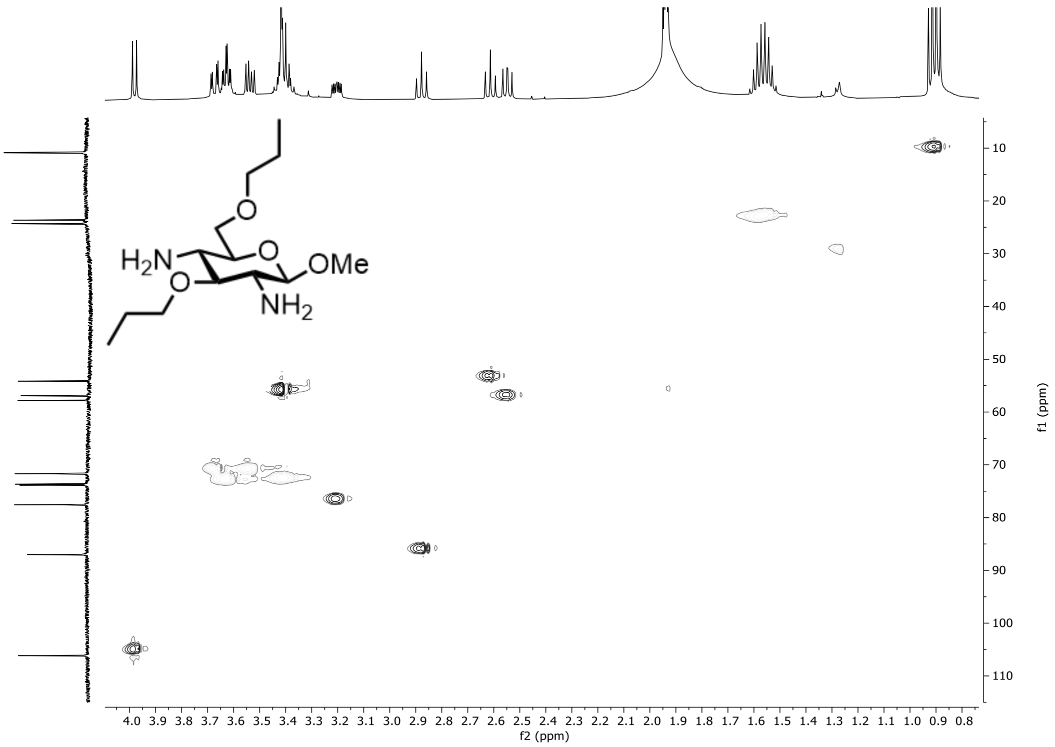

^{1}H-NMR (500 MHz) spectrum of compound **11** in acetonitrile-d_3.

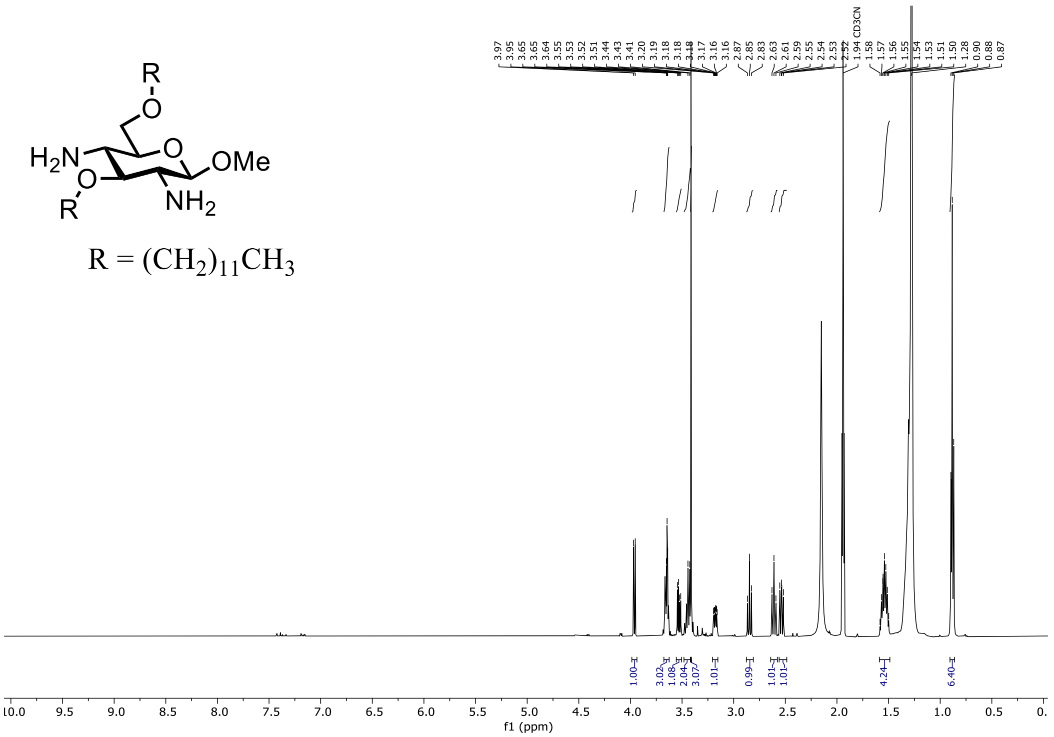

COSY spectrum of compound **11** in acetonitrile-d_3.

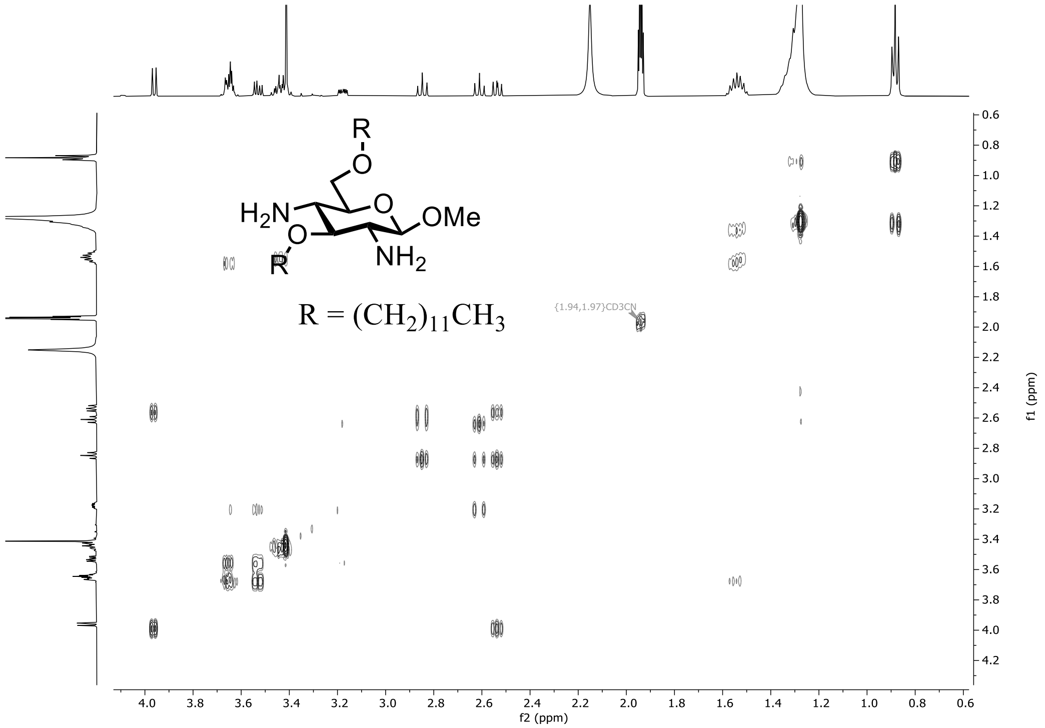

HSQC spectrum of compound **11** in acetonitrile-d_3.

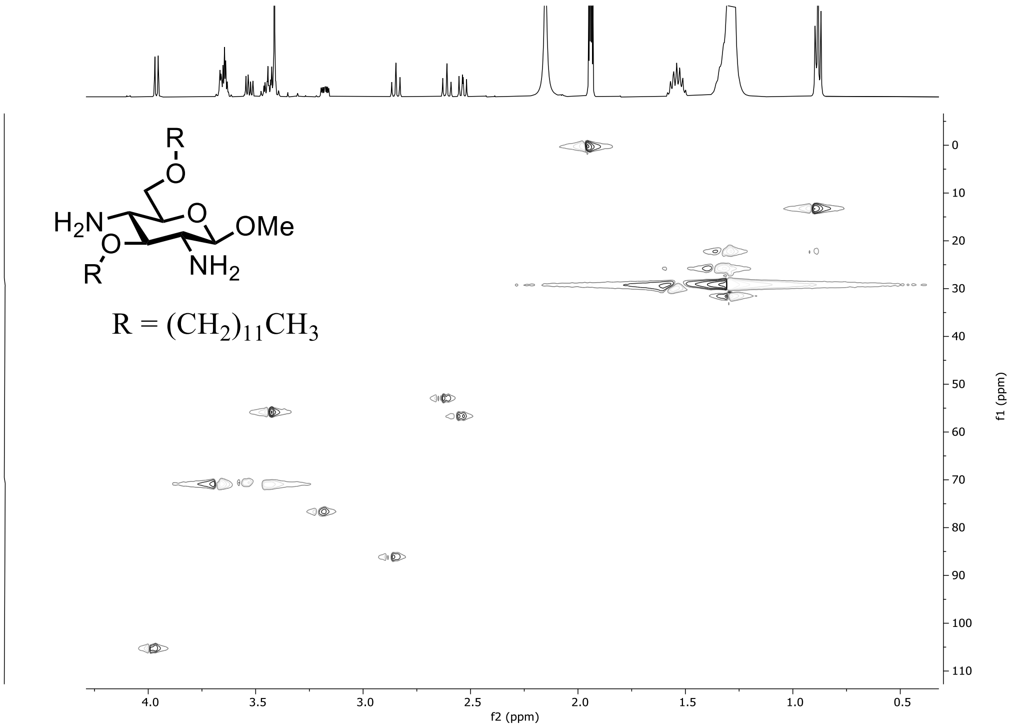

^{1}H-NMR (500 MHz) spectrum of compound **12** in chloroform-*d*.

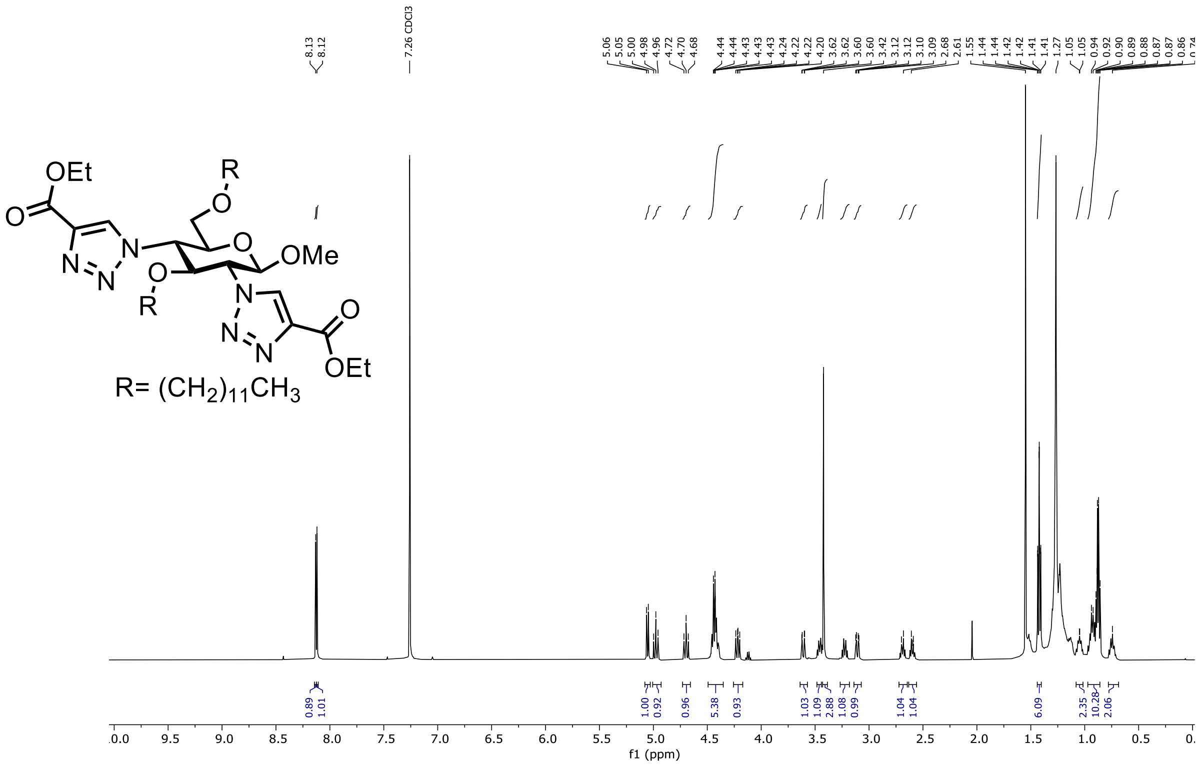

$^{13}C\{^1H\}$-NMR (126 MHz) spectrum of compound **12** in chloroform-*d*.

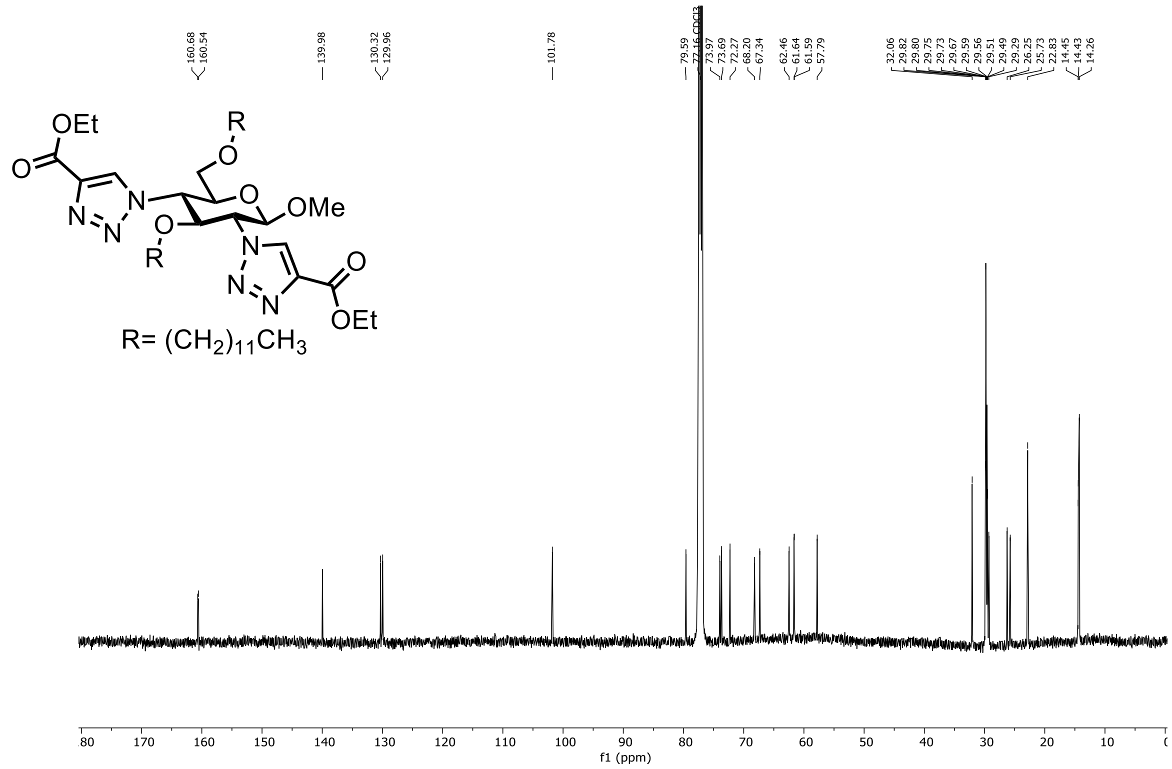

COSY spectrum of compound **12** in chloroform-*d*.

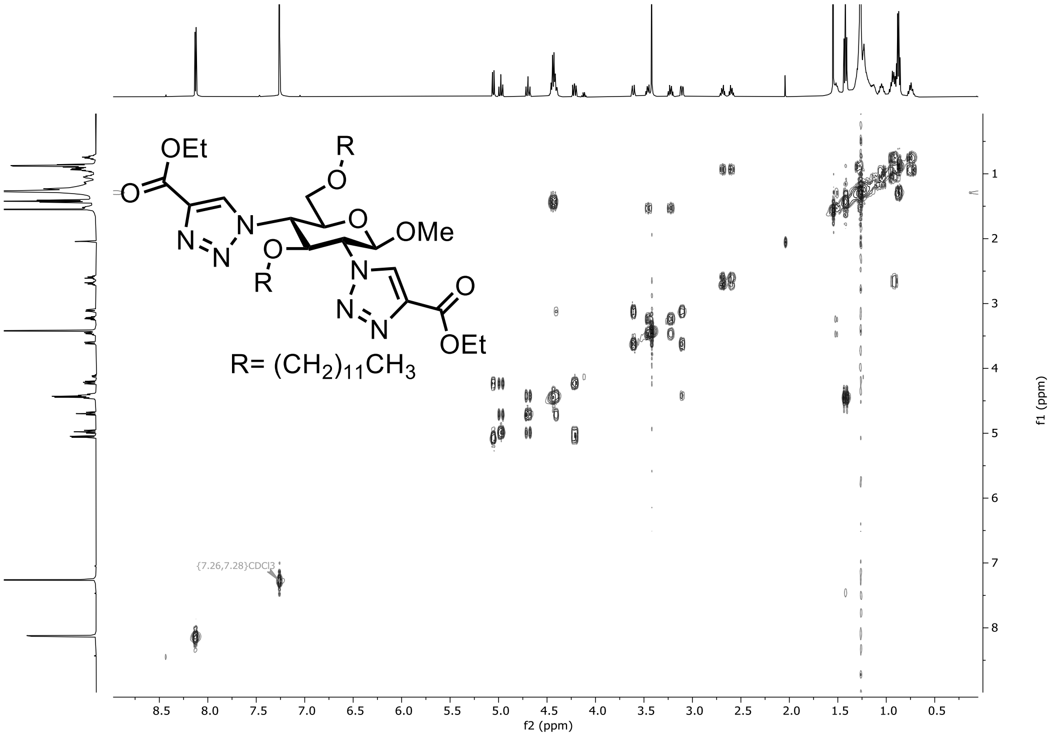

HSQC spectrum of compound **12** in chloroform-*d*.

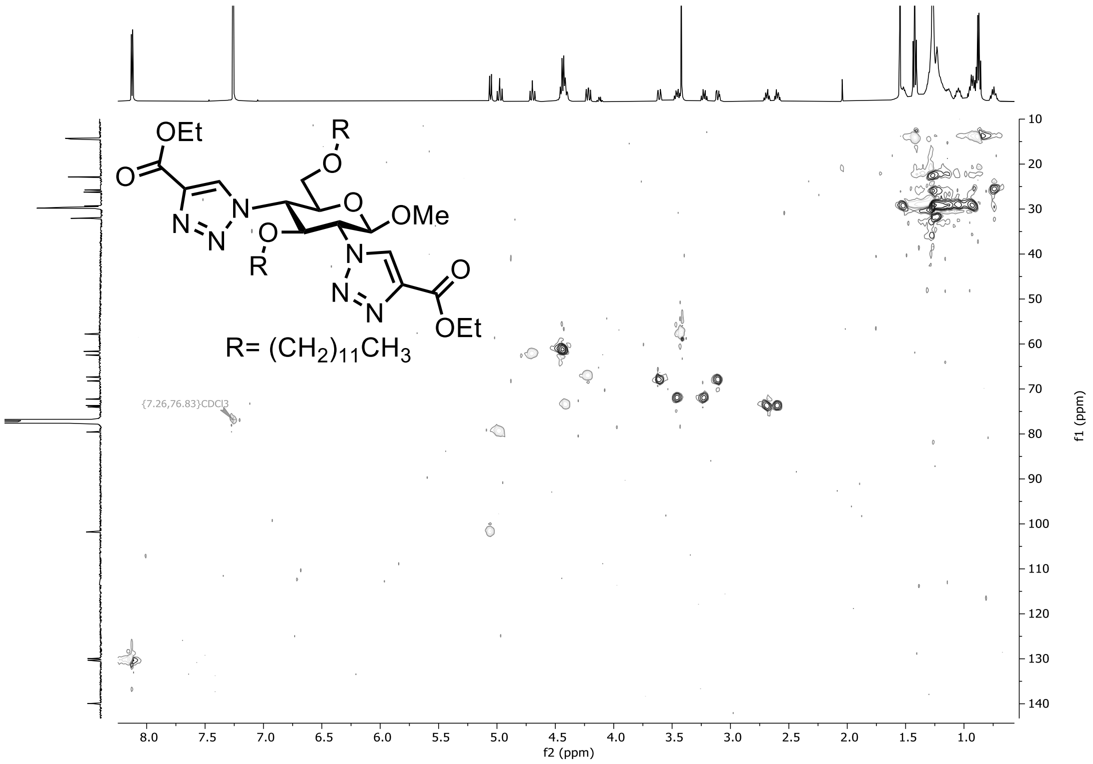

^{1}H-NMR (500 MHz) spectrum of compound **16** in acetonitrile-d_3.

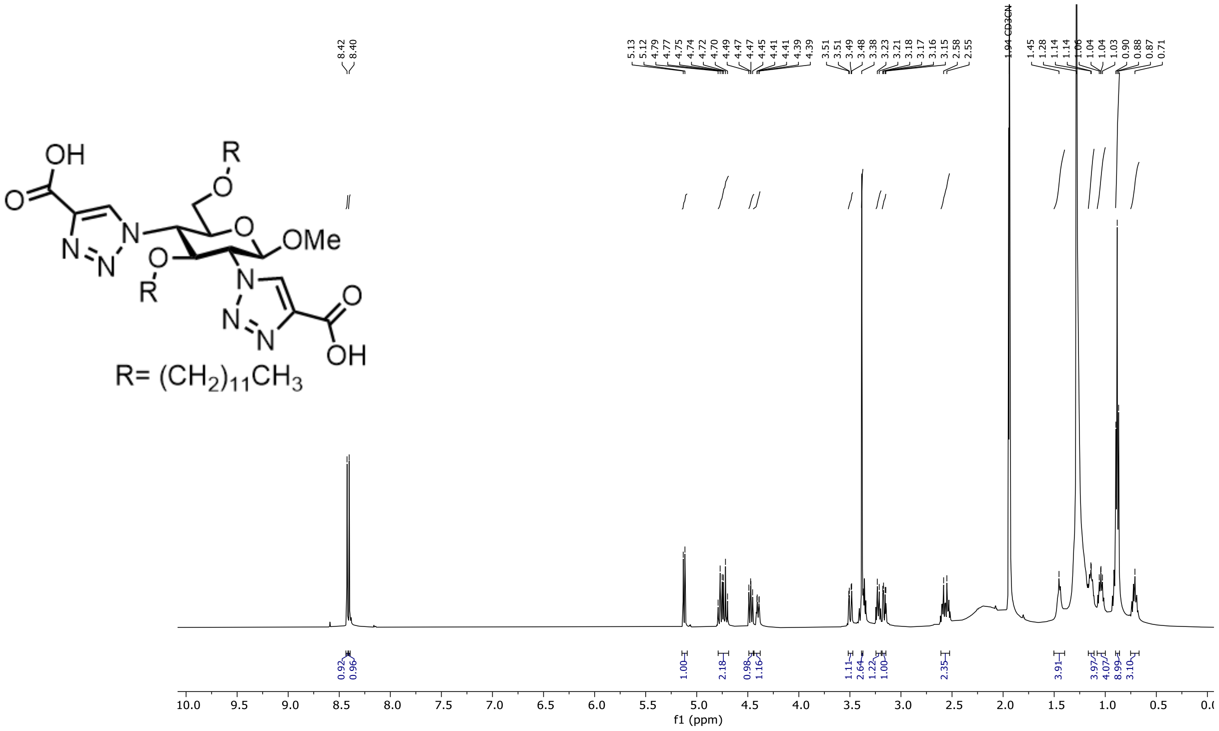

$^{13}C\{^{1}H\}$-NMR (126 MHz) spectrum of compound **16** in acetonitrile-*d*$_3$.

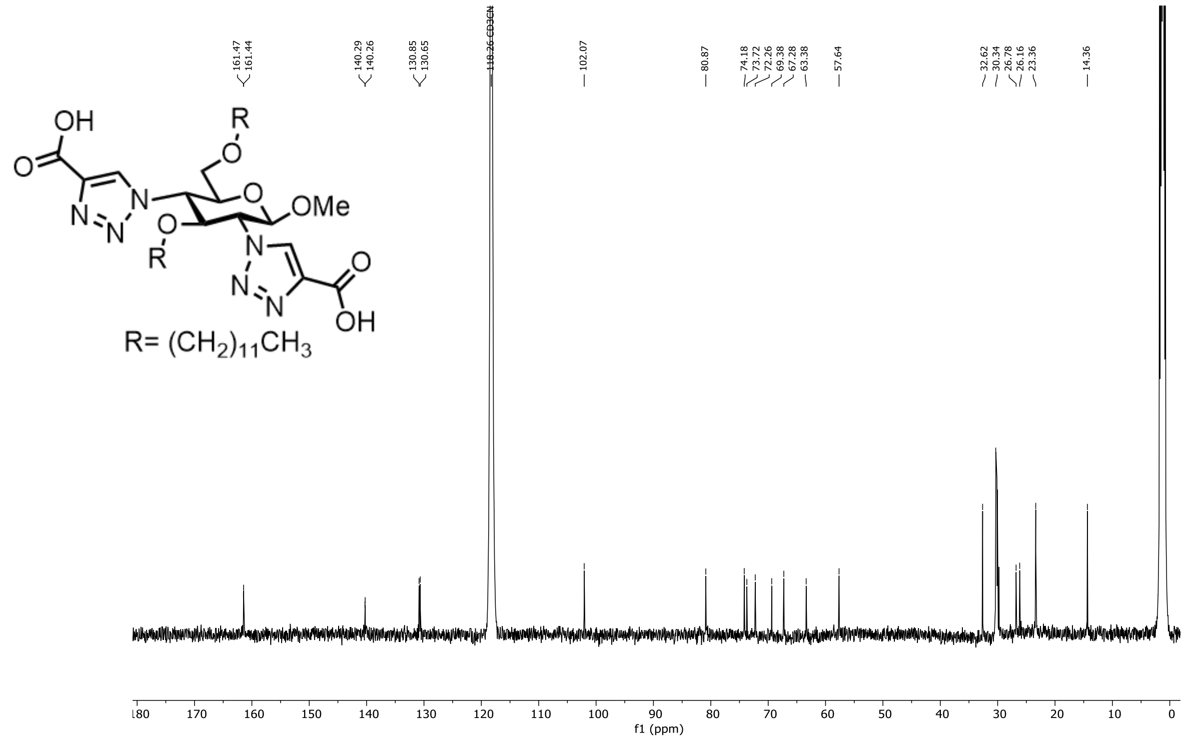

COSY spectrum of compound **16** in acetonitrile-*d*$_3$.

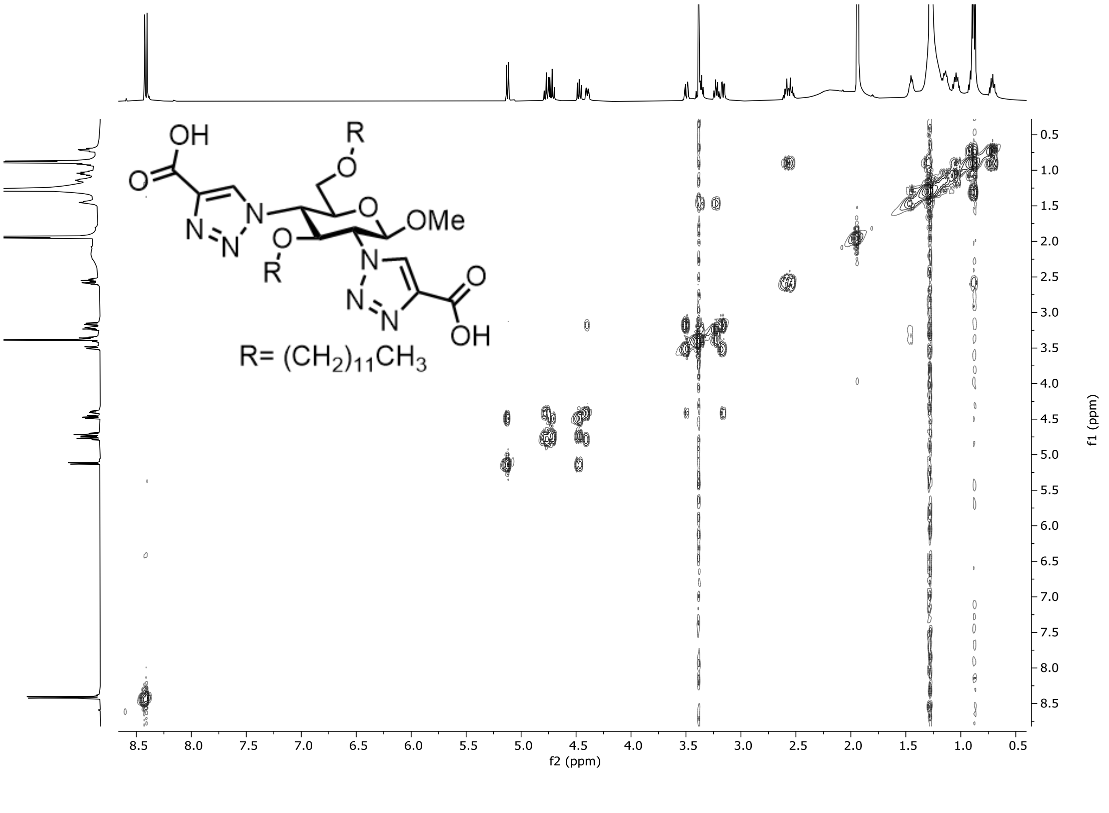

HSQC spectrum of compound **16** in acetonitrile-d_3.

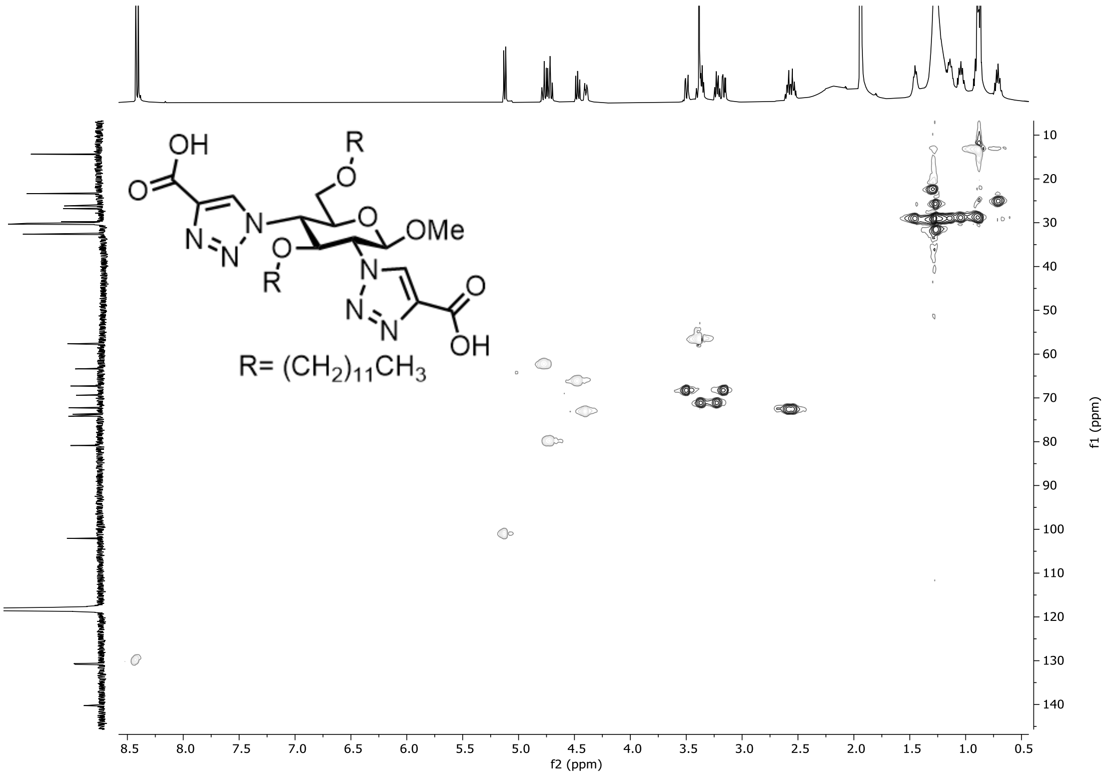

^{1}H-NMR (500 MHz) spectrum of compound **17** in acetonitrile-d_3**.**

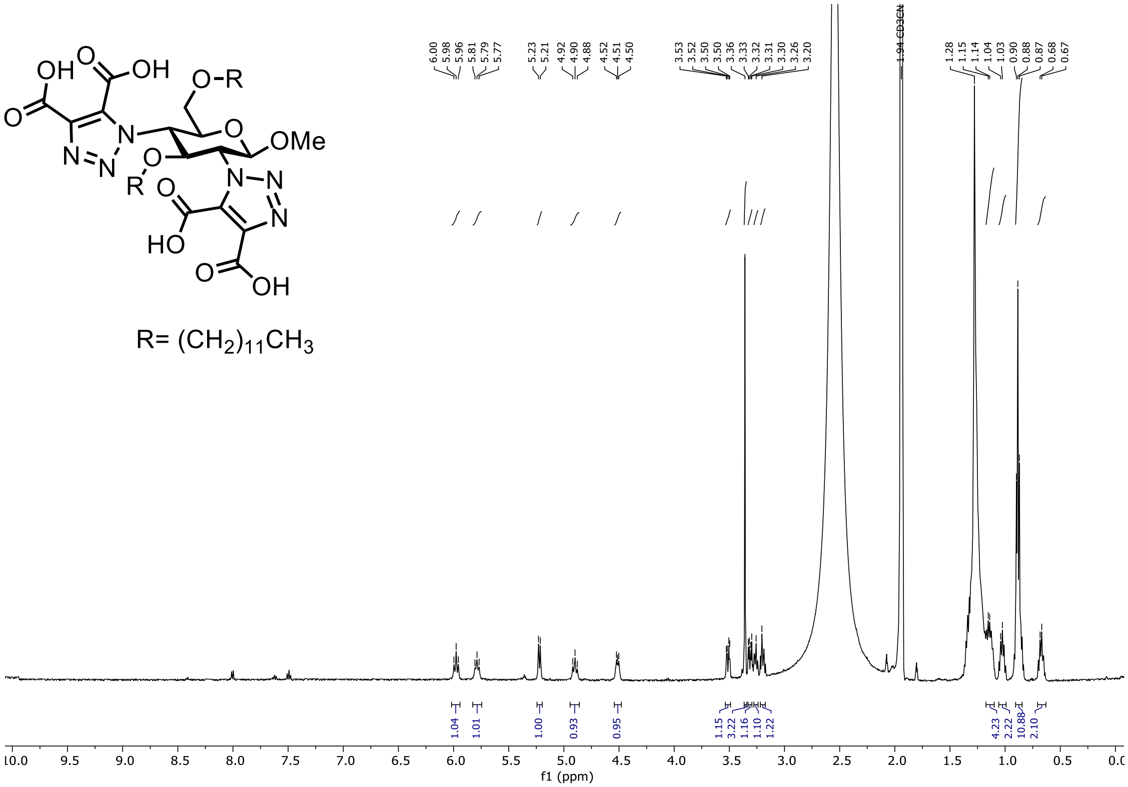

$^{13}C\{^{1}H\}$-NMR (126 MHz) spectrum of compound **17** in acetonitrile-d_3. (There are signals missing due to the low concentration of compound in the NMR sample).

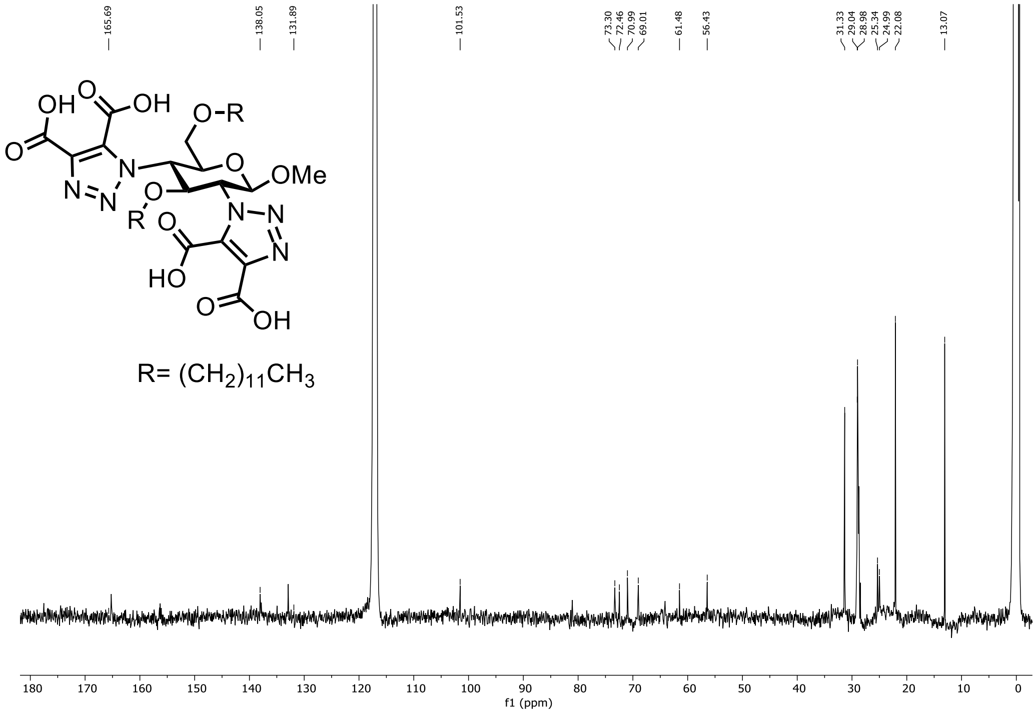

COSY spectrum of compound **17** in acetonitrile-d_3.

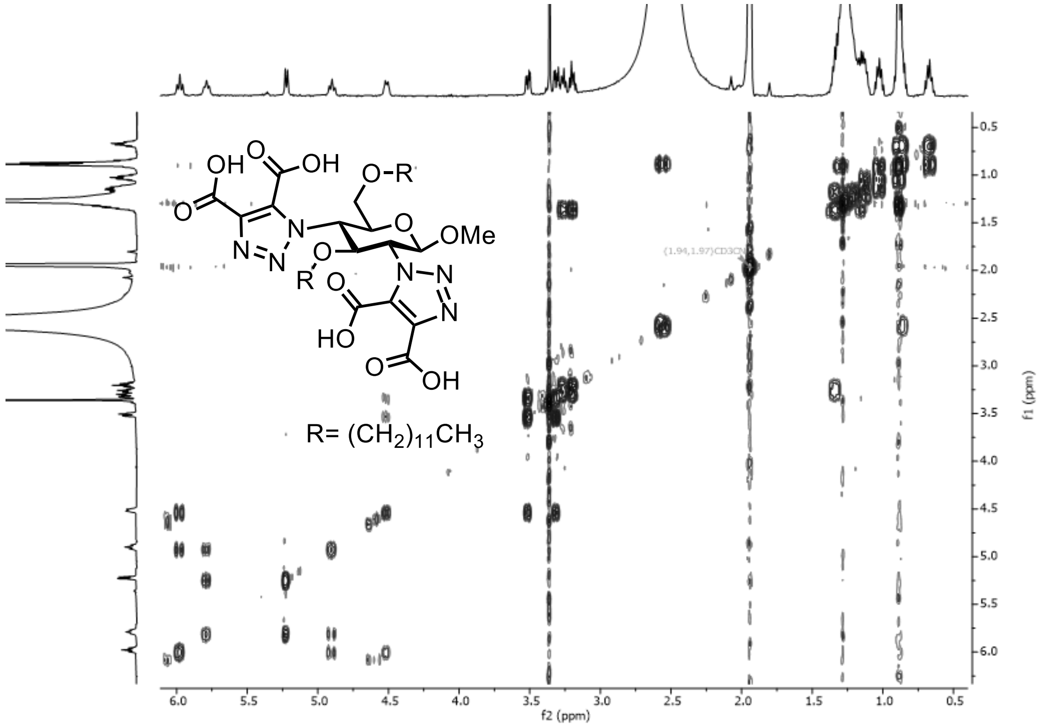

HSQC spectrum of compound **17** in acetonitrile-d_3.

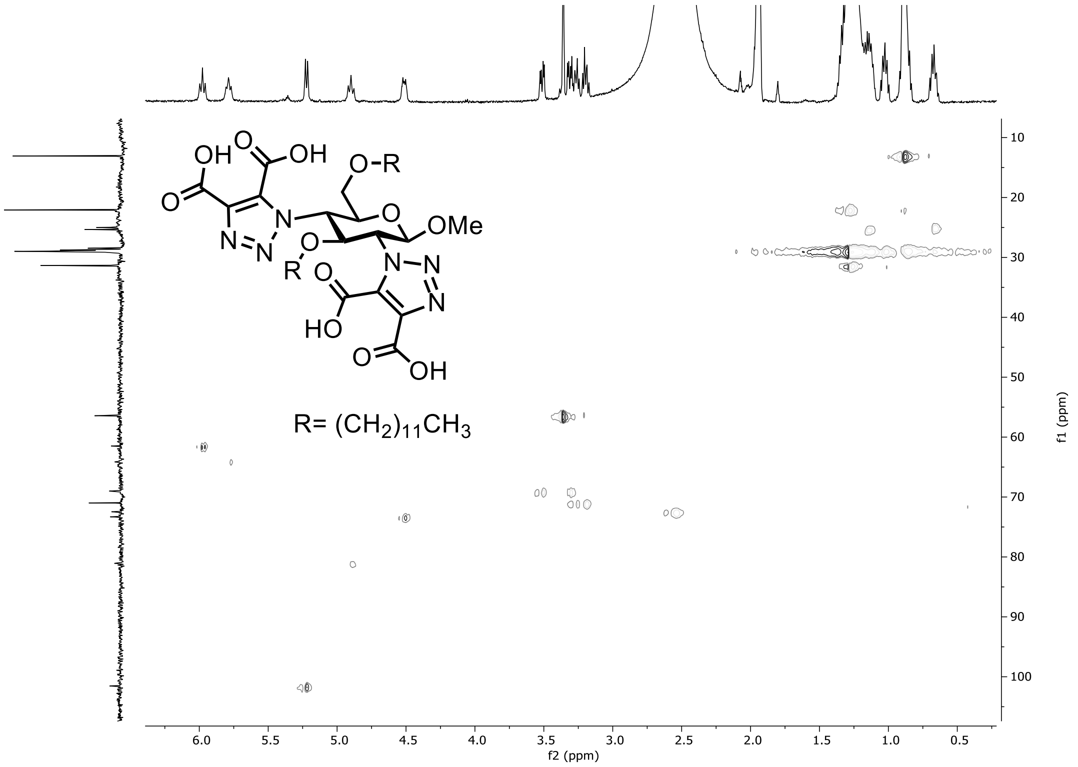

^{1}H-NMR (500 MHz) spectrum of compound **18α** in chloroform-*d*.

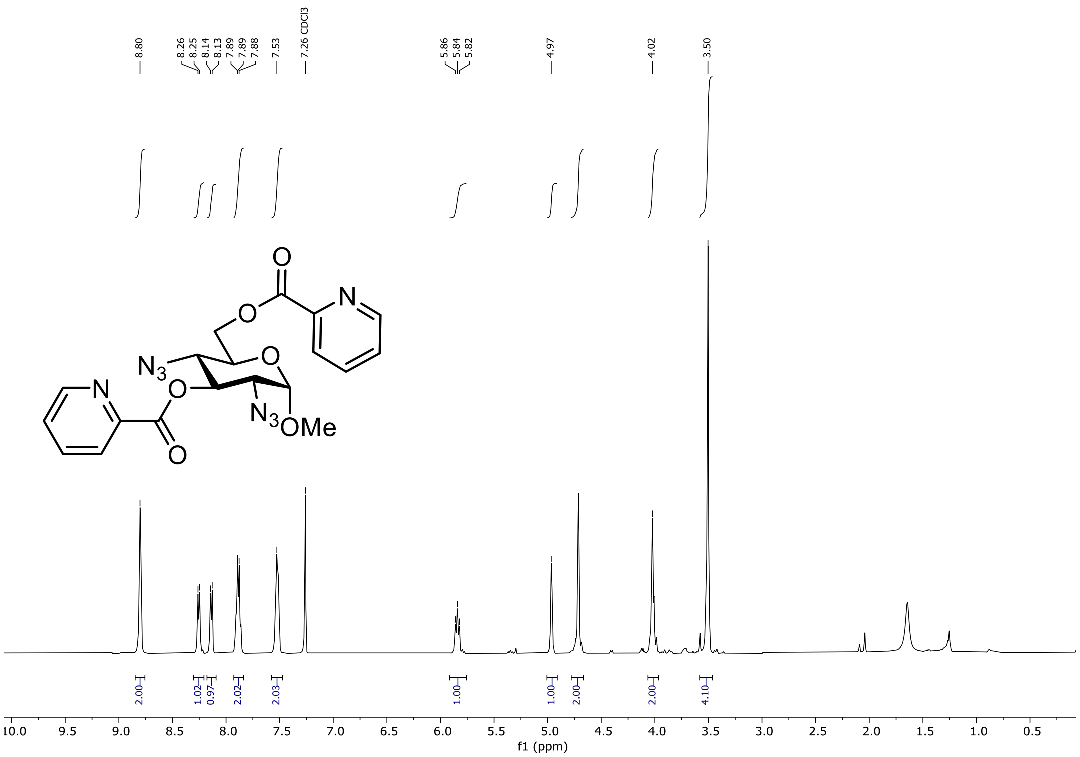

^{13}C{^{1}H}-NMR (126 MHz) spectrum of compound **18α** in chloroform-*d*.

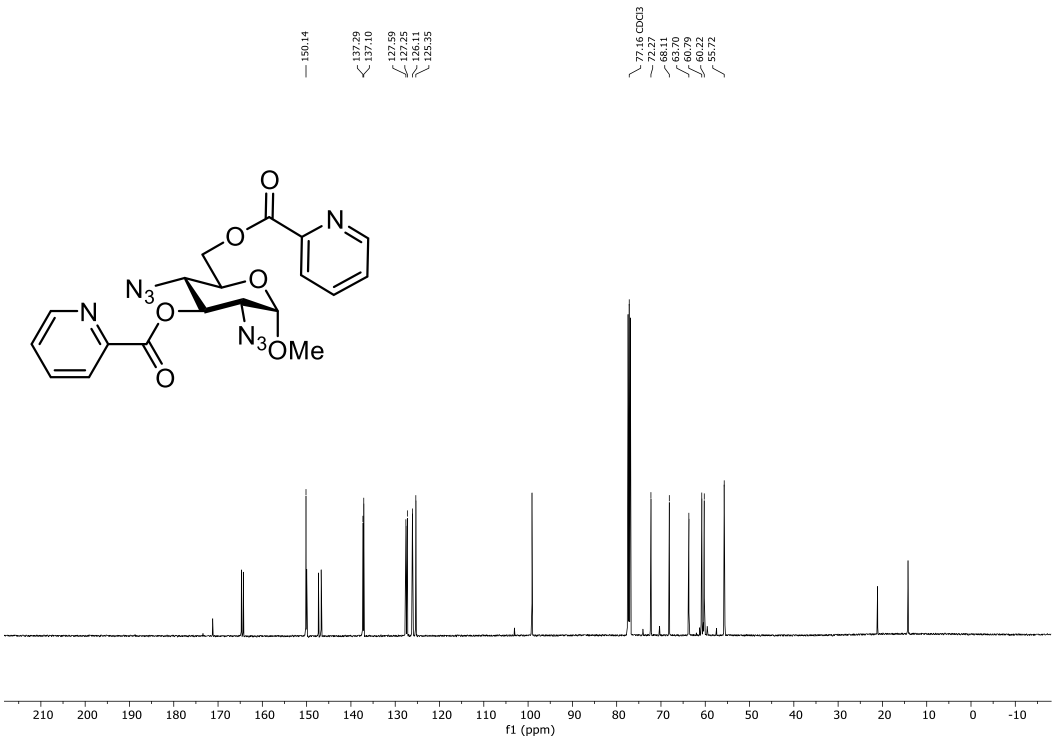

COSY spectrum of compound **18α** in chloroform-*d*.

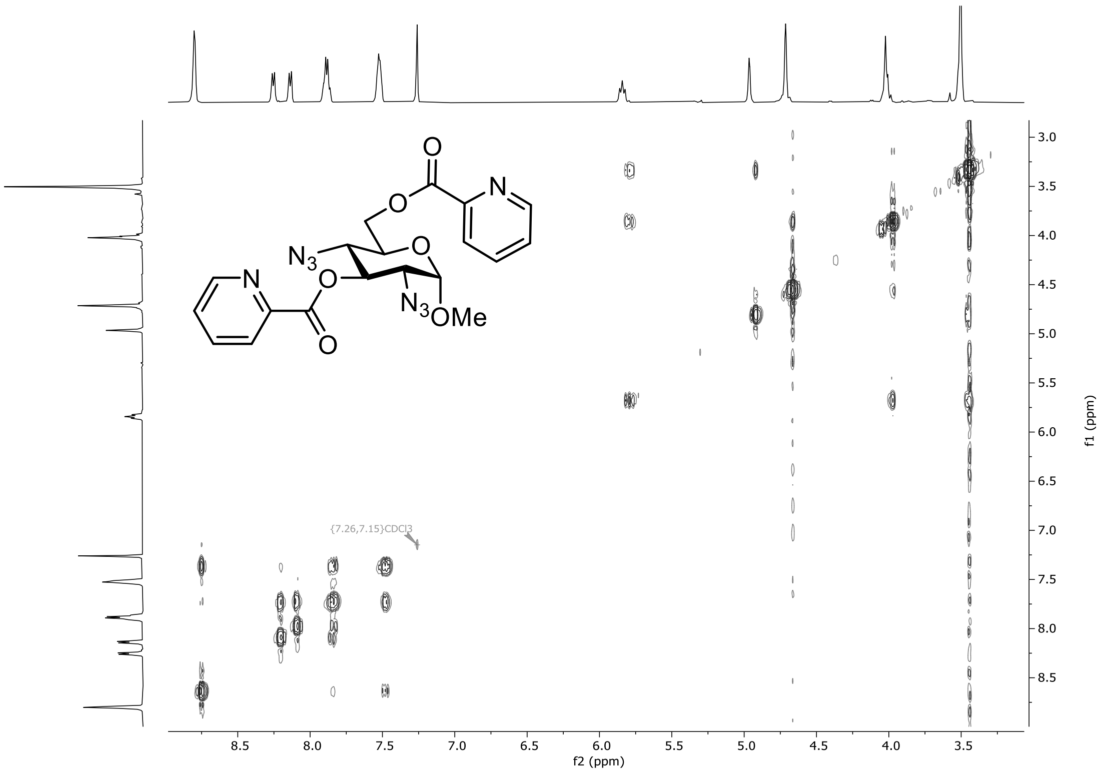

HSQC spectrum of compound **18α** in chloroform-*d*.

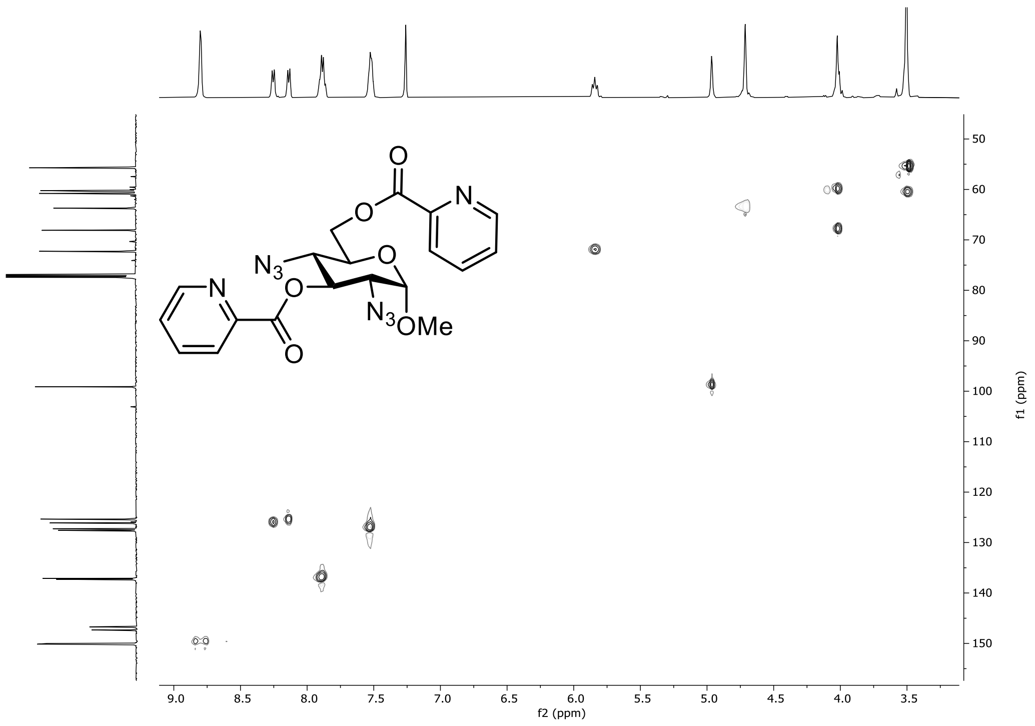

^{1}H-NMR (500 MHz) spectrum of compound **18β** in chloroform-*d*.

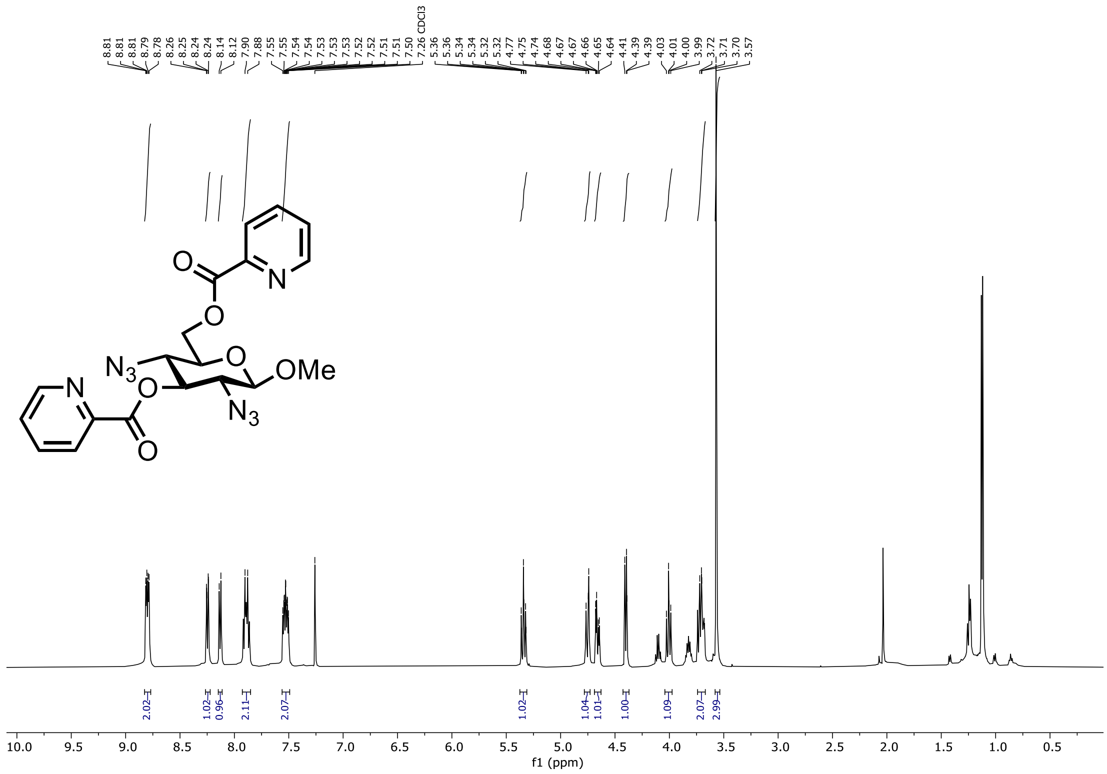

^{13}C{^{1}H}-NMR (126 MHz) spectrum of compound **18β** in chloroform-*d*.

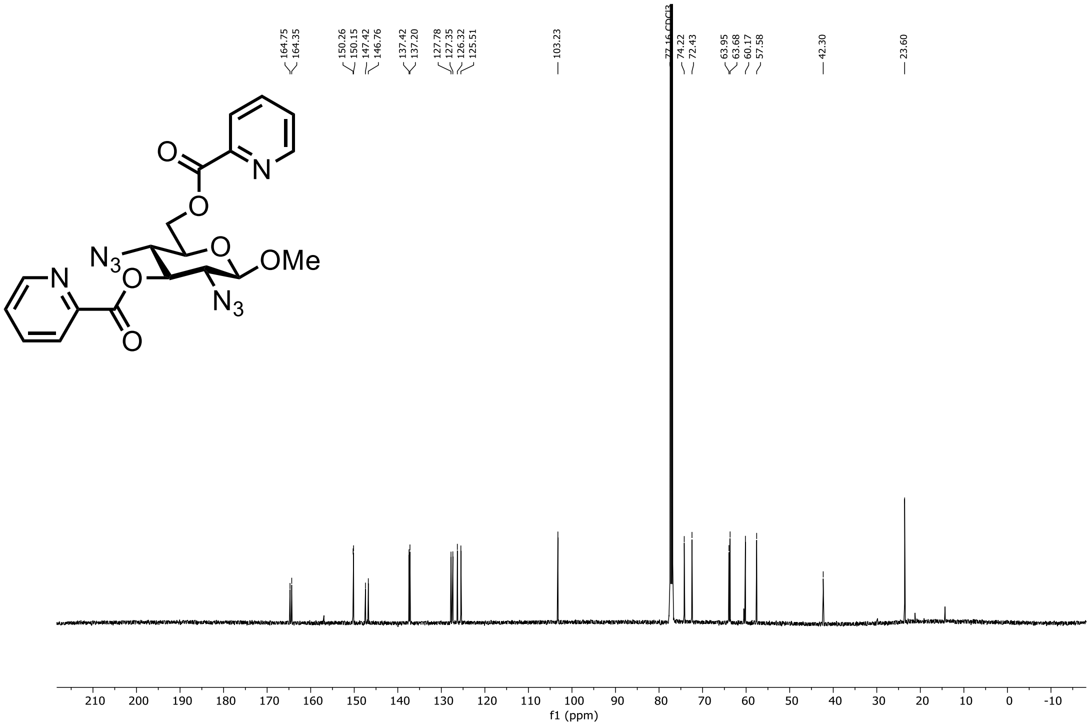

COSY spectrum of compound **18β** in chloroform-*d*.

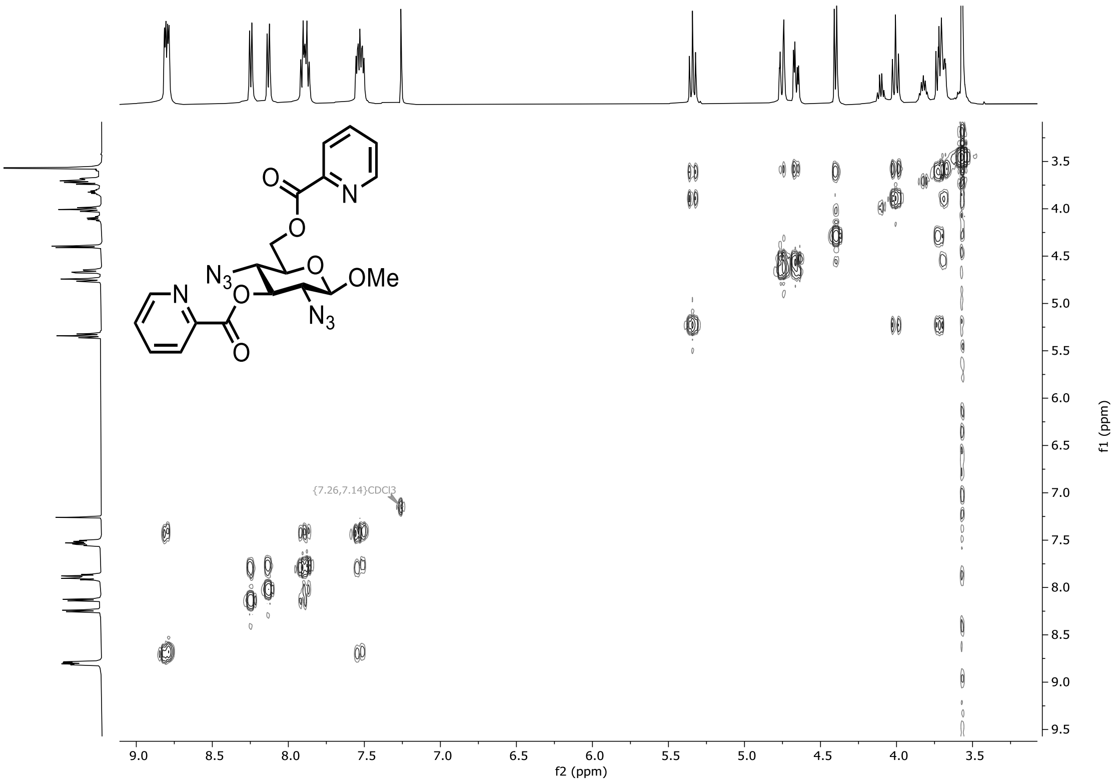

HSQC spectrum of compound **18β** in chloroform-*d*.

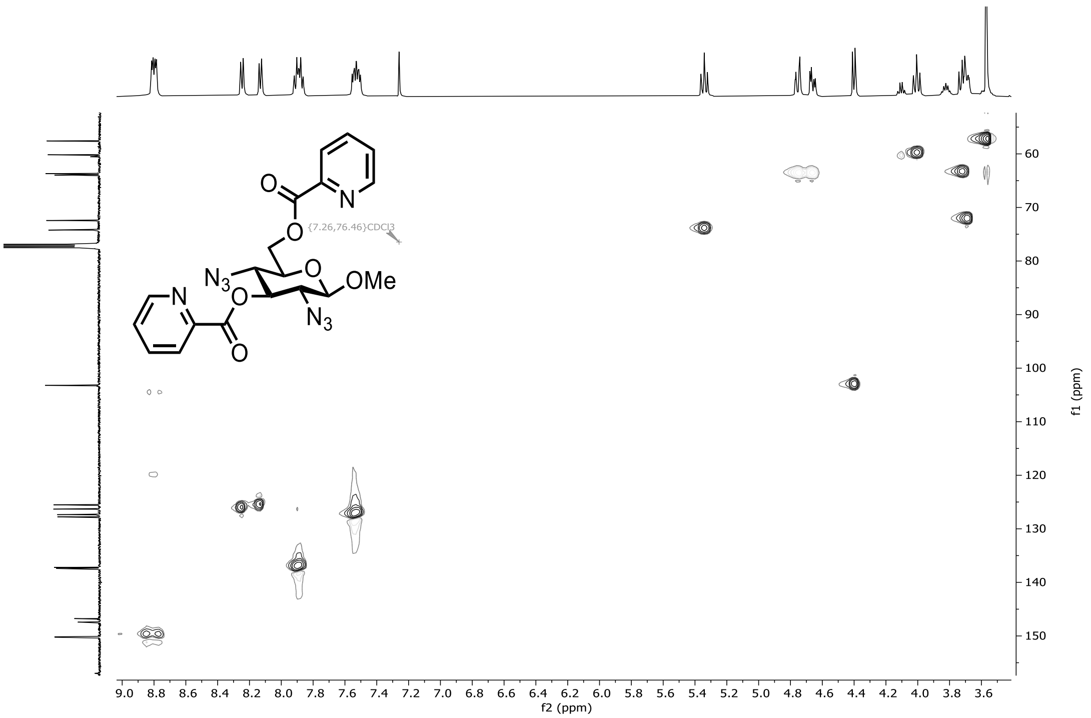

^{1}H-NMR (500 MHz) spectrum of compound **19** in acetonitrile-d_3.

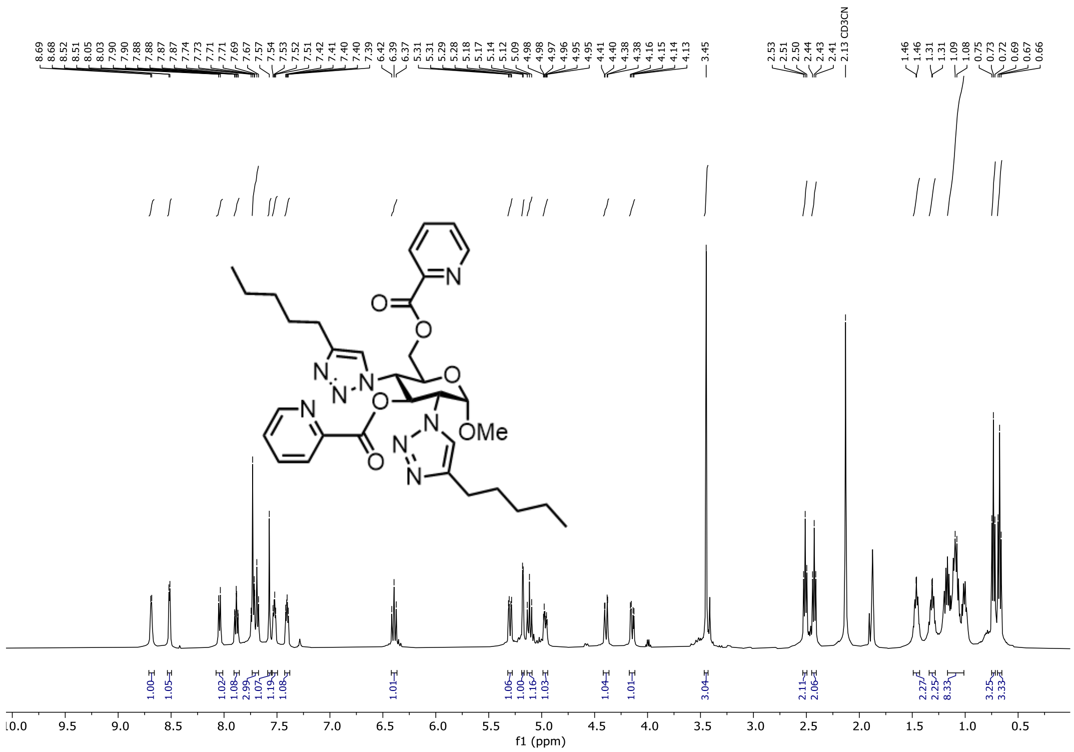

^{13}C{^{1}H}-NMR (126 MHz) spectrum of compound **19** in acetonitrile-d_3.

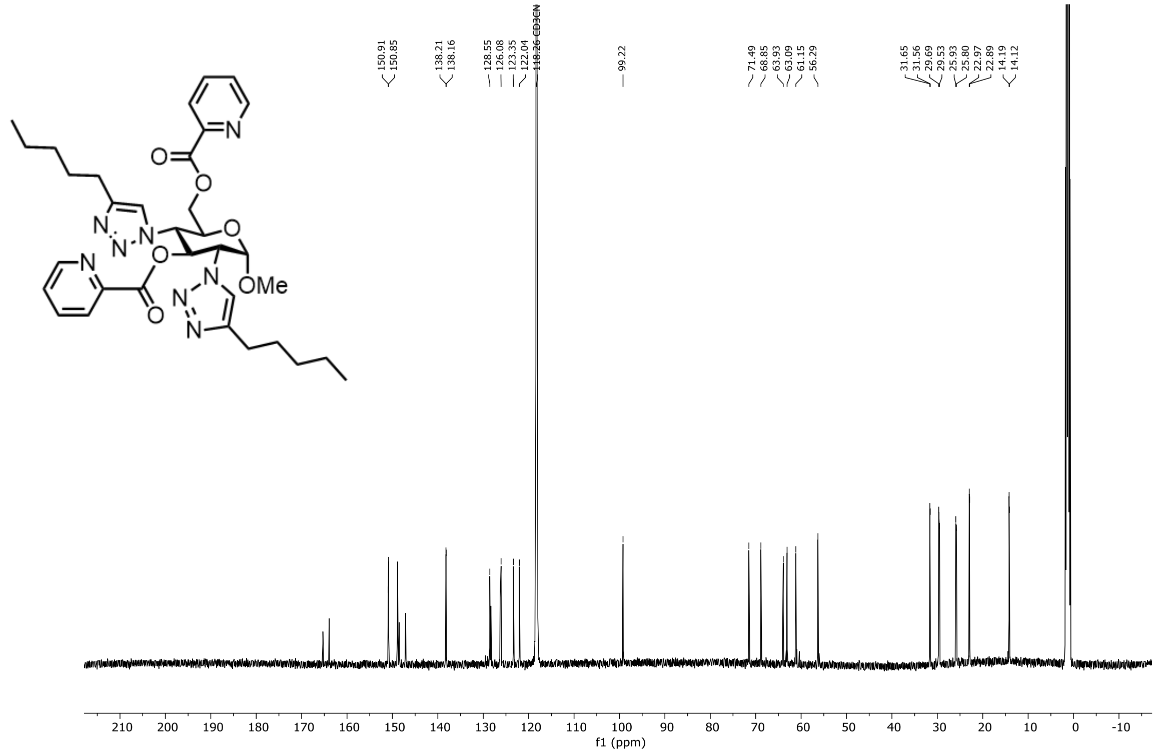

COSY spectrum of compound **19** in acetonitrile-d_3.

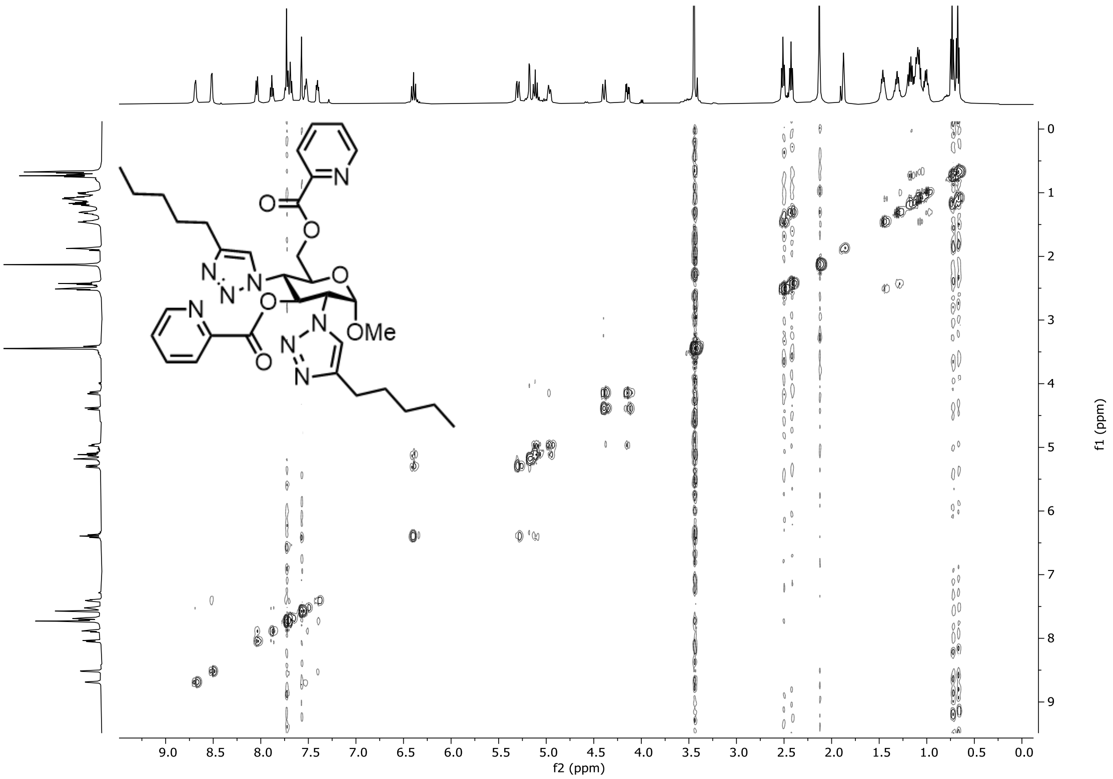

HSQC spectrum of compound **19** in acetonitrile-d_3.

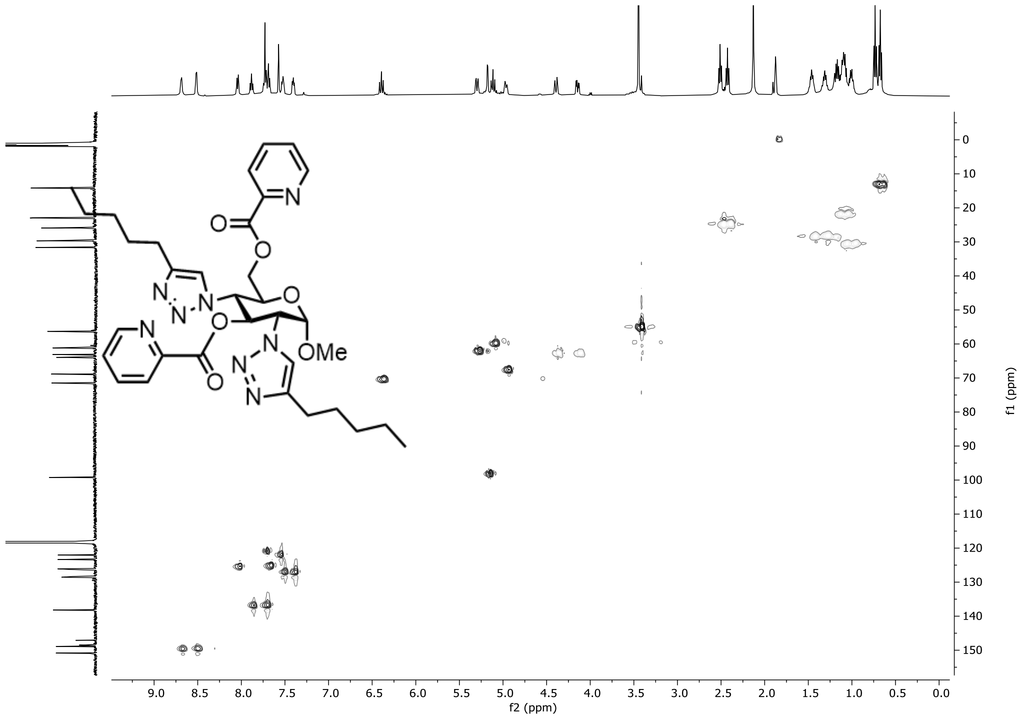

Supplement: File 1 — Experimental procedures and spectroscopic data. [file Beilstein_J_Org_Chem-16-2788-s001.pdf]
